# Probing the changes in gene expression due to α-crystallin mutations in mouse models of hereditary human cataract

**DOI:** 10.1371/journal.pone.0190817

**Published:** 2018-01-16

**Authors:** Usha P. Andley, Eric Tycksen, Brittney N. McGlasson-Naumann, Paul D. Hamilton

**Affiliations:** 1 Department of Ophthalmology and Visual Sciences, Washington University School of Medicine, St. Louis, Missouri, United States of America; 2 Genome Technology Access Center, Washington University in St. Louis, St. Louis, Missouri, United States of America; University of Missouri Columbia, UNITED STATES

## Abstract

The mammalian eye lens expresses a high concentration of crystallins (α, β and γ-crystallins) to maintain the refractive index essential for lens transparency. Crystallins are long-lived proteins that do not turnover throughout life. The structural destabilization of crystallins by UV exposure, glycation, oxidative stress and mutations in crystallin genes leads to protein aggregation and development of cataracts. Several destabilizing mutations in crystallin genes are linked with human autosomal dominant hereditary cataracts. To investigate the mechanism by which the α-crystallin mutations *Cryaa*-R49C and *Cryab*-R120G lead to cataract formation, we determined whether these mutations cause an altered expression of specific transcripts in the lens at an early postnatal age by RNA-seq analysis. Using knock-in mouse models previously generated in our laboratory, in the present work, we identified genes that exhibited altered abundance in the mutant lenses, including decreased transcripts for *Clic5*, an intracellular water channel in *Cryaa*-R49C heterozygous mutant lenses, and increased transcripts for *Eno1b* in *Cryab*-R120G heterozygous mutant lenses. In addition, RNA-seq analysis revealed increased histones H2B, H2A, and H4 gene expression in *Cryaa*-R49C mutant lenses, suggesting that the αA-crystallin mutation regulates histone expression via a transcriptional mechanism. Additionally, these studies confirmed the increased expression of histones H2B, H2A, and H4 by proteomic analysis of *C*r*yaa*-R49C knock-in and *Cryaa*;*C*ryab gene knockout lenses reported previously. Taken together, these findings offer additional insight into the early transcriptional changes caused by *Cryaa* and *Cryab* mutations associated with autosomal dominant human cataracts, and indicate that the transcript levels of certain genes are affected by the expression of mutant α-crystallin *in vivo*.

## Introduction

The formation of cataracts is the most common cause of vision loss, accounting for 51% of the cases of blindness worldwide [1]. To this end, cataract surgeries cost the U.S. Medicare system approximately $5 billion annually [2, 3]. Epidemiological studies have shown that the pathogenesis of human cataracts involves genetic, environmental, and other disease-associated risk factors, and genetic factors account for 50% of childhood cataract cases [4]. The crystallin protein family comprises 90% of proteins found in the lens, and these proteins play a key role in lens transparency. Prior research has led to the identification of point mutations in the genes encoding α-, β-, and γ-crystallins, leading to the development of a hereditary form of human cataracts either at birth or at an early age [5]. The α-crystallin protein is an aggregate of the αA- and αB-crystallin polypeptides that are expressed in epithelial and fiber cells in the lens. Furthermore, human patients harboring single point mutations in αA- and αB-crystallin genes develop hereditary cataracts [6]. Accordingly, functional studies of the formation of hereditary cataracts will provide important information regarding the etiology of age-related cataracts. Thus, to investigate the underlying etiology of hereditary cataracts, we used embryonic stem cell-based technologies to generate knock-in mice expressing proteins harboring either the αA-R49C or αB-R120G α-crystallin mutations, which are associated with human autosomal dominant hereditary cataracts [7, 8]. These mice develop cataracts at an early age and serve as important tools for understanding the disease process [9, 10].

Histones play a central role in the packaging of DNA into nucleosomes, comprise the basic building block of chromatin, and are involved in transcriptional regulation [11–13]; however, very little is known about the specific role of histones in the lens [14, 15]. The expression of histone transcripts decreases in lens epithelial cells exposed to UV radiation, suggesting a response to stress [16]. Also, a recent study demonstrated that the specific acetylation of histone H3 is required for normal lens induction in the embryonic mouse eye at stage E9.5 [14]. Furthermore, αB- and αA-crystallin are first detected at embryonic day 9.5 and 10.5 respectively [17], indicating that these proteins may play a role in lens development. Previously, we demonstrated an increase in the abundance of histones H2B and H4 in lens epithelial cells that express the αA-crystallin R116C mutation [18]. In addition, we observed upregulation of histones at an early age in *Cryaa*;*Cryab* gene knock-out (DKO) and *Cryaa*-R49C homo knock-in mouse lenses [19, 20]. Proteomics analysis of 2-day-old DKO and *Cryaa*-R49C knock-in mouse lenses revealed an increased abundance of histones H2A, H2B fragment, and H4. Furthermore, we discovered that the mutant αA-R49C protein is distributed mainly in the nucleus of the lens, where this protein may bind and sequester histones [7]. Collectively, these findings strongly suggest a functional relationship between histones and α-crystallin. Specifically, an increase in histones may be indicative of an increase in nucleosome density in lenses from the αA-R49C mutant mice, and a functional increase in the histone/DNA ratio may lead to increased amounts of heterochromatin.

The use of RNA-seq technology could be a viable approach for obtaining information regarding the expression of histones in normal and cataractous lenses. At present, whether all core histones (H2A, H2B, H3, and H4) or only specific histones are regulated by α-crystallin mutations is not known [21, 22]. Global changes in all major core histone species would indicate that α-crystallin mutations and cataract formation affect histone expression and that the histones are protected by the chaperone function of α-crystallin. Conversely, a lack of change in histone transcripts in the α-crystallin mutant knock-in lenses would suggest that the previously reported increased abundance of histones resulted from increased apoptosis and subsequent release of histones from chromatin.

Currently, it remains unclear whether α-crystallin serves as a transcriptional inhibitor of histones. Thus, these studies of αA-crystallin and histones will determine whether the expression of histones is affected by the reduced solubility of the αA-R49C mutant protein *in vivo* [7, 9].

## Methods

### Animals and lenses

*Cryaa*-R49C knock-in mice and *Cryab*-R120G knock-in mice were generated at the Department of Ophthalmology and Visual Sciences at Washington University School of Medicine by stem cell-based techniques described previously [9, 10, 23]. Wild-type (WT), heterozygous (het) and homozygous (homo) knock-in mice carrying the αA-crystallin R49C mutation (*Cryaa*-R49C) or the αB-crystallin R120G mutation (*Cryab*-R120G) on a C57Bl/6J background were genotyped by PCR-based methods. C57BL/6J mice were used as age-matched controls Animal care, breeding and genotyping were performed by trained veterinary staff at the Mouse Genetics Core at Washington University. All experimental procedures conducted using the mice were reviewed and approved by the Institutional Animal Care and Use Committee at Washington University. Adult mice were sacrificed by CO_2_ inhalation. Postnatal mice were sacrificed by CO_2_ inhalation followed by cervical dislocation.

### RNA isolation and purification

Mouse eyes were dissected, and the lenses were collected as described previously [10]. RNA was extracted from mouse lenses using protocols from the Tissue Procurement Center at Washington University. The mice used for RNA isolation were 2-day-old αA-R49C knock-in and 14-day-old αB-R120G knock-in mice. RNA was isolated from three groups of lenses for each genotype. Multiple lenses were combined for each biological replicate. The number of animals in each group used for RNA isolation is shown in S1 Table.

### RNA-seq library preparation

Library preparation was performed using a Clontech poly-A kit according to the manufacturer’s protocol. The resulting cDNA ends were rendered blunt, an A base was added to the 3’ ends, and Illumina sequencing adapters were ligated to the ends. The ligated fragments were then amplified for 12 cycles using primers that incorporated unique index tags, and the resulting fragments were sequenced on an Illumina HiSeq 2500 sequencing system using single reads that extended 50 bases and targeted 30M reads per sample.

### RNA-seq data acquisition, quality control, and processing

RNA-seq reads were aligned to the GRCm38.76 assembly from Ensembl using Spliced Transcripts Alignment to Reference (STAR) version 2.0.4b [24]. The gene counts were derived from the number of uniquely aligned unambiguous reads that were detected using Subread-featureCounts version 1.4.5 [25], and the transcript counts were produced using Sailfish version 0.6.3. The sequencing performance was assessed based on the total number of aligned reads, total number of uniquely aligned reads, genes and transcripts detected, ribosomal fraction known junction saturation, and read distribution over known gene models using RSeQC version 2.3 [26]. All gene-level and transcript counts were then imported into the R Bioconductor edgeR package [27, 28]. The trended mean of M values (TMM) were normalized to account for differences in library size, and the genes or transcripts not expressed in any sample were excluded from further analysis. The raw counts and fastq.gz files are available at NCBI GEO accession number GSE98027.

The performance of the samples was assessed using a Spearman's rank-order correlation matrix, and multi-dimensional scaling plots and generalized linear models with robust dispersion estimates were created to test for differential expression at both the gene and transcript levels. The fit of the trended and tagwise dispersion estimates were then plotted to confirm the proper fit of the observed mean to the variance relationship in which the tagwise dispersions were equivalent to the biological coefficients of variation in each gene. Differentially expressed genes and transcripts were then filtered for false discovery rate (FDR)-adjusted *p*-values ≤0.05.

To enhance the biological interpretation of the large set of transcripts, genes and transcripts were grouped based on known Kyoto Encyclopedia of Genes and Genomes (KEGG) biological interactions and pathways. We used the R Bioconductor package GAGE [29] to examine the log 2 fold-changes reported by Limma to determine if genes expressed in each contrast, regardless of statistical significance, were up or down regulated in comparison to global background log 2 fold-changes as well as tests for perturbations of log 2 fold-changes within pathways. For any pathway that was significantly up or down regulated or perturbed with a *p*-value less than or equal to 0.05, annotated KEGG graphs were downloaded and rendered with the R/Bioconductor package Pathview [30] so that gene and protein complexes were color coded by the mean log 2 fold-change with a rescaled minimum of -2 and maximum of 2 for ease of interpretation. Heat maps of genes with altered expression in knock-in mutant lenses were displayed by the R package heatmap3 [31].

### Real-time PCR

All real-time quantitative PCR (qPCR) analyses were performed by the Genome Technology Access Center using RNA samples extracted from the lenses of wild-type (WT), *Cryaa*-R49C, and *Cryab*-R120G mice. The primers used for the qPCR analyses are listed in S2 Table.

### Gel permeation chromatography (GPC)

The murine lenses were homogenized, and water-soluble proteins were extracted as described previously [10]. The molecular weights of the extracted proteins were determined from the light scatter data, and the concentrations were calculated based on the refractive index data. In the studies involving water-soluble proteins from WT and knock-in mouse lenses, 100 μl of water-soluble lens proteins were separated in succession on G3000 PWXL and G5000 PWXL size exclusion chromatography columns (Tosoh Bioscience LLC, Prussia, PA) in line with the Viscotek TDA 302 triple detector array system, which measured UV absorption, refractive index (RI), multi-angle light scattering, and viscosity (Viscotek/Malvern). The GPC system was equipped with a VE-1122 pump and a VE-7510 degasser. The Viscotek OmniSEC software was used to calculate the molecular weight of the crystallin proteins using bovine serum albumin (BSA) and the 92-kDa Pullulan Malvern standards. The protein samples (100 μl) were injected onto the columns, and Dulbecco’s Phosphate-Buffered Saline (PBS) Modified buffer (0.5x) without Ca^2+^ or Mg^2+^ was used as the mobile phase at a flow rate of 0.8 ml/min at 25°C. The protein concentration was calculated based on the refractive index using a dn/dc of 0.185 for both the BSA standard and the crystallin proteins.

## Results

Previous proteomics analyses have focused on the effects of mutations in lens proteins that are associated with the development of cataracts in lenses obtained from *Cryaa*-R49C and *Cryab*-R120G mutant mice at early postnatal ages. Our current work, however, examined changes in the expression of protein-coding genes and non-coding genes in these lenses *in vivo*. The lens samples utilized in the current RNA-seq analysis were whole lenses collected from 2-day-old WT, *Cryaa*-R49C-het, and *Cryaa*-R49C-homo mice that were the same age as the mice used in the proteomics analyses. In addition, lens samples obtained from 14-day-old WT, *Cryab*-R120G-het, and *Cryab*-R120G-homo mice were also examined, because cataracts appear at an older age in the *Cryab*-R120G knock-in mice than in the *Cryaa*-R49C knock-in mice. Both of these ages represent early stages of cataract formation in these two models. We analyzed three biological replicates for each of the genotypes described above, and each replicate comprised 4 to 17 whole lenses.

The RNA-seq analysis resulted in approximately 29–51 million raw sequence reads for each of the 18 cDNA libraries generated from the pooled murine lenses (S3 Table). On average, 88–94% of the trimmed reads mapped to the mouse reference genome. The lenses obtained from *Cryaa*-R49C mice contained 38,000–42,000 transcripts, and the lenses from *Cryab*-R120G mice contained 28,000–31,000 transcripts. Also, the expression of 19,000 and 17,000 genes was detected in lenses obtained from *Cryaa*-R49C and *Cryab*-R120G mice, respectively. The ribosomal fraction was ≤1% in our samples, but the lenses contained a high amount of long non-coding RNAs (lncRNAs), although the basis of this observation remains unknown (S1 and S2 Figs). The end bias plots (S3 Fig) illustrate the placement of the reads with fewer reads at the 5’ end, most likely due to the large number of transcripts sequenced and the high level of lncRNAs in these samples (S1 Fig). To determine the similarity between expression profiles, we used Pearson correlation plots (S4 Fig). The correlation values indicate closely similar expression values for transcripts between biological replicates.

Sailfish was used to estimate the number of transcripts and splice isoforms using an expectation-maximization algorithm to account for bias correction. The STAR summary is shown in S3 Table. The STAR summary indicated that the average input read length was 49, which is very good. The number of splice junctions, which were calculated using the ratio of the number of spliced reads to the total number of reads from the alignment summary, were limited and accounted for 0.1%. The majority of reads that were unmapped were too short to be mapped.

**Fig 1:**
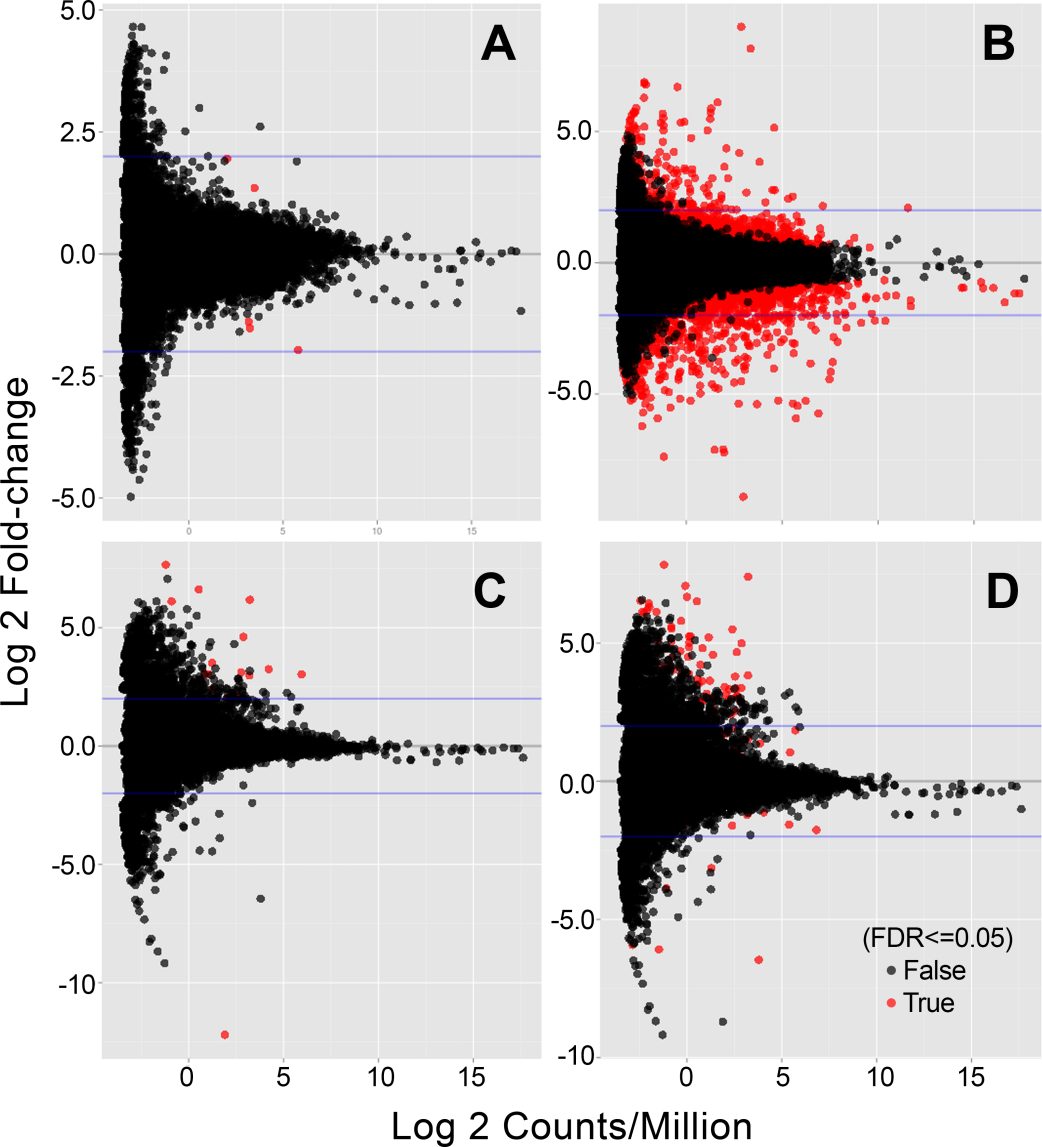
MA plots for genes that were altered in lenses obtained from *Cryaa*-R49C or *Cryab-*R120G mutant knock-in mice as compared with age-matched WT mice. (A) 2-day-old *Cryaa*-R49C-het, (B) 2-day-old *Cryaa*-R49C-homo, (C) 14-day-old *Cryab*-R120G-het, and (D) 14-day-old *Cryab*-R120G-homo mice. The x-axis indicates the average expression over all samples in the *Cryaa* (or *Cryab*) lenses. The y-axis represents the log 2-fold change of normalized counts between the mutant and WT samples.

At the gene level, the unadjusted *p*-value of ≤0.05 and the Benjamini-Hochberg FDR *p*-value were examined, and the genes were annotated using Ensembl. The log ratio and mean average (MA) plots indicate differentially expressed genes (FDR <0.05; red dots) identified in lenses collected from 2-day-old *Cryaa*-WT, *Cryaa*-R49C het, and *Cryaa*-R49C homo mice (Fig 1A and 1B, respectively), and from lenses collected from 14-day-old *Cryab*-WT, *Cryab*-R120G-het, and *Cryab*-R120G-homo mice (Fig 1C and 1D, respectively). Each gene is represented by a dot, and differentially expressed genes are represented by red dots (Fig 1). The lenses from *Cryaa*-R49C-het mice exhibited very few differentially expressed genes (red dots) compared with lenses from WT mice (see also Table 1). Conversely, a comparison of lenses collected from the *Cryaa*-R49C-homo and *Cryab*-R120G-homo versus those from WT mice revealed the differential expression of several genes, as shown in the MA plots (Fig 1B and 1D). The lower number of gene changes observed in *Cryaa*-R49C-het, *Cryab*-R120G-het, and *Cryab*-R120G-homo mouse lenses suggest the effects of the mutations on gene expression in the pre-cataractous lens.

**Table 1:** List of genes with altered abundance in *Cryaa*-R49C-het mouse lenses.

Entrez gene ID	Gene name	Protein ID	Linear fold change	Log fold change	*p-*value	FDR
224796	*Clic5*	Chloride intracellular channel 5	-3.909	-1.967	1.92E-13	5.14E-09
11568	*Aebp1*	AE binding protein 1	-2.614	-1.386	4.08E-07	5.11E-03
58185	*Rsad2*	radical S-adenosyl methionine domain containing 2	-2.867	-1.519	6.08E-07	5.11E-03
21648	*Dynlt1b*	dynein light chain Tctex-type 1B	3.867	1.951	7.65E-07	5.11E-03
19736	*Rgs4*	regulator of G-protein signaling 4	2.554	1.353	3.14E-06	1.68E-02

Differentially expressed genes in the WT, *Cryaa*-R49C-het, and *Cryaa*-R49C-homo lenses were filtered based on the highest fold change and significant *p*-values (Table 2 and S4 Table), leading to the identification of genes that were significantly altered in lenses from *Cryaa*-R49C and *Cryab*-R120G mice. The RNA-seq analysis of lenses from 2-day-old *Cryaa*-R49C-het mice revealed significant changes in the expression of only five genes compared with analysis of lenses from WT mice. These altered genes included *Clic5* (-3.9-fold), *Aebp1* (-2.6-fold), and *Rsad2* (-2.9-fold), which were decreased in the *Cryaa*-R49C-het mouse lenses, as well as *Dynlt1b* (3.9-fold) and *Rgs4* (2.6-fold), which were increased in the *Cryaa*-R49C-het mouse lenses. Because the magnitude of the expression changes was rather small, the genes were not analyzed by qPCR. Conversely, RNA-seq analysis of lenses from *Cryaa*-R49C-homo mice revealed changes in the expression of 2450 genes with an FDR <0.05 compared with WT mouse lenses (S4 Table). The genes that exhibited increased expression included *Ddit3* (35.3-fold and 34.3-fold by RNA-seq and qPCR, respectively), *Chac1* (285-fold and 276-fold by RNA-seq and qPCR, respectively), and *Trib3* (509-fold and 298-fold by RNA-seq and qPCR, respectively) (S4 Table). Genes that were decreased in *Cryaa*-R49C-homo mouse lenses compared to WT lenses included *Tcp11* (-17.78-fold and -5-fold by RNA-seq and qPCR, respectively), *Stx11* (-31.98-fold and -6.25-fold by RNA-seq and qPCR, respectively), and *Hspb1* (-69.09 and -50-fold by RNA-seq and qPCR, respectively).

**Table 2:** List of histone genes with altered abundance in *Cryaa*-R49C-homo mouse lenses.

Entrez gene ID	Gene name	Protein ID	Linear fold change	Log fold change	*p*-value	FDR
50708	Hist1h1c	histone cluster 1, H1c	2.616	1.38762	1.11E-06	6.04E-05
102641229	Hist2h4	histone cluster 2, H4	3.404	1.76761	6.26E-05	1.80E-03
97122	Hist2h4	histone cluster 2, H4	3.404	1.76761	6.26E-05	1.80E-03
319162	Hist3h2a	histone cluster 3, H2a	1.695	0.76152	2.70E-04	5.82E-03
68024	Hist1h2bc	histone cluster 1, H2bc	1.663	0.73402	5.58E-04	1.03E-02
78303	Hist3h2ba	histone cluster 3, H2ba	-1.703	-0.76846	7.40E-04	1.29E-02
319166	Hist1h2ae	histone cluster 1, H2ae	-2.581	-1.36797	2.08E-03	2.80E-02

**Fig 2:**
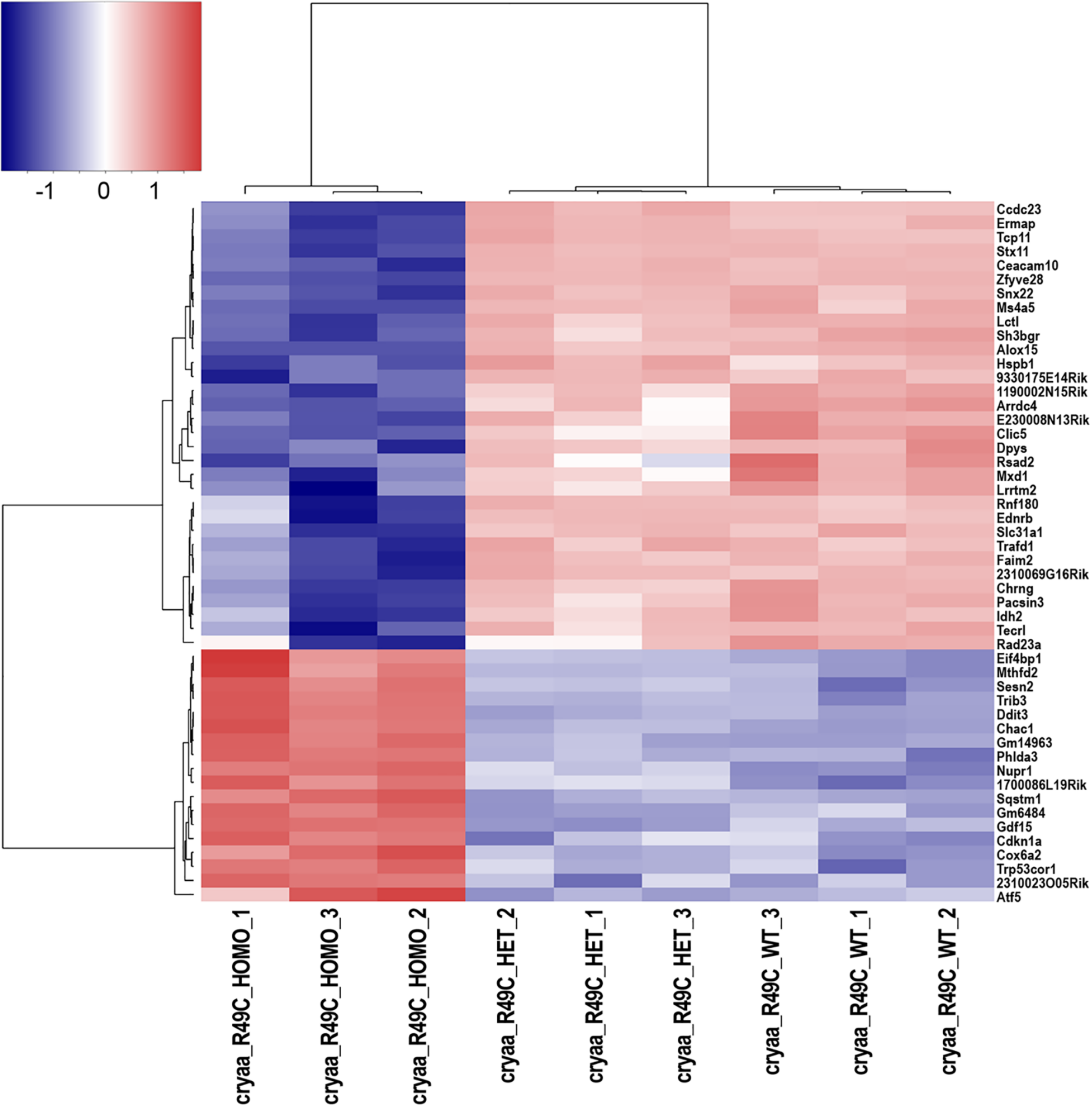
Heat map of genes with altered expression in *Cryaa*-R49C knock-in mutant lenses as displayed by the R package heatmap3 [31]. In order to illustrate relative log 2 fold-changes across samples, the values displayed per gene represent log 2 transformed counts-per-million with a prior count of 2, which were then rescaled to a mean of 0 with a standard deviation of 1. The genes and samples were then re-organized by hierarchical clustering to illustrate the similarity of expression changes within and across genotypes.

The top 30 genes that were upregulated in *Cryaa*-R49C-homo mouse lenses compared with WT mouse lenses included *Mthfd2*, *Phida3*, *Gm14963*, *Sesn2*, *cdkn1a*, *1700086L19Rik*, and *Gdf15* (Fig 2 and S4 Table). The genes that exhibited decreased expression in *Cryaa*-R49C-homo mouse lenses compared with lenses from WT mice included *Hspb1*, *Ccdc23*, *Snx22*, *Ceacam10*, *Ms4a5*, and *Ermap* (Fig 2). Additionally, *Hist2h4* was upregulated in *Cryaa*-R49C-homo mouse lenses compared with lenses from WT mice.

**Fig 3:**
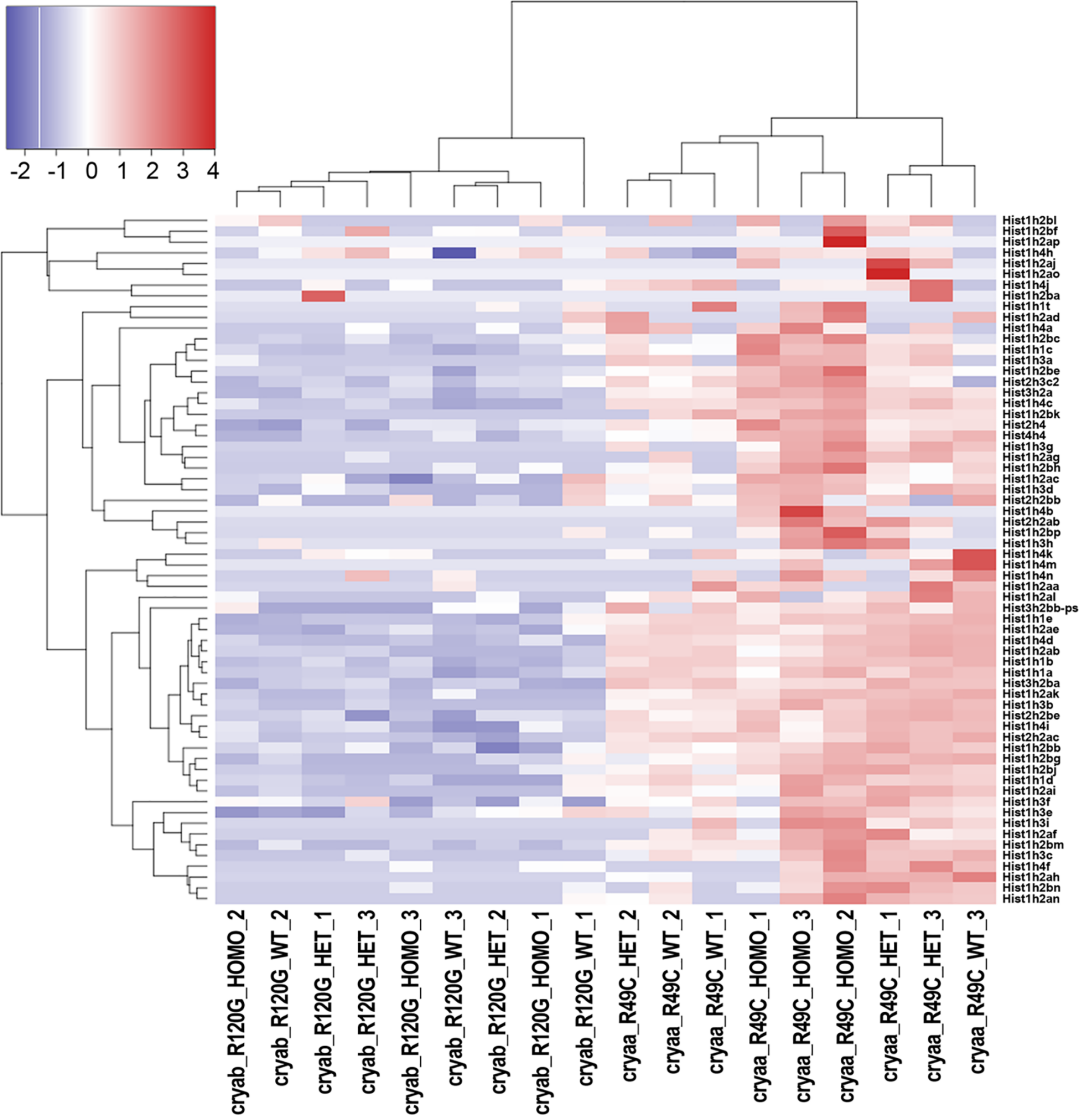
The heat map for change in histone genes in *Cryaa*-R49C and *Cryab*-R120G knock-in mutant lenses as displayed by the R package heatmap3 [31]. In order to illustrate relative log 2 fold-changes across samples, the values displayed per gene represent log 2 transformed counts-per-million with a prior count of 2, which were then rescaled to a mean of 0 with a standard deviation of 1. The genes and samples were then re-organized by hierarchical clustering to illustrate the similarity of expression changes within and across genotypes. In one of the WT samples, the analysis of histone gene expression appeared more like that of the *Cryaa*-R49C-het sample, indicating that variability in the expression of the histone genes can occur even within the same genotype. This outlier was not the result of a sample mix-up, because the non-histone genes exhibited the same fold change and direction of change as the three genotypes.

The effect of the mutation on the expression of histone genes was analyzed in lenses from *Cryaa*-R49C-het and *Cryaa*-R49C-homo mice (Fig 3). The lens libraries contained numerous transcripts for genes that encoded several different histones, and increases in the histone cluster 1 (H1 and H2), histone cluster 2 (H4), and histone cluster 3 (H2) genes were the most notable in lenses from *Cryaa*-R49C-homo mice compared with WT mouse lenses. A block of genes encoding histones was upregulated in *Cryaa*-R49C-homo mouse lenses compared to expression in lenses from WT mice, and other histone genes were upregulated in lenses from both *Cryaa*-R49C-het and *Cryaa*-R49C-homo mice compared to WT mice. Notably, expression of different histone genes was also increased in the *Cryaa*-R49C-het mouse lenses compared to WT mouse lenses. These differentially expressed histone genes included histone cluster 1 (H1 and H2) and histone cluster 3 (H2). The genes shown in the heat maps are those with low adjusted *p*-values and high fold changes (Fig 3 and Table 2).

The RNA-seq analysis likewise revealed that expression of 26 genes was significantly altered in *Cryab*-R120G-het mouse lenses (FDR<0.05; Table 3), and these differentially expressed genes included *Slc6a13* (5.3-fold linear increase), *Ttr* (4.1-fold), *Tfap2b* (24.5-fold), and *Hba-a2* (8.6-fold). These results were validated by qPCR, and a 2.6-fold increase in *Hba-a2*, a 6.8-fold increase in *Ttr*, and a 1.5-fold increase in *Slc6a13* expression were revealed. In lenses from *Cryab*-R120G-homo mice, 103 genes were significantly altered compared to gene expression in WT mouse lenses (S5 Table). Among these, *Slc6a13* was increased 5.7-fold and 1.7-fold based on the RNA-seq and qPCR results, respectively, and *Ttr* was increased 3.8-fold and 5.1-fold based on RNA-seq and qPCR, respectively (S5 Table). No marked differences in the expression or transcription of histone genes were detected among the lenses from *Cryab*-R120G WT and *Cryab*-R120G-het mice. However, transcripts for the variant histone H2A.X increased 2.6-fold in the lenses of *Cryab*-R120G-homo mice as compared with *Cryab*-R120G WT lenses. In addition, our data indicated that histones were downregulated in the lenses from *Cryab*-R120G mice compared with *Cryaa*-R49C mice, perhaps due to the older age of the *Cryab*-R120G mice.

**Table 3:** List of top 30 genes with altered abundance in *Cryab*-R120G-het mouse lenses.

Entrez gene ID	Gene name	Protein ID	Linear fold change	Log fold change	*p*-value	FDR
NA	*Gm11942*	predicted gene 11942	72.5	6.18	6.86E-14	1.83E-09
433182	*Eno1b*	enolase 1B, retrotransposed	8.16	3.0	5.57E-13	7.44E-09
13806	*Eno1b*	enolase 1B, retrotransposed	8.16	3.03	5.57E-13	7.44E-09
14412	*Slc6a13*	solute carrier family 6	5.29	2.40	4.54E-08	4.04E-04
110257	*Hba-a2*	hemoglobin alpha, adult chain 2	8.63	3.11	1.25E-07	8.37E-04
57811	*Rgr*	retinal G protein coupled receptor	8.30	3.05	2.18E-07	1.17E-03
11656	*Alas2*	aminolevulinic acid synthase 2, erythroid	11.4	3.51	3.64E-07	1.62E-03
15122	*Hba-a1*	hemoglobin alpha, adult chain 1	7.90	2.98	6.35E-07	2.42E-03
15129	*Hbb-bs*	hemoglobin, beta adult s chain	8.20	3.04	1.40E-06	4.67E-03
100503605	*Hbb-bs*	hemoglobin, beta adult s chain	8.20	3.04	1.40E-06	4.67E-03
15130	*Hbb-bs*	hemoglobin, beta adult s chain	8.20	3.04	1.40E-06	4.67E-03
13190	*Dct*	dopachrome tautomerase	4.08	2.03	2.16E-06	5.79E-03
114142	*Foxp2*	forkhead box P2	98.3	6.62	2.17E-06	5.79E-03
20431	*Pmel*	premelanosome protein	4.34	2.12	2.97E-06	6.62E-03
22139	*Ttr*	transthyretin	4.13	2.05	2.83E-06	6.62E-03
21419	*Tfap2b*	transcription factor AP-2 beta	24.5	4.62	3.74E-06	7.68E-03
15130	*Hbb-bt*	hemoglobin, beta adult t chain	9.52	3.25	6.09E-06	1.09E-02
101488143	*Hbb-bt*	hemoglobin, beta adult t chain	9.52	3.25	6.09E-06	1.09E-02
15129	*Hbb-bt*	hemoglobin, beta adult t chain	9.52	3.25	6.09E-06	1.09E-02
NA	*Gm15610*	predicted gene 15610	200.2	7.66	6.00E-06	1.09E-02
12842	*Col1a1*	collagen, type I, alpha 1	3.96	1.98	1.88E-05	3.14E-02
14282	*Fosb*	FBJ osteosarcoma oncogene B	-4.71E+03	-1.22E+01	2.11E-05	3.31E-02
22228	*Ucp2*	uncoupling protein 2 (mitochondrial, proton carrier)	3.08	1.62	3.54E-05	4.97E-02
317758	*Gimap9*	GTPase, IMAP family member 9	69.1	6.11	3.43E-05	4.97E-02
19699	*Reln*	reelin	6.76	2.76	4.51E-05	6.02E-02
20150	*Slc1a1*	solute carrier family 1	27.6	4.79	5.83E-05	7.42E-02
83961	*Nrg4*	neuregulin 4	-2.87	-1.52	6.71E-05	8.15E-02
67729	*Mansc1*	MANSC domain containing 1	18.0	4.17	8.37E-05	9.72E-02
20512	*Slc1a3*	solute carrier family 1	7.18	2.84	9.78E-05	9.76E-02
20476	*Six6*	sine oculis-related homeobox 6	5.67	2.50	1.06E-04	9.76E-02

**Fig 4:**
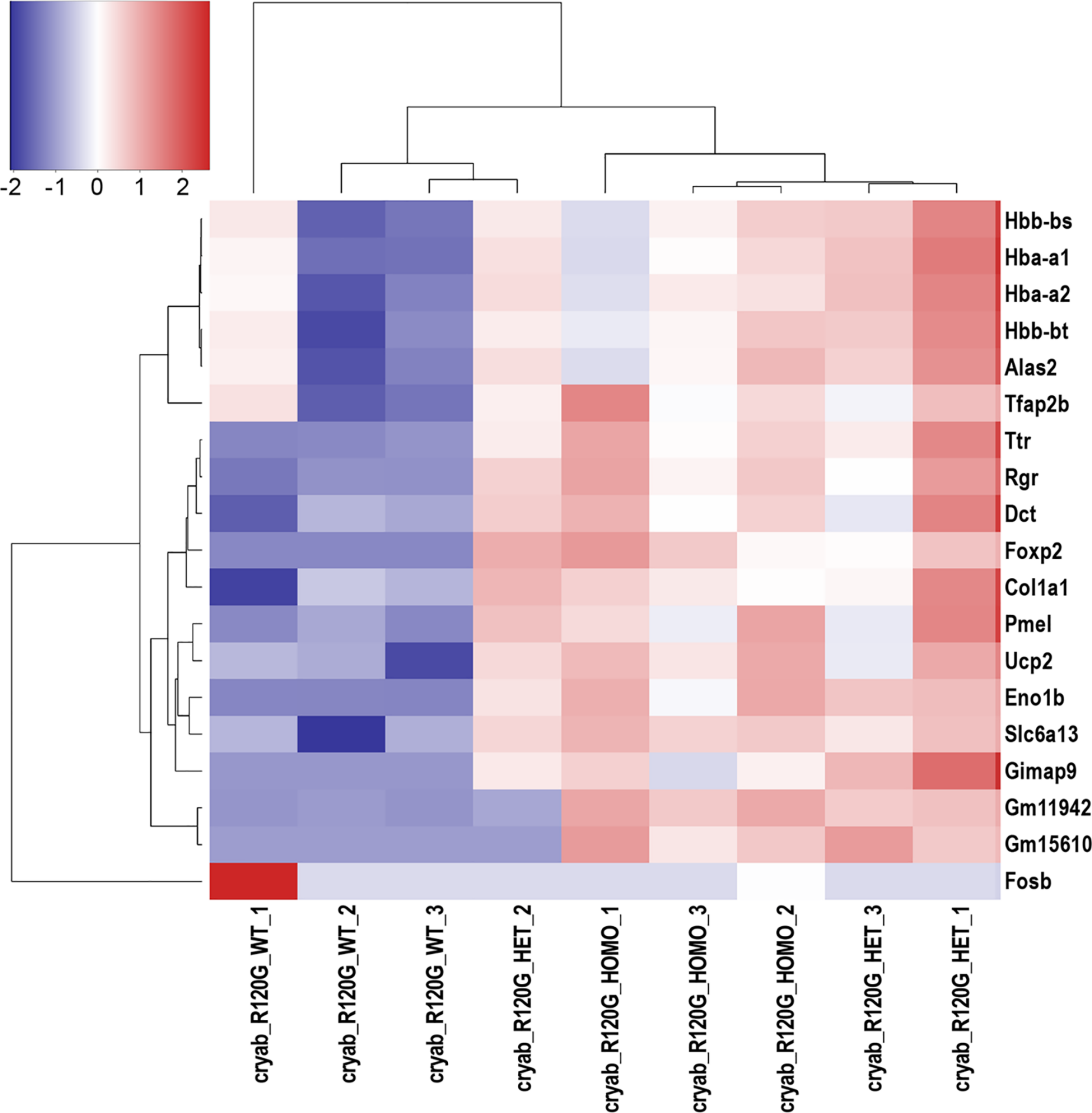
Heat maps of genes with altered expression in *Cryab*-R120G knock-in mutant lenses as displayed by the R package heatmap3 [31]. In order to illustrate relative log 2 fold-changes across samples, the values displayed per gene represent log 2 transformed counts-per-million with a prior count of 2, which were then rescaled to a mean of 0 with a standard deviation of 1. The genes and samples were then re-organized by hierarchical clustering to illustrate the similarity of expression changes within and across genotypes.

The top 30 genes that were differentially expressed in lenses from *Cryab*-R120G-het and *Cryab*-R120G-homo mice, when compared with *Cryab*-R120G-WT mice included the downregulation of *Hbb-ba*, *Hbb-b1*, *Hba-a1*, *Hba-a2*, *Alas2*, *Slc6a13*, and *Tfapb2* (Fig 4). Marked upregulation of *FosB* was detected in one of the three *Cryab*-R120G-WT samples despite no apparent changes in hemoglobin gene expression, indicating the possibility that this sample could be an outlier. Only one histone transcript *H2afx*, for the variant histone H2A.X, showed a significant 2.9-fold increase in the lenses of *Cryab*-R120G homo mice (S5 Table).

**Fig 5:**
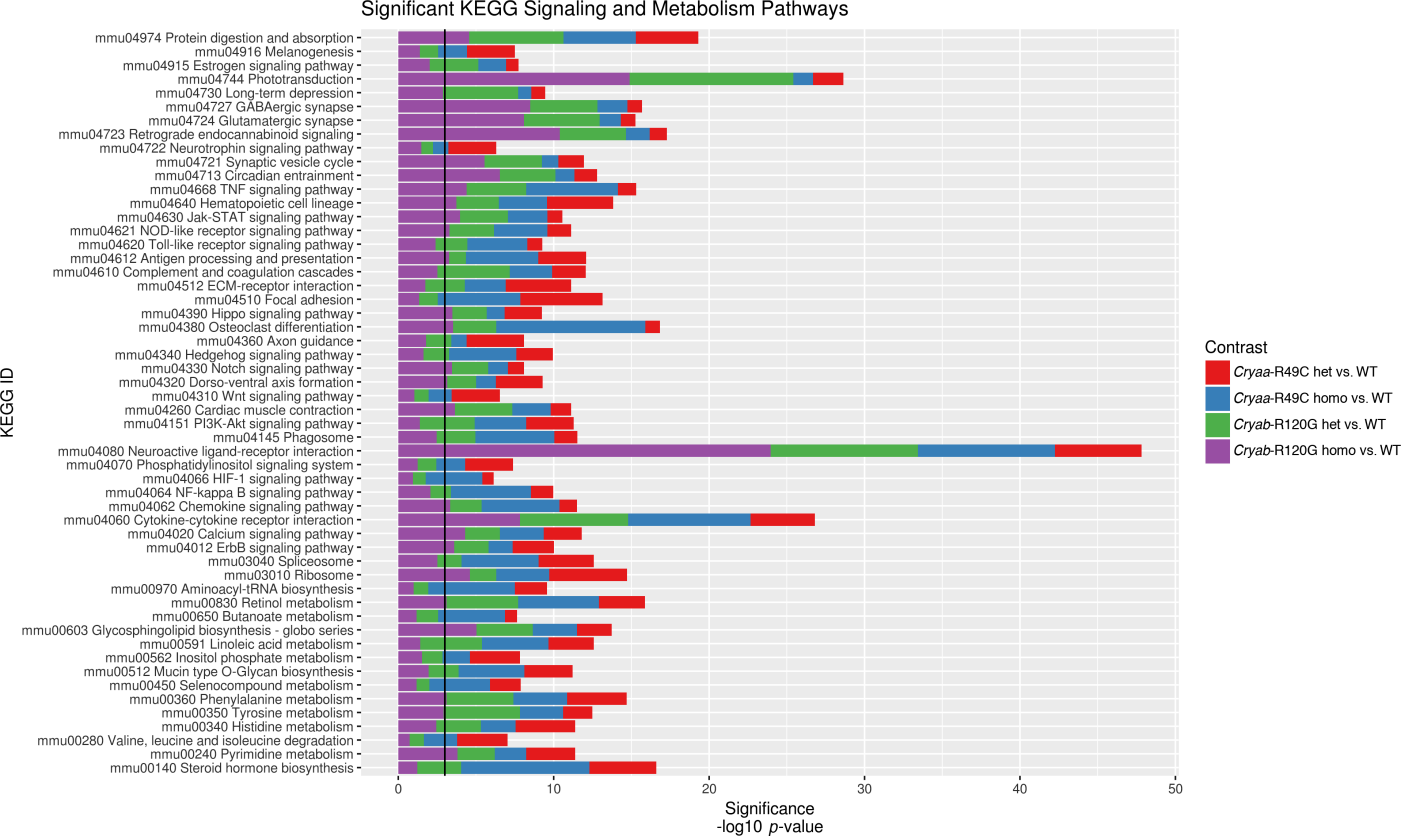
Barplot of significantly perturbed KEGG signaling and metabolism pathways across all four mutant genotypes. The statistical significance shown on the x-axis has been–log 10 scaled. Each pathway has 4 stacked bars showing the significance (not the magnitude) of each pathway for each genotype. The *p*-value cutoff of 0.05 is indicated by the vertical black line, to differentiate pathways significant for one genotype versus the others.

The changes in gene expression in lenses from the *Cryaa*-R49C and *Cryab*-R120G mutated mice were examined using KEGG analysis (Fig 5). This analysis demonstrated that these mutations affect genes in a wide range of metabolic pathways (S6 and S7 Figs). The top metabolic pathways identified in lenses with the *Cryaa*-R49C mutation were those for steroid hormone biosynthesis, selenocompound metabolism, mucin-type O-glycan biosynthesis, and linoleic acid metabolism. In the *Cryab*-R120G mutant lenses, the affected metabolic pathways were those for pyrimidine metabolism, aminoacyl t-RNA biosynthesis, protein processing in the endoplasmic reticulum, and cardiac muscle contraction pathways. In addition, pathways for ribosomal RNA metabolism, spliceosome and cytokine-cytokine receptor interaction were among the metabolic pathways that were affected in lenses with either *Cryaa*-R49C or *Cryab*-R120G mutation. The involvement of the steroid biosynthesis pathway in lenses with the *Cryaa*-R49C mutation is interesting because recent findings indicate that certain topically applied cholesterol derivatives suppress lens opacities in mice with the *Cryab*-R120G or *Cryaa*-R49C mutations [32].

## Discussion

The arginine 49 to cysteine mutation in αA-crystallin (*Cryaa*-R49C) and arginine 120 to glycine mutation in αB-crystallin (*Cryab*-R120G) are associated with autosomal dominant human cataracts [7, 8]. We developed knock-in mouse models to investigate the mechanism of hereditary cataract formation and demonstrated that the knock-in mice develop cataracts, and undergo changes in abundance of specific lens protein at an early postnatal age [9, 10]. In this study, we sought to determine whether apparent 2-3-fold increases in histone expression we observed by proteomic analysis occurred at the transcriptional level in *Cryaa*-R49C and *Cryab*-R120G knock-in mutant lenses obtained from young mice at an early postnatal age [20]. Our findings indicate an increase in the transcript levels of specific histones, suggesting that mutant αA-crystallin protein affects the transcript levels of certain histone genes. We investigated lenses from 2-day-old *Cryaa*-R49C mice because an increase in histone levels was previously observed via proteomics analysis at this age [20]. The heat maps showed an increase in numerous histone transcripts in the *Cryaa*-R49C knock-in mutant mouse lenses. These findings suggest that the previously reported increase in histones observed by proteomics analysis occurred at the transcript level. Interestingly, transcripts for the core histones H2A, H2B, and H4, which are regulated during the cell cycle, were significantly enhanced in the *Cryaa*-R49C homo lenses, suggesting that the increased expression of histones may be a beneficial response induced in the absence of any wild type αA-crystallin protein. In addition, the increase in transcripts for histone H1, which binds to the linker DNA between the nucleosomes and is thought to be important for the higher order structure of chromatin [33] in *Cryaa*-R49C homo lenses suggest a role for the αA-crystallin in the higher order structure of chromatin. Moreover, the observed increases in histone transcripts in *Cryaa*-R49C homo lenses, as well as the increase in histone *H2afx* transcripts in *Cryab*-R120G homo lenses suggest that histone expression is selectively regulated in the absence of any wild type αA- or αB-crystallin proteins in mouse lenses. Whether such an increase in histone expression occurs during aging or cataract development in the human lens remains to be investigated.

These findings are particularly important since small heat shock proteins have been shown to be involved in regulating transcription [34] and αA-crystallin has been reported to interact with a sequence in γ-crystallin gene promoters and helps regulate their transcription [35, 36]. The increase in H2A.X in the *Cryab*-R120G homo lenses is also consistent with the role of α-crystallin in the lens nucleus, since *Cryab* null mouse lens epithelial cells had increased genomic instability and an increased proportion of polyploid cells in vitro, and *Cryaa* null mouse lens epithelial cells had reduced proliferation in vivo [37, 38]. In addition, the supply of histones has been shown to regulate the timing of the S-phase and progression of the cell cycle [39]. Furthermore, an increase in expression of α-crystallin occurs as lens epithelial cells enter the cell cycle, and cell cycle entry into the S phase is delayed in lens epithelial cells derived from *Cryaa*;*Cryab* gene knockout (DKO) null mice [40, 41]. Studies on lenses show that lens-specific chromatin domain borders are marked by histone H3 acetylation [42]. In addition, the histone chaperone NASP has been found to fine-tune a reservoir of soluble histone H3-H4 [43]. Thus, one function of α-crystallin may be as a tunable reservoir of histones, although additional studies are required to investigate the possibility that α-crystallin modulates the soluble histone pool in the lens. Although α-crystallin is predominantly a cytoplasmic protein, it has been detected in the nuclei of rat lens epithelial explants, and in other organelles such as the Golgi and mitochondria, and the *Cryaa*-R49C mutant protein is expressed mainly in the nucleus of transfected lens epithelial cells, where it may bind histones [7, 44]. Intriguingly, recent work suggests that aging is associated with a significant loss of histone proteins, and increasing the histone supply supports lifespan extension in yeast [45]. Thus, the physiological relevance of increased histone transcripts in the lenses of mice during early cataract development may be a beneficial response. Further studies using co-immunolocalization studies and PLA are underway, as these results are necessary for functional interpretation of the transcript data.

RNA-seq has previously been used to probe the transcriptional changes that occur during lens differentiation, development and cataract [46–49]. Our studies revealed a significant level of lncRNAs in the postnatal mouse lenses in agreement with previous studies on embryonic mouse lenses. Importantly, our RNA-seq data indicate that transcriptional changes in the lenses of *Cryaa*-R49C and *Cryab*-R120G mutant mice occurred at an early age, despite the relatively small increase in the molecular weight of α-crystallin at these ages (S6 Table). In addition to histones, RNA-seq analysis also identified many genes that exhibited altered abundance in the mutant lenses. For example, in *Cryaa*-R49C-het mouse lenses, decreased levels of *Clic5*, an intracellular chloride channel that stabilizes membrane-actin filament linkages on hair cells, are consistent with a role of α-crystallin in stabilizing actin filaments [50, 51]. Similarly, downregulation of the transcription factors *Aebp1* and *Rsad2*, which are regulators of cell differentiation, suggest that these proteins are likely associated with early changes in cataract development [52, 53]. *Ddit3*, also known as CHOP, is an ER overload response transcription factor and a proapoptotic gene, was markedly increased in *Cryaa*-R49C-homo lenses, consistent with prior reports of upregulation of the CHOP protein in adult *Cryaa*-R49C-homo lenses [54]. This finding suggests that some of the observed changes in gene expression in *Cryaa*-R49C-homo lenses may be related to the strong upregulation of *Ddit3*. Also, the expression of *Trib3*, an unfolded protein response (UPR) gene, was increased 509-fold in the lenses from *Cryaa*-R49C-homo mice. *Trib3* is also enhanced during cataract development in major intrinsic protein of lens fiber (MIP)-mutant mice [55]. The expression of *Chac1*, a cation transport regulator that is also involved in ER stress, was increased in the *Cryaa*-R49C-homo mouse lenses, as in the MIP-mutant lenses [56]. In contrast, the decreased expression of *Tcp11*, a determinant of sperm morphology, as well as the decreased expression of *Stx11*, a gene involved in tumor suppression, suggest roles for these factors in the small eye phenotype of the *Cryaa*-R49C-homo lenses [57, 58]. In addition, the decreased expression of *Hspb1*, which encodes Hsp27 and is related to actin cytoskeletal dynamics, is consistent with the protective effect of α-crystallin on actin [20].

The expression of *Slc6a13*, which encodes a transporter that regulates the entry of solutes including amino acids into cells [59], was increased 5.3-fold and 5.7-fold in *Cryab*-R120G-het and *Cryab*-R120G-homo mouse lenses, respectively, compared with expression in WT lenses. The 24-fold increase in *Cryab*-R120G-het mouse lenses for *Tfap2b*, which encodes a transcription factor that plays a role in development and survival, suggests that this mutation affects lens development and morphology, a phenotype that was reported previously [10, 60]. Our findings also demonstrate a 48-fold increase in *Vax203* gene expression, and this gene is linked with transcription factors that are known to play a role in eye development and function [61]. Furthermore, the observed 4-fold increase in *Ttr*, which can form amyloids, is involved in cardiomyopathy and is a carrier of retinol, suggesting a role for this protein in cataract development in *Cryab*-R120G-het mouse lenses [62, 63]. Finally, the increase in *Hba-a2* in the *Cryab*-R120G-het lenses suggests an effect of oxidative stress in the *Cryab*-R120G-mutant cataracts, since hemoglobin monomers have been found in vertebrate tissues other than blood, where they play a role in modulating the redox balance [64–67]. At present we have not investigated the role of α-crystallin chaperone function on gene expression changes. Studies reported in the literature suggest that the *Cryaa*-R49C and *Cryab*-R120G mutations cause structural changes in α-crystallin, and these changes may reduce chaperone function [68, 69]. Importantly, the gene expression changes observed in this study do not necessarily indicate corresponding changes in the protein levels. While it is interesting and perhaps informative to speculate on the relationship between the alterations in transcripts and functional relevance, further studies will be necessary to validate the altered transcript levels and to determine whether these changes are related to actual protein level changes as well as to specific lens functions.

In summary, we identified genes that were altered in early cataract development due to mutations in *Cryaa* and *Cryab* genes. Some of the identified genes are known to be involved in development, transport, apoptosis, and UPR. We also demonstrated an increase in the expression of several histone genes. Given the importance of histones in cell growth and genomic stability, our data provide further insight into the mechanism of hereditary cataract formation. Further work is necessary to fully understand the relationship between α-crystallin mutations, histone integrity, and cataract development. Additionally, the identification of pathways affected by α-crystallin mutations may accelerate the development of non-surgical therapeutic approaches to treat cataract.

## Supporting information

S1 Fig Transcript biotype distribution of *Cryaa*-R49C and *Cryab*-R120G mouse lenses used in this study. (DOCX)

S2 Fig Gene biotype analysis of *Cryaa*-R49C and *Cryab*-R120G mouse lenses used in this study. (DOCX)

S3 Fig End bias plots for the RNA-seq analysis. (DOCX)

S4 Fig Pearson correlation plots for the RNA-seq analysis. (DOCX)

S5 Fig MDS plots. Samples that are less similar are separated by larger distances, whereas samples that are most similar occupy closer positions in the plots. At the gene level, the *Cryaa*-R49C-homo samples were clustered together on the x- and y-axes and were separated from the spots corresponding to WT and *Cryaa*-R49C-het samples. In contrast, at the transcript level, two of the *Cryaa*-R49C-homo samples were separated from the third, demonstrating the biological coefficient of variation between the three replicates. *Cryab*-R120G mouse lenses were separated from the *Cryaa*-R49C mouse lenses along the second dimension (y-axis). The *Cryab* samples exhibited increased variance between the three genotypes. That is, each of the three WT, heterozygous, and homozygous *Cryab*-R120G mutants varied significantly.
(DOCX)

S6 Fig Output of GAGE and Pathview KEGG analysis for 2-day-old *Cryaa*-R49C mouse lenses. Blue boxes represent down-regulated genes or complexes of genes or their proteins and yellow boxes are up-regulated genes or complexes of genes or proteins. Data are plotted on a compressed mean log 2 fold-change scale. (A) *Cryaa*-R49C-het vs. WT lenses; (B) *Cryaa*-R49C-homo vs. WT lenses.
(PDF)

S7 Fig Output of GAGE and Pathview KEGG analysis for 14-day-old *Cryab*-R120G mouse lenses where blue boxes represent down-regulated genes or complexes of genes or their proteins and yellow boxes are up-regulated genes or complexes of genes or their proteins. Data are plotted on a compressed mean log 2 fold-change scale. (A) *Cryab*-R120G-het vs. WT lenses; (B) *Cryab*-R120G-homo vs. WT lenses.
(PDF)

S1 Table The number of lenses used in each sample. (XLSX)

S2 Table A list of qPCR primers (life technologies) used in this study. (XLSX)

S3 Table STAR summary. (XLS)

S4 Table List of altered genes in *Cryaa*-R49C-homo mouse lenses with FDR<0.05. (XLSX)

S5 Table List of altered genes in *Cryab*-R120G-homo mouse lenses with FDR<0.05. (XLSX)

S6 Table Molecular weight (MW) of α-crystallin from *Cryaa*-R49C and *Cryab*-R120G knock-in mouse lenses. (DOCX)

## Abbreviations

BSA: bovine serum albumin
FDR: false discovery rate
GAGE: generally applicable gene-set enrichment
GPC: gel permeation chromatography
GEO: Gene Expression Omnibus
het: heterozygous
homo: homozygous
KEGG: Kyoto Encyclopedia of Genes and Genomes
lncRNAs: long non-coding RNAs
MA plots: log ratio and mean average plots
MDS plots: multi-dimensional scaling plots
NCBI: National Center for Biotechnology Information
PBS: phosphate-buffered saline
qPCR: real-time quantitative PCR
STAR: Spliced Transcripts Alignment to Reference
TMM: trended mean of M values
UPR: unfolded protein response
WT: wild-type
