# Supplementary material for: Probing the changes in gene expression due to α-crystallin mutations in mouse models of hereditary human cataract
Source: PLoS One. 2018 Jan 16;13(1):e0190817. doi: 10.1371/journal.pone.0190817 (PMC5770019; doi:10.1371/journal.pone.0190817)
Supplement: S1 Fig — (DOCX) [file pone.0190817.s001.docx]

**S1 Fig**


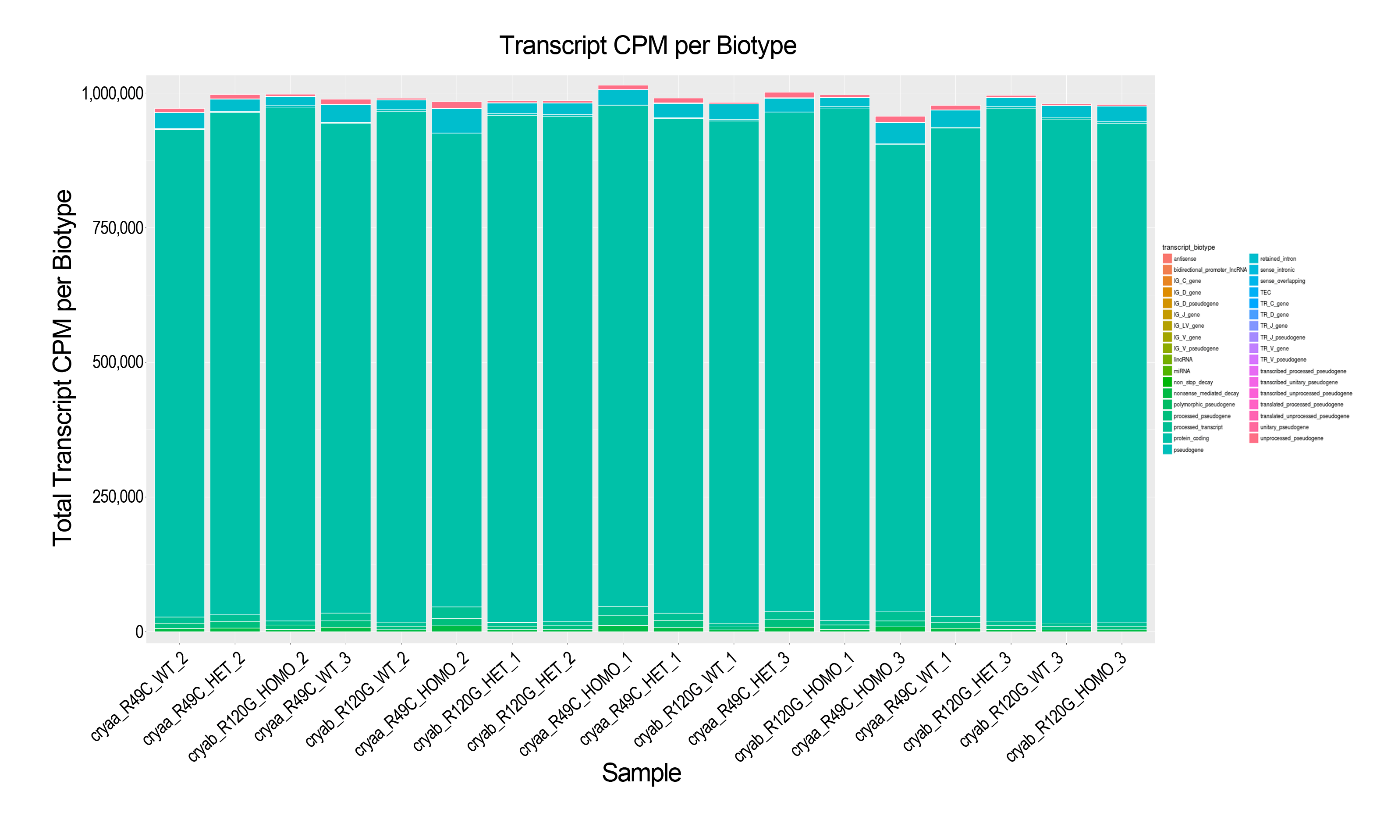


**S1 Fig:** Transcript biotype distribution of *cryaa*-R49C and *cryab*-R120G mouse lenses used in this study.
