# Supplementary figures and images for: Probing the changes in gene expression due to α-crystallin mutations in mouse models of hereditary human cataract
Source: PLoS One. 2018 Jan 16;13(1):e0190817. doi: 10.1371/journal.pone.0190817 (PMC5770019; doi:10.1371/journal.pone.0190817)

**S2 Fig**


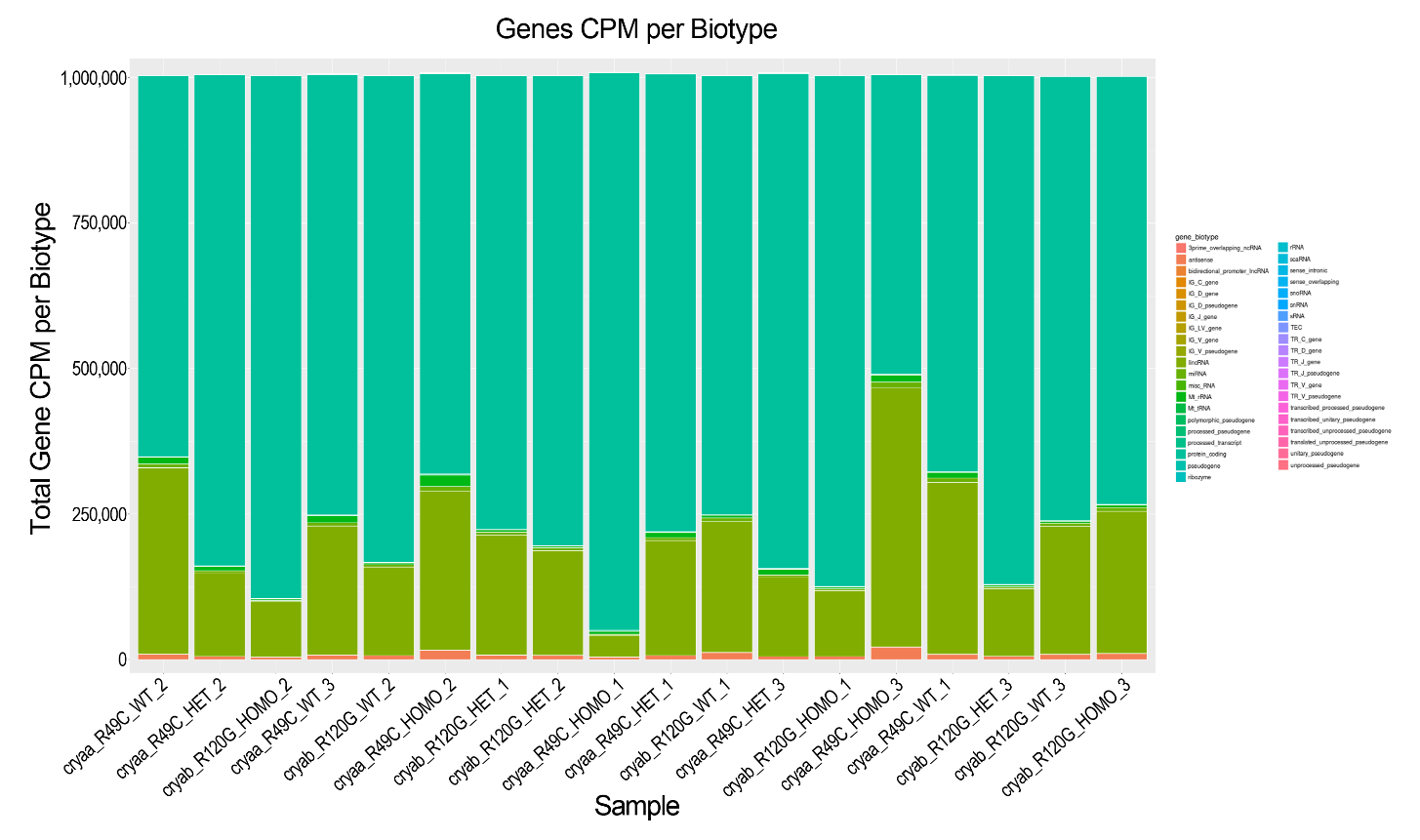


**S2 Fig**: Gene biotype analysis of *cryaa*-R49C and *cryab*-R120G mouse lenses used in this study.

Supplement: S2 Fig — (DOCX) [file pone.0190817.s002.docx]

**S3 Fig**


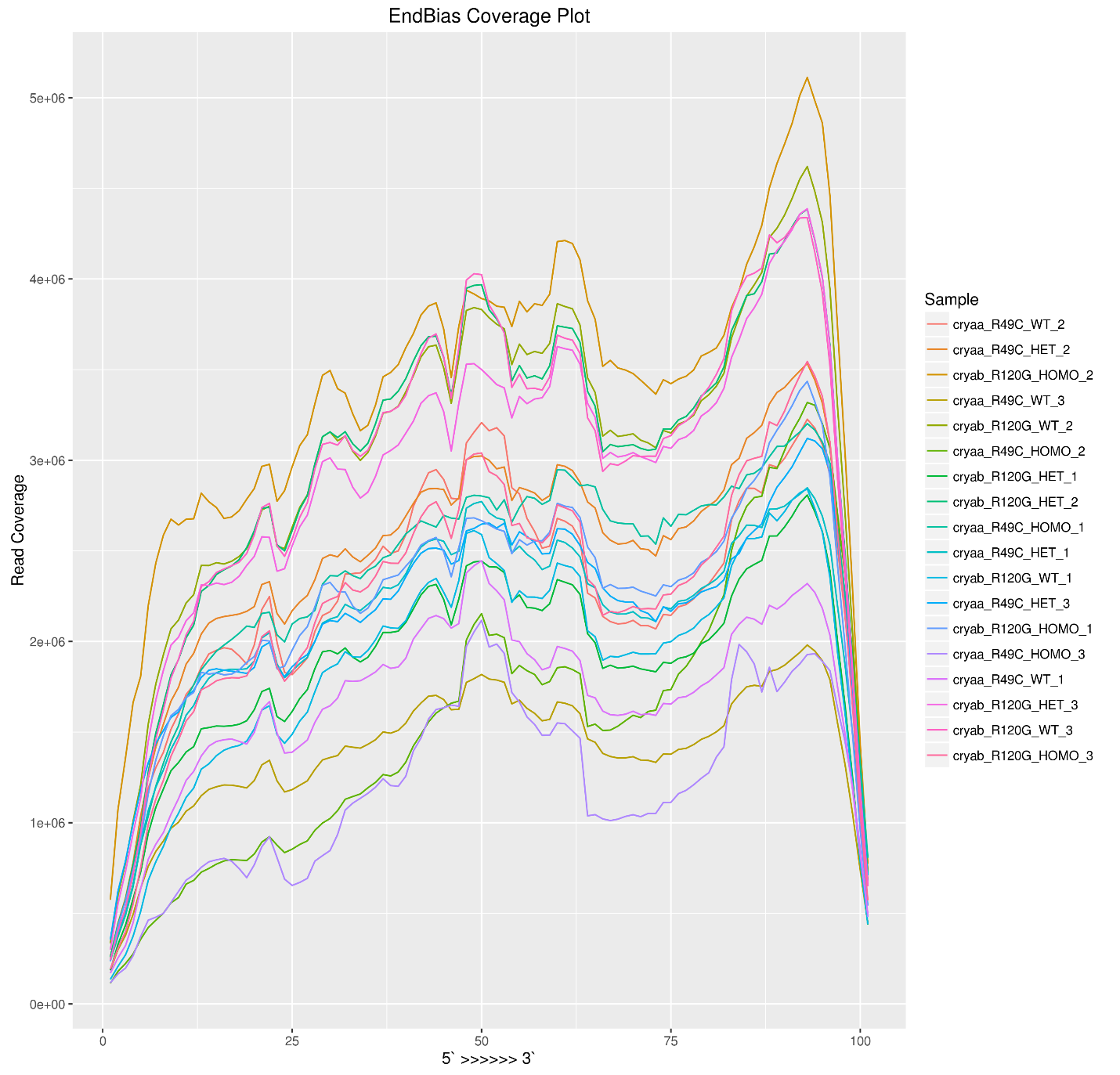


**S3 Fig:** End bias plots for the RNA-seq analysis.

Supplement: S3 Fig — (DOCX) [file pone.0190817.s003.docx]

**S4 Fig**


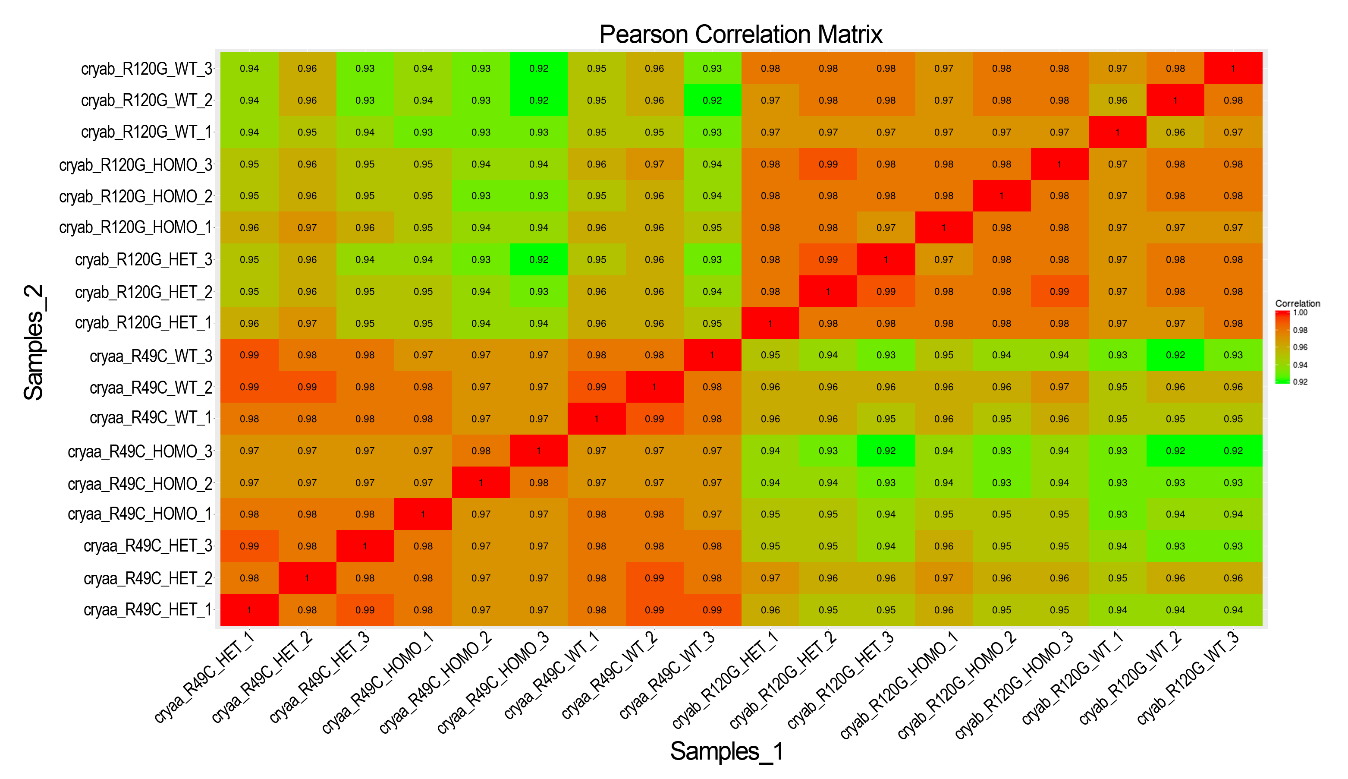


**S4 Fig:** Pearson correlation plots for the RNA-seq analysis.

Supplement: S4 Fig — (DOCX) [file pone.0190817.s004.docx]
