# Supplementary material for: Probing the changes in gene expression due to α-crystallin mutations in mouse models of hereditary human cataract
Source: PLoS One. 2018 Jan 16;13(1):e0190817. doi: 10.1371/journal.pone.0190817 (PMC5770019; doi:10.1371/journal.pone.0190817)
Supplement: S5 Fig — Samples that are less similar are separated by larger distances, whereas samples that are most similar occupy closer positions in the plots. At the gene level, the Cryaa-R49C-homo samples were clustered together on the x- and y-axes and were separated from the spots corresponding to WT and Cryaa-R49C-het samples. In contrast, at the transcript level, two of the Cryaa-R49C-homo samples were separated from the third, demonstrating the biological coefficient of variation between the three replicates. Cryab-R120G mouse lenses were separated from the Cryaa-R49C mouse lenses along the second dimension (y-axis). The Cryab samples exhibited increased variance between the three genotypes. That is, each of the three WT, heterozygous, and homozygous Cryab-R120G mutants varied significantly. (DOCX) [file pone.0190817.s005.docx]

**S5 FIG**


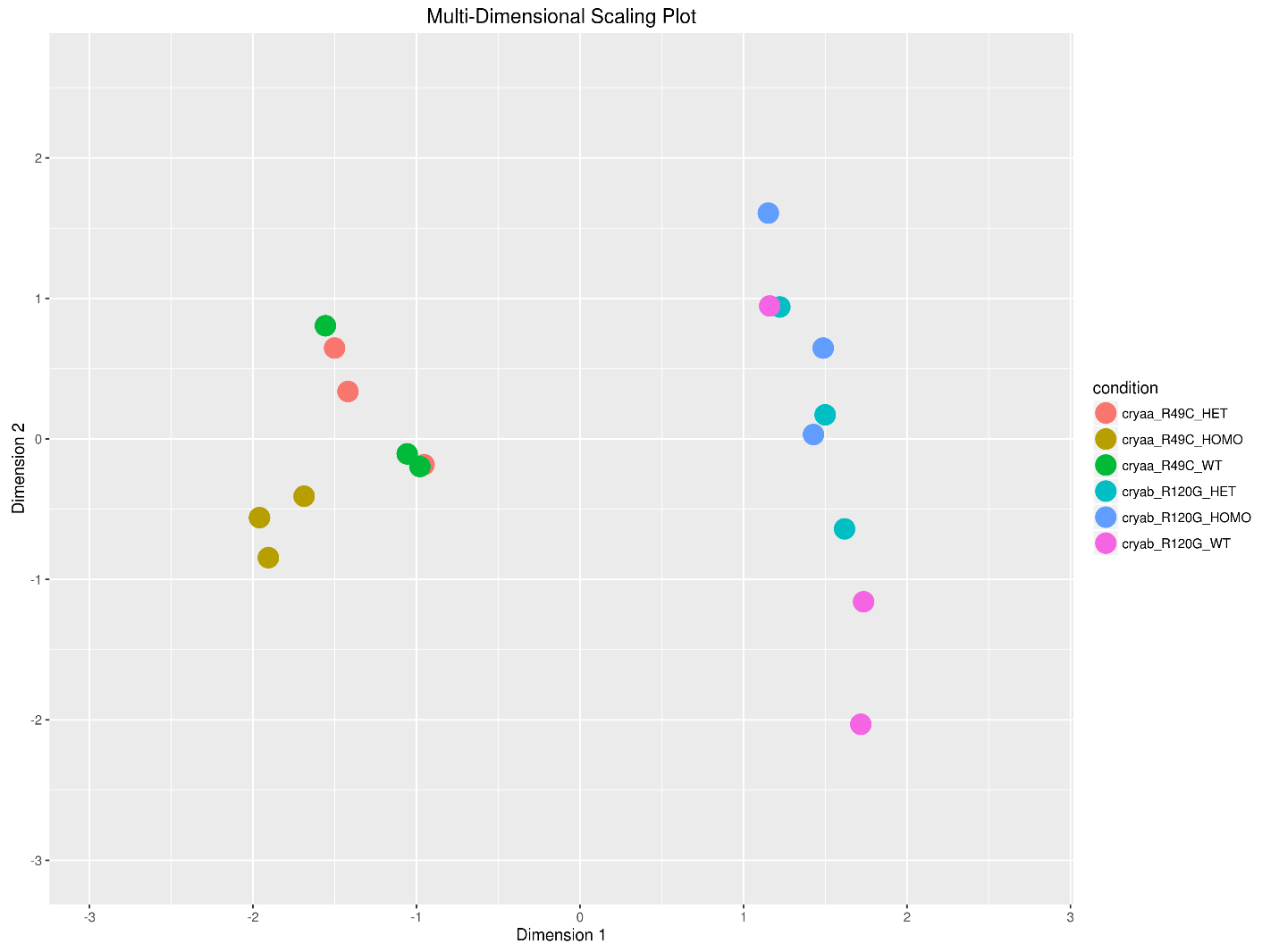


**S5 Fig:** MDS plots. Samples that are less similar are separated by larger distances, whereas samples that are most similar occupy closer positions in the plots. At the gene level, the *cryaa*-R49C-homo samples were clustered together on the x- and y-axes and were separated from the spots corresponding to WT and *cryaa*-R49C-het samples. In contrast, at the transcript level, two of the *cryaa*-R49C-homo samples were separated from the third, demonstrating the biological coefficient of variation between the three replicates. *Cryab*-R120G mouse lenses were separated from the *cryaa*-R49C mouse lenses along the second dimension (y-axis). The *cryab* samples exhibited increased variance between the three genotypes. That is, each of the three WT, heterozygous, and homozygous *cryab*-R120G mutants varied significantly.
