# Supplementary material for: Probing the changes in gene expression due to α-crystallin mutations in mouse models of hereditary human cataract
Source: PLoS One. 2018 Jan 16;13(1):e0190817. doi: 10.1371/journal.pone.0190817 (PMC5770019; doi:10.1371/journal.pone.0190817)
Supplement: S6 Fig — Blue boxes represent down-regulated genes or complexes of genes or their proteins and yellow boxes are up-regulated genes or complexes of genes or proteins. Data are plotted on a compressed mean log 2 fold-change scale. (A) Cryaa-R49C-het vs. WT lenses; (B) Cryaa-R49C-homo vs. WT lenses. (PDF) [file pone.0190817.s006.pdf]

S6A Fig

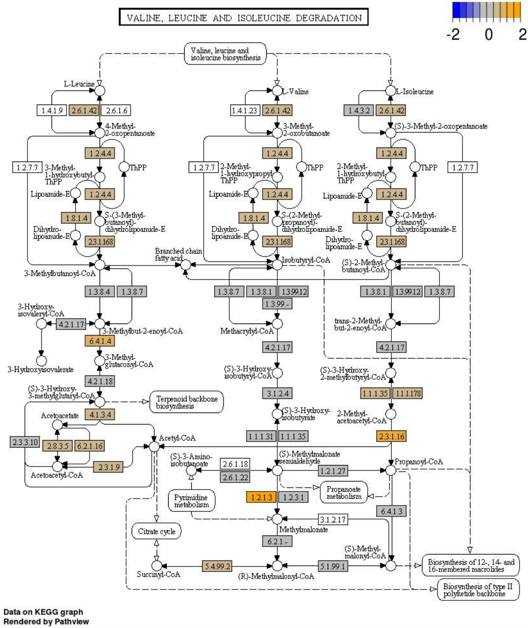
VALINE, LEUCINE AND ISOLEUCINE DEGRADATION
-2
0
2
Valine, leucine and isoleucine biosynthesis
L-Leucine
1.4.1.9
2.6.1.42
2.6.1.6
4-Methyl-2-oxopentanoate
1.2.7.7
1.2.4.4
3-Methyl-1-hydroxybutyl-ThPP
ThPP
Lipoamide-E
1.2.4.4
1.8.1.4
S-(3-Methylbutanoyl)-dihydrolipoamide-E
Dihydro-lipoamide-E
2.3.1.168
3-Methylbutanoyl-CoA
Branched chain fatty acid
1.3.8.4
1.3.8.7
3-Hydroxy-isovaleryl-CoA
4.2.1.17
3-Methylbut-2-enoyl-CoA
6.4.1.4
3-Hydroxyisovalerate
3-Methyl-glutaconyl-CoA
4.2.1.18
(S)-3-Hydroxy-3-methylglutaryl-CoA
Terpenoid backbone biosynthesis
Acetoacetate
4.1.3.4
2.3.3.10
2.8.3.5
6.2.1.16
Acetyl-CoA
Acetoacetyl-CoA
2.3.1.9
L-Valine
1.4.1.23
2.6.1.42
3-Methyl-2-oxobutanoate
1.2.7.7
1.2.4.4
2-Methyl-1-hydroxypropyl-ThPP
ThPP
Lipoamide-E
1.2.4.4
1.8.1.4
S-(2-Methylpropanoyl)-dihydrolipoamide-E
Dihydro-lipoamide-E
2.3.1.168
Isobutyryl-CoA
1.3.8.7
1.3.8.1
1.3.99.12
1.3.99.-
Methacrylyl-CoA
4.2.1.17
(S)-3-Hydroxy-isobutyryl-CoA
3.1.2.4
(S)-3-Hydroxy-isobutyrate
1.1.1.31
1.1.1.35
(S)-3-Amino-isobutanoate
2.6.1.18
2.6.1.22
(S)-Methylmalonate semialdehyde
1.2.1.27
Pyrimidine metabolism
1.2.1.3
1.2.3.1
Propanoate metabolism
Methylmalonate
3.1.2.17
Citrate cycle
6.2.1.-
Succinyl-CoA
5.4.99.2
(R)-Methylmalonyl-CoA
5.1.99.1
L-Isoleucine
1.4.3.2
2.6.1.42
(S)-3-Methyl-2-oxopentanoate
1.2.4.4
2-Methyl-1-hydroxybutyl-ThPP
ThPP
1.2.7.7
Lipoamide-E
1.2.4.4
1.8.1.4
S-(2-Methylbutanoyl)-dihydrolipoamide-E
Dihydro-lipoamide-E
2.3.1.168
(S)-2-Methyl-butanoyl-CoA
1.3.8.1
1.3.99.12
1.3.8.7
trans-2-Methyl-but-2-enoyl-CoA
4.2.1.17
(S)-3-Hydroxy-2-methylbutyryl-CoA
1.1.1.35
1.1.1.178
2-Methyl-acetoacetyl-CoA
2.3.1.16
Propanoyl-CoA
6.4.1.3
(S)-Methyl-malonyl-CoA
Biosynthesis of 12-, 14- and 16-membered macrolides
Biosynthesis of type II polyketide backbone
Data on KEGG graph
Rendered by Pathview

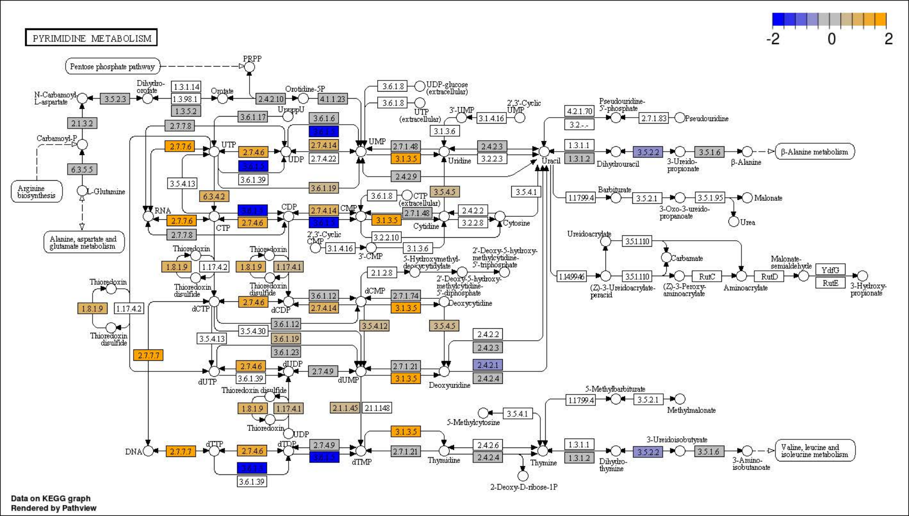
PYRIMIDINE METABOLISM
-2
0
2
Pentose phosphate pathway
PRPP
N-Carbamoyl-L-aspartate
3.5.2.3
Dihydro-orotate
1.3.1.14
1.3.98.1
1.3.5.2
Orotate
2.4.2.10
Orotidine-5P
4.1.1.23
UppppU
3.6.1.17
3.6.1.6
2.7.7.8
2.1.3.2
Carbamoyl-P
6.3.5.5
Arginine biosynthesis
L-Glutamine
Alanine, aspartate and glutamate metabolism
UTP
2.7.7.6
2.7.4.6
UDP
2.7.4.14
2.7.4.22
3.6.1.39
3.5.4.13
6.3.4.2
3.6.1.19
UMP
3.6.1.8
UDP-glucose (extracellular)
UTP (extracellular)
2.7.1.48
3.1.3.5
2.4.2.9
3.1.3.6
3'-UMP
3.1.4.16
2',3'-Cyclic UMP
Uridine
2.4.2.3
3.2.2.3
Uracil
4.2.1.70
3.2.-.-
Pseudouridine-5'-phosphate
2.7.1.83
Pseudouridine
1.3.1.1
1.3.1.2
Dihydrouracil
3.5.2.2
3-Ureido-propionate
3.5.1.6
β-Alanine
β-Alanine metabolism
RNA
2.7.7.6
2.7.7.8
CTP
2.7.4.6
CDP
2.7.4.14
CMP
3.1.3.5
3.6.1.8
CTP (extracellular)
2.7.1.48
3.5.4.5
Cytidine
2.4.2.2
3.2.2.8
Cytosine
3.5.4.1
3.2.2.10
2',3'-Cyclic CMP
3.1.4.16
3'-CMP
3.1.3.6
1.1799.4
Barbiturate
3.5.2.1
3-Oxo-3-ureido-propanoate
3.5.1.95
Malonate
Urea
Ureidoacrylate
3.5.1.110
Carbamate
1.149946
(Z)-3-Ureidoacrylate-peracid
3.5.1.110
(Z)-3-Peroxy-aminoacrylate
RutC
Aminoacrylate
RutD
Malonate-semialdehyde
YdfG
RutE
3-Hydroxy-propionate
Thioredoxin
1.8.1.9
1.17.4.2
Thioredoxin disulfide
1.17.4.1
5-Hydroxymethyl-deoxycytidine
2'-Deoxy-5-hydroxy-methylcytidine-5'-triphosphate
2.1.2.8
2'-Deoxy-5-hydroxy-methylcytidine-5'-diphosphate
dCTP
2.7.4.6
dCDP
3.6.1.12
2.7.4.14
dCMP
2.7.1.74
3.1.3.5
Deoxycytidine
3.5.4.12
3.5.4.5
3.5.4.30
3.6.1.19
3.6.1.23
3.5.4.13
2.7.7.7
2.4.2.2
2.4.2.3
2.4.2.1
2.4.2.4
dUTP
2.7.4.6
dUDP
2.7.4.9
dUMP
2.7.1.21
3.1.3.5
Deoxyuridine
3.6.1.39
Thioredoxin disulfide
1.8.1.9
1.17.4.1
Thioredoxin
UDP
2.1.1.45
2.1.1.148
5-Methylbarbiturate
1.1799.4
3.5.2.1
Methylmalonate
5-Methylcytosine
3.5.4.1
DNA
2.7.7.7
dTTP
2.7.4.6
dTDP
2.7.4.9
dTMP
3.1.3.5
2.7.1.21
Thymidine
2.4.2.6
2.4.2.4
Thymine
1.3.1.1
1.3.1.2
Dihydro-thymine
3-Ureidoisobutyrate
3.5.2.2
3.5.1.6
3-Amino-isobutanoate
Valine, leucine and isoleucine metabolism
2-Deoxy-D-ribose-1P
3.6.1.39
Data on KEGG graph
Rendered by Pathview

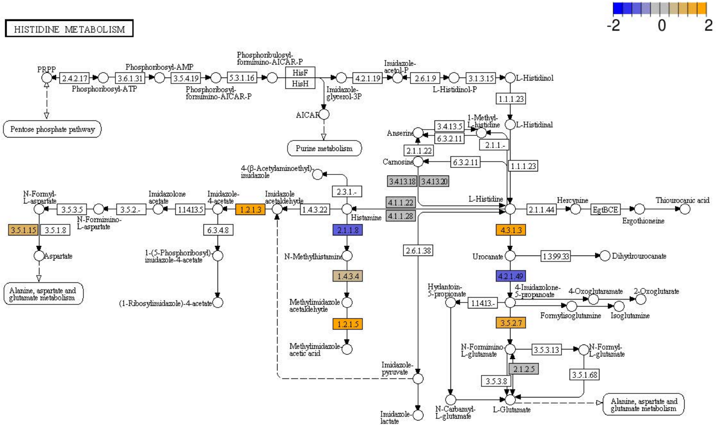


Data on KEGG graph
Rendered by Pathview

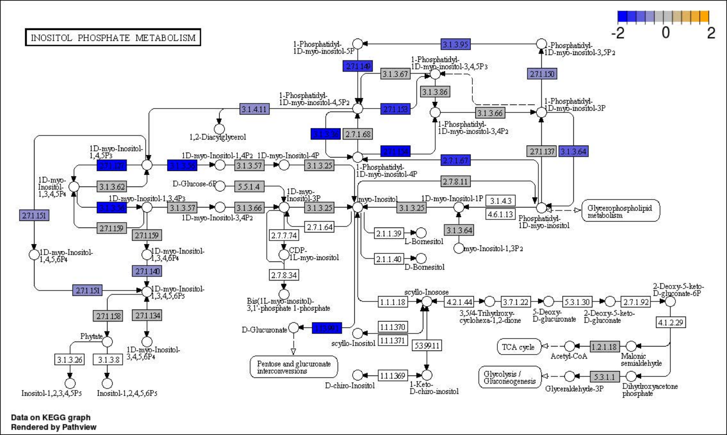

INOSITOL PHOSPHATE METABOLISM
-2
0
2
1-Phosphatidyl-1D-myo-inositol-5P
3.1.3.95
1-Phosphatidyl-1D-myo-inositol-3,5P2
2.7.1.149
3.1.3.67
1-Phosphatidyl-1D-myo-inositol-3,4,5P3
2.7.1.150
3.1.3.86
1-Phosphatidyl-1D-myo-inositol-4,5P2
2.7.1.153
3.1.3.66
1-Phosphatidyl-1D-myo-inositol-3P
3.1.4.11
1,2-Diacylglycerol
1-Phosphatidyl-1D-myo-inositol-3,4P2
3.1.3.36
2.7.1.68
2.7.1.154
2.7.1.137
3.1.3.64
2.7.1.67
1-Phosphatidyl-1D-myo-inositol-4P
1D-myo-Inositol-1,4,5P3
2.7.1.127
3.1.3.56
1D-myo-Inositol-1,4P2
3.1.3.57
1D-myo-Inositol-4P
3.1.3.25
1D-myo-Inositol-1,3,4,5P4
3.1.3.62
D-Glucose-6P
5.5.1.4
1D-myo-Inositol-3P
2.7.8.11
1D-myo-Inositol-1,3,4P3
3.1.3.56
3.1.3.57
3.1.3.66
3.1.3.25
myo-Inositol
3.1.3.25
1D-myo-Inositol-1P
3.1.4.3
4.6.1.13
Phosphatidyl-1D-myo-inositol
Glycerophospholipid metabolism
2.7.1.151
1D-myo-Inositol-3,4P2
2.7.1.159
2.7.1.159
2.7.1.64
2.7.7.74
2.1.1.39
L-Bornesitol
3.1.3.64
myo-Inositol-1,3P2
1D-myo-Inositol-1,4,5,6P4
1D-myo-Inositol-1,3,4,6P4
2.7.1.140
CDP-1L-myo-inositol
2.1.1.40
D-Bornesitol
2.7.8.34
2.7.1.151
1D-myo-Inositol-1,3,4,5,6P5
scyllo-Inosose
1.1.1.18
4.2.1.44
3,5/4-Trihydroxy-cyclohexa-1,2-dione
3.7.1.22
5-Deoxy-D-glucuronate
5.3.1.30
2-Deoxy-5-keto-D-gluconate
2.7.1.92
2-Deoxy-5-keto-D-gluconate-6P
2.7.1.158
2.7.1.134
Bis(1L-myo-inositol)-3,1'-phosphate 1-phosphate
4.1.2.29
D-Glucuronate
1.13.99.1
1.1.1.370
1.1.1.371
scyllo-Inositol
Phytate
1D-myo-Inositol-3,4,5,6P4
5.3.99.11
TCA cycle
Acetyl-CoA
1.2.1.18
Malonic semialdehyde
3.1.3.26
3.1.3.8
Pentose and glucuronate interconversions
1.1.1.369
D-chiro-Inositol
1-Keto-D-chiro-inositol
Glycolysis / Gluconeogenesis
Glyceraldehyde-3P
5.3.1.1
Dihydroxyacetone phosphate
Inositol-1,2,3,4,5P5
Inositol-1,2,4,5,6P5
Data on KEGG graph
Rendered by Pathview

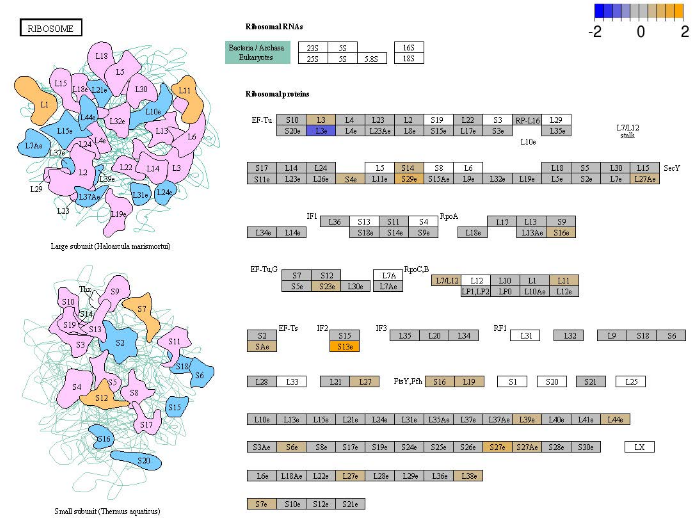


Data on KEGG graph
Rendered by Pathview

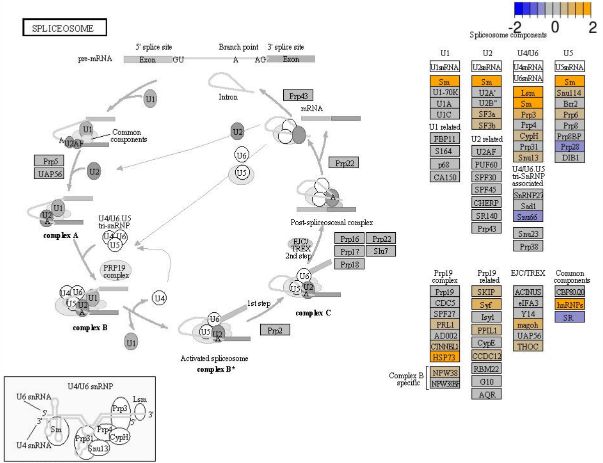


Data on KEGG graph
Rendered by Pathview

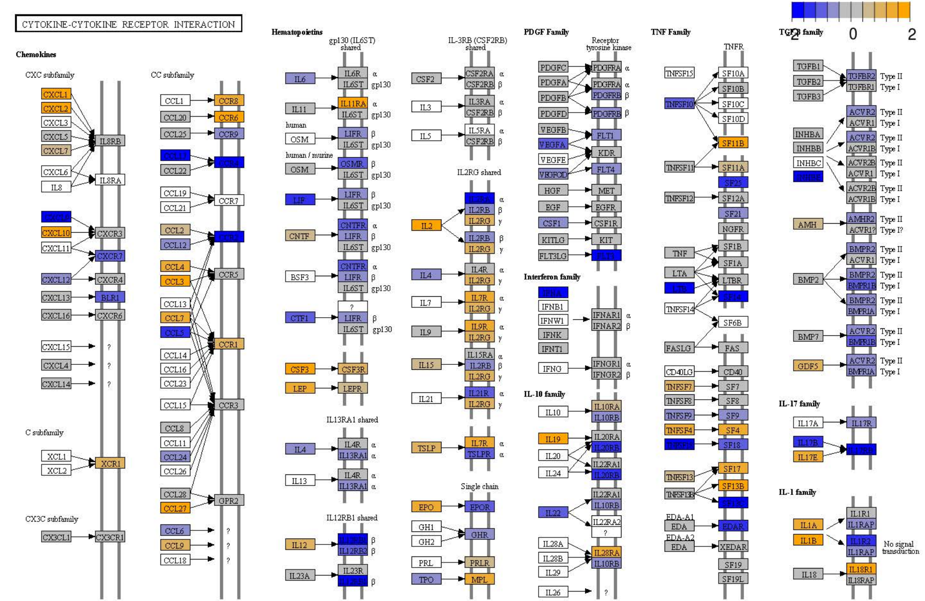


Data on KEGG graph
Rendered by Pathview

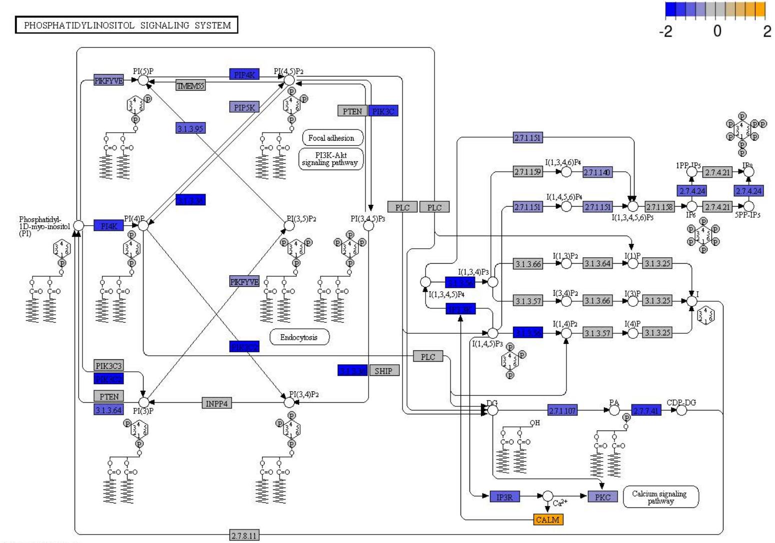


Data on KEGG graph
Rendered by Pathview

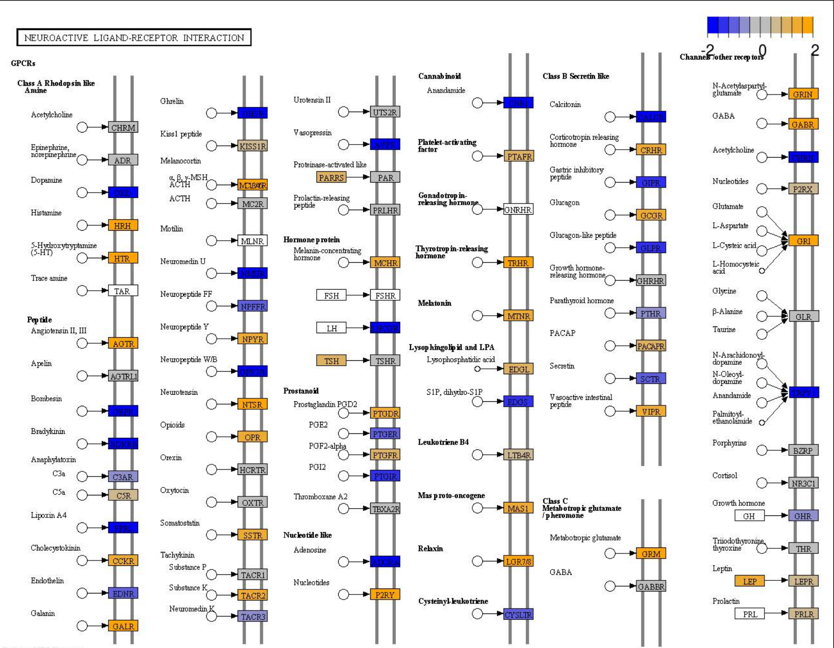


Data on KEGG graph
Rendered by Pathview

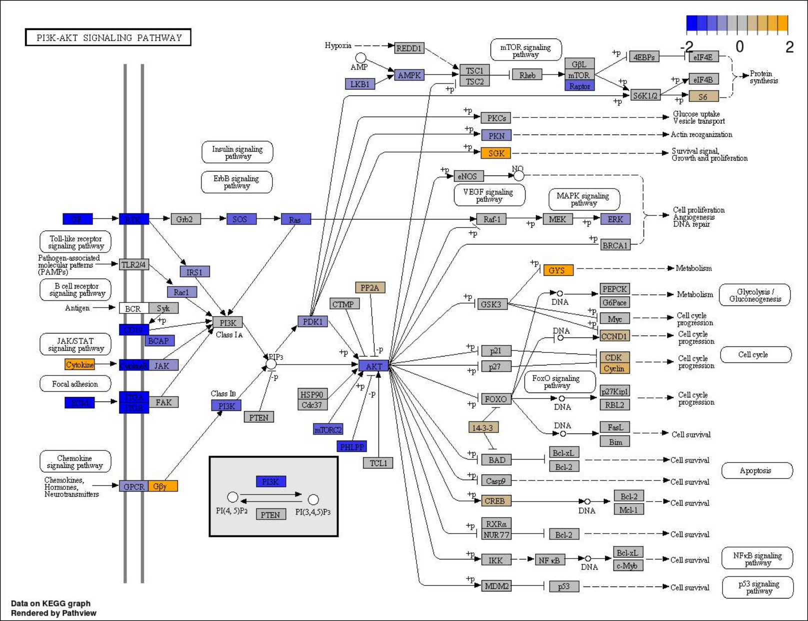
PI3K-AKT SIGNALING PATHWAY
-2
0
2
Hypoxia
REDD1
AMP
AMPK
LKB1
TSC1
TSC2
mTOR signaling pathway
Rheb
GβL
mTOR
Raptor
4EBPs
eIF4E
eIF4B
S6K1/2
S6
Protein synthesis
PKCs
Glucose uptake
Vesicle transport
PKN
Actin reorganization
SGK
Survival signal, Growth and proliferation
Insulin signaling pathway
ErbB signaling pathway
eNOS
NO
VEGF signaling pathway
MAPK signaling pathway
GF
RTK
Grb2
SOS
Ras
Raf-1
MEK
ERK
Cell proliferation
Angiogenesis
DNA repair
BRCA1
Toll-like receptor signaling pathway
Pathogen-associated molecular patterns (PAMPs)
TLR2/4
IRS1
Rac1
B cell receptor signaling pathway
Antigen
BCR
Syk
PI3K
Class IA
CD19
BCAP
JAK/STAT signaling pathway
Cytokine
CytokineR
JAK
Focal adhesion
ECM
ITGA
ITGB
FAK
Class IB
PI3K
PIP3
PTEN
PDK1
CTMP
PP2A
AKT
HSP90
Cdc37
mTORC2
PHLPP
TCL1
GYS
Metabolism
GSK3
DNA
PEPCK
G6Pace
Metabolism
Glycolysis / Gluconeogenesis
Myc
Cell cycle progression
CCND1
Cell cycle progression
p21
p27
CDK
Cyclin
Cell cycle progression
Cell cycle
FoxO signaling pathway
FOXO
p27Kip1
RBL2
Cell cycle progression
14-3-3
FasL
Bim
Cell survival
Chemokine signaling pathway
Chemokines, Hormones, Neurotransmitters
GPCR
Gβγ
BAD
Bcl-xL
Bcl-2
Cell survival
Apoptosis
Casp9
Cell survival
PI3K
PI(4, 5)P2
PTEN
PI(3,4,5)P3
CREB
Bcl-2
Mcl-1
Cell survival
RXRα
NUR77
Bcl-2
Cell survival
IKK
NF κB
Bcl-xL
c-Myb
Cell survival
NFκB signaling pathway
MDM2
p53
Cell survival
p53 signaling pathway
Data on KEGG graph
Rendered by Pathview

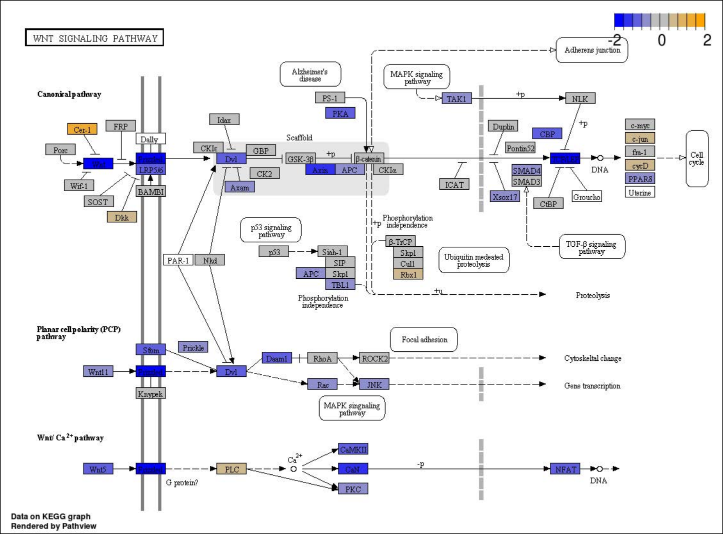

WNT SIGNALING PATHWAY
-2
0
2
Canonical pathway
Adherens junction
Alzheimer's disease
MAPK signaling pathway
PS-1
PKA
TAK1
NLK
+p
Idax
FRP
Cer-1
Dally
Porc
Wnt
Frizzled
LRP5/6
Wif-1
SOST
BAMBI
Dkk
CKIε
Dvl
GBP
Scaffold
GSK-3β
β-catenin
Axin
APC
CKIα
CK2
Axam
ICAT
Duplin
CBP
Pontin52
SMAD4
SMAD3
Xsox17
TCF/LEF
DNA
CtBP
Groucho
c-myc
c-jun
fra-1
cycD
PPARδ
Uterine
Cell cycle
p53 signaling pathway
p53
Siah-1
SIP
Skp1
TBL1
Phosphorylation independence
β-TrCP
Cul1
Rbx1
Ubiquitin mediated proteolysis
TGF-β signaling pathway
+u
Proteolysis
PAR-1
Nkd
Planar cell polarity (PCP) pathway
Sfom
Prickle
Wnt11
Knypek
Daam1
RhoA
ROCK2
Focal adhesion
Cytoskeltal change
Rac
JNK
Gene transcription
MAPK singnaling pathway
Wnt/ Ca 2+ pathway
Wnt5
G protein?
PLC
Ca 2+
CaMKII
CaN
PKC
-p
NFAT
Data on KEGG graph
Rendered by Pathview

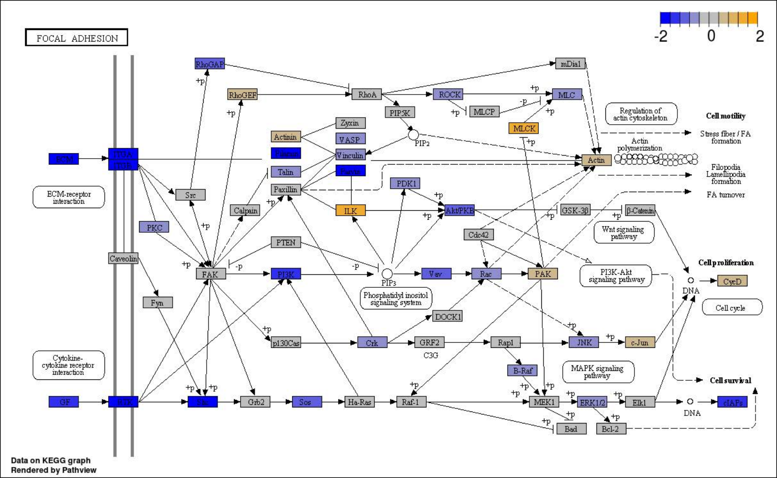

FOCAL ADHESION
-2
0
2
ECM
ITGA
ITGB
ECM-receptor interaction
Cytokine-cytokine receptor interaction
GF
RTK
Caveolin
PKC
Fyn
Src
RhoGAP
RhoGEF
RhoA
ROCK
MLCP
MLC
mDia1
PIP5K
PIP2
MLCK
Actinin
Zyxin
VASP
Filamin
Vinculin
Talin
Parvin
Paxillin
Calpain
ILK
PDK1
Akt/PKB
GSK-3β
β-Catenin
Wnt signaling pathway
Cdc42
PTEN
FAK
PI3K
PIP3
Vav
Rac
PAK
PI3K-Akt signaling pathway
Phosphatidyl inositol signaling system
DOCK1
p130Cas
Crk
GRF2
C3G
Rap1
JNK
c-Jun
B-Raf
MAPK signaling pathway
Shc
Grb2
Sos
Ha-Ras
Raf-1
MEK1
ERK1/2
Elk1
Bad
Bcl-2
DNA
cIAPs
CycD
Cell cycle
Actin
Actin polymerization
Regulation of actin cytoskeleton
Cell motility
Stress fiber / FA formation
Filopodia Lamellipodia formation
FA turnover
Cell proliferation
Cell survival
+p
-p
Data on KEGG graph
Rendered by Pathview

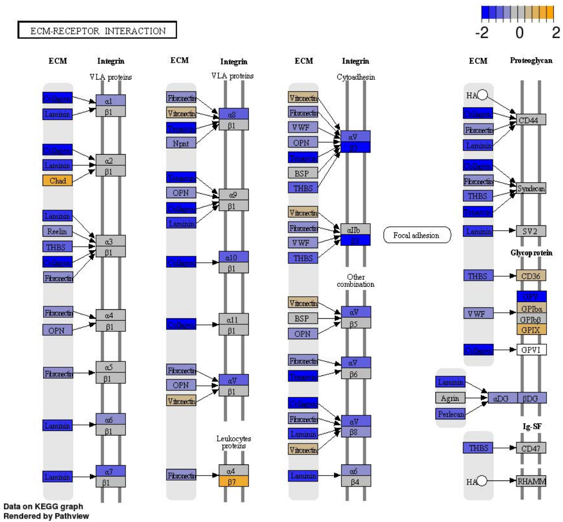

ECM-RECEPTOR INTERACTION
-2
0
2
ECM
Integrin
VLA proteins
Collagen
Laminin
α1
β1
Collagen
Laminin
Chad
α2
β1
Laminin
Reelin
THBS
Collagen
Fibronectin
α3
β1
Fibronectin
OPN
α4
β1
Fibronectin
α5
β1
Laminin
α6
β1
Laminin
α7
β1
ECM
Integrin
VLA proteins
Fibronectin
Vitronectin
Tenascin
Npnt
α8
β1
Tenascin
OPN
Collagen
Laminin
α9
β1
Collagen
α10
β1
Collagen
α11
β1
Fibronectin
OPN
Vitronectin
αV
β1
Leukocytes proteins
Fibronectin
α4
β7
ECM
Integrin
Cytoadhesin
Vitronectin
Fibronectin
VWF
OPN
Tenascin
BSP
THBS
αV
β3
Vitronectin
Fibronectin
VWF
THBS
αIIb
β3
Focal adhesion
Other combination
Vitronectin
BSP
OPN
αV
β5
Fibronectin
Tenascin
αV
β6
Collagen
Fibronectin
Laminin
Vitronectin
αV
β8
Laminin
α6
β4
ECM
Proteoglycan
HA
Collagen
Fibronectin
Laminin
CD44
Collagen
Fibronectin
THBS
Tenascin
Syndecan
Laminin
SV2
Glycoprotein
THBS
CD36
VWF
GPV
GPIbα
GPIbβ
GPIX
Collagen
GPVI
Laminin
Agrin
Perlecan
αDG
βDG
Ig-SF
THBS
CD47
HA
RHAMM
Data on KEGG graph
Rendered by Pathview

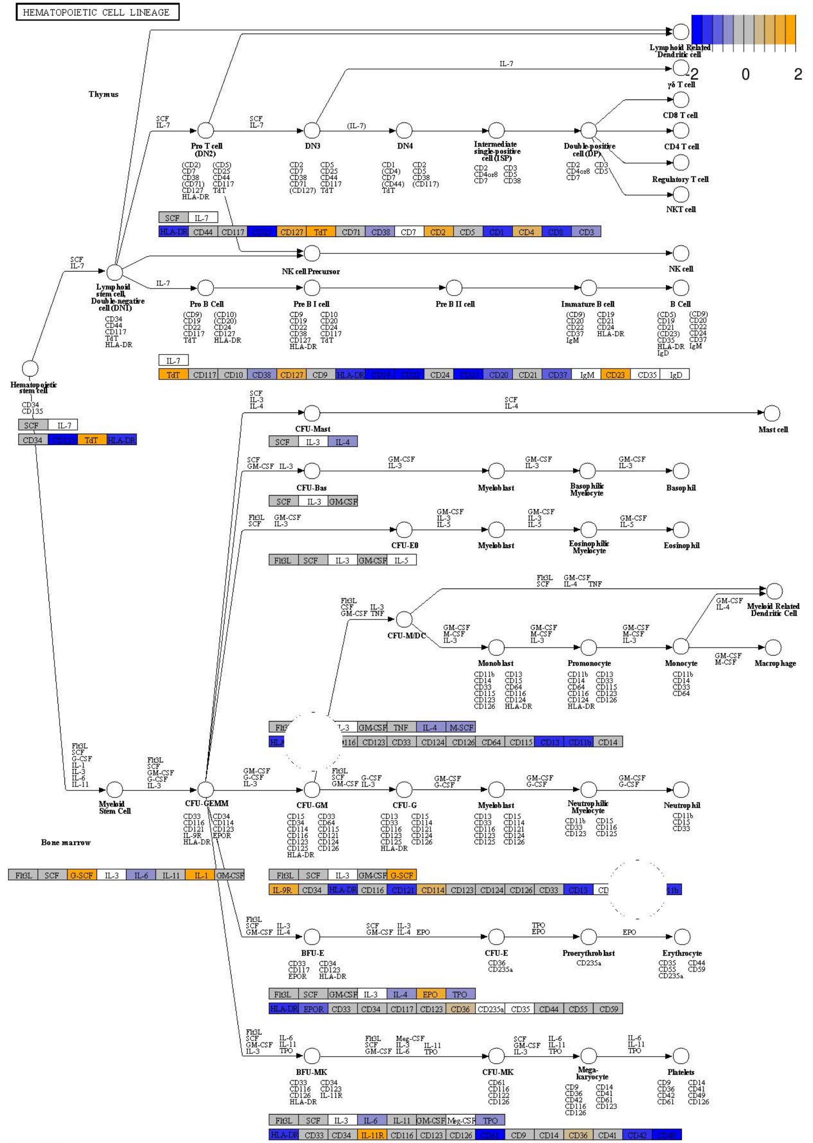


Data on KEGG graph
Rendered by Pathview

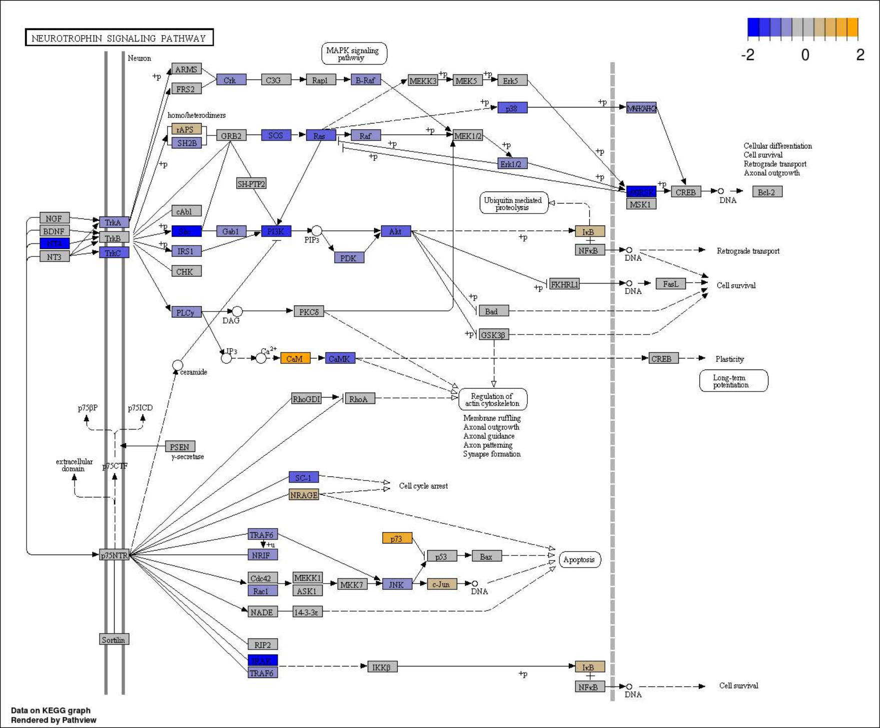
NEUROTROPHIN SIGNALING PATHWAY
-2
0
2
Neuron
MAPK signaling pathway
ARMS
FRS2
Crk
C3G
Rap1
B-Raf
MEKK3
MEK5
Erk5
+p
homo/heterodimers
rAPS
SH2B
GRB2
SOS
Ras
Raf
MEK1/2
Erk1/2
p38
MAPKAPK2
p90RSK
MSK1
CREB
DNA
Bcl-2
Cellular differentiation
Cell survival
Retrograde transport
Axonal outgrowth
SH-PTP2
cAbl
Ubiquitin mediated proteolysis
NGF
BDNF
NT4
NT3
TrkA
TrkB
TrkC
Shc
Gab1
PI3K
PIP3
Akt
PDK
IκB
NFκB
Retrograde transport
Cell survival
IRS1
CHK
FKHRL1
FasL
Bad
GSK3β
PLCγ
DAG
PKCδ
IP3
Ca2+
CaM
CaMK
CREB
Plasticity
Long-term potentiation
ceramide
RhoGDI
RhoA
Regulation of actin cytoskeleton
Membrane ruffling
Axonal outgrowth
Axonal guidance
Axon patterning
Synapse formation
p75βP
p75ICD
PSEN
γ-secretase
extracellular domain
p75CTF
SC-1
NRAGE
Cell cycle arrest
TRAF6
+u
NRIF
p75NTR
p73
p53
Bax
Apoptosis
Cdc42
Rac1
MEKK1
ASK1
MKK7
JNK
c-Jun
NADE
14-3-3ε
Sortilin
RIP2
IRAK
TRAF6
IKKβ
IκB
NFκB
Cell survival
Data on KEGG graph
Rendered by Pathview

S6B Fig

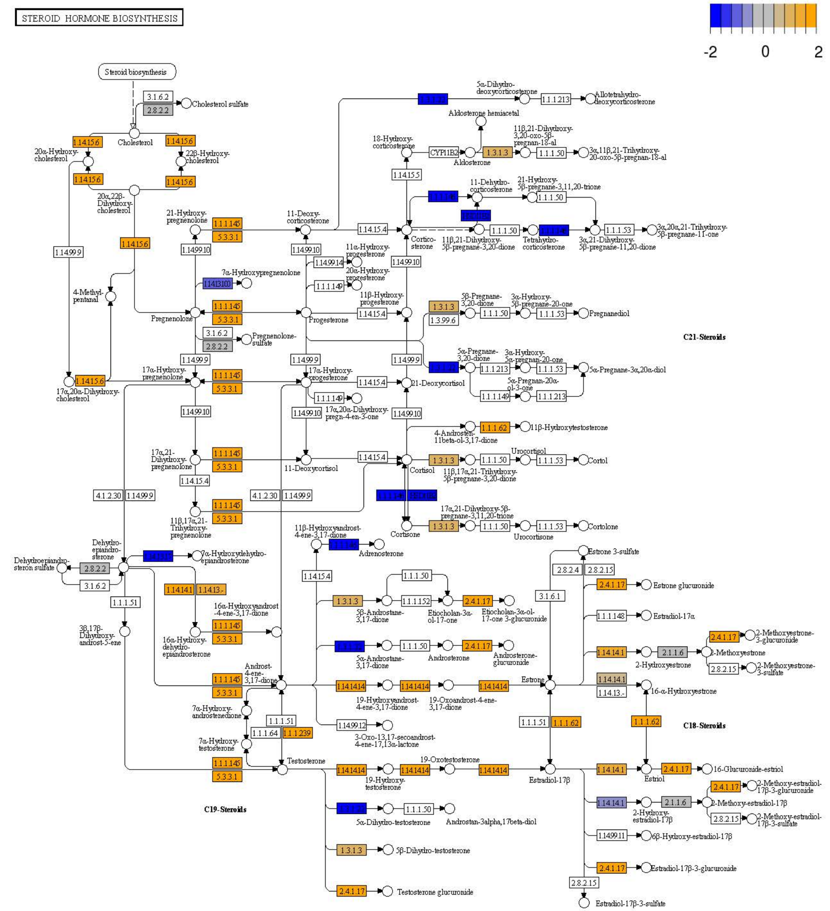


Data on KEGG graph
Rendered by Pathview

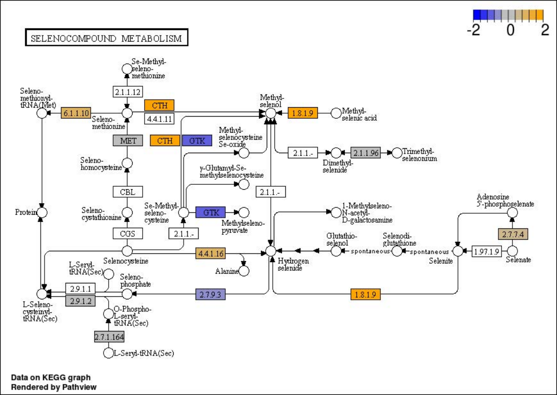

-2 0 2
SELENOCOMPOUND METABOLISM
Se-Methyl-seleno-methionine
2.1.1.12
Seleno-methionyl-tRNA(Met)
6.1.1.10
Seleno-methionine
CTH
4.4.1.11
Methyl-selenol
1.8.1.9
Methyl-selenic acid
MET
CTH
GTK
Methyl-selenocysteine Se-oxide
2.1.1.-
2.1.1.96
Trimethyl-selenonium
Dimethyl-selenide
Seleno-homocysteine
γ-Glutamyl-Se-methylselenocysteine
CBL
2.1.1.-
Protein
Seleno-cystathionine
Se-Methyl-seleno-cysteine
GTK
Methylseleno-pyruvate
1-Methylseleno-N-acetyl-D-galactosamine
Adenosine 5'-phosphoselenate
CGS
2.1.1.-
2.7.7.4
Glutathio-selenol
Selenodi-glutathione
spontaneous
spontaneous
Selenite
1.97.1.9
Selenate
Selenocysteine
4.4.1.16
Hydrogen selenide
L-Seryl-tRNA(Sec)
Alanine
Seleno-phosphate
2.9.1.1
2.9.1.2
L-Seleno-cysteinyl-tRNA(Sec)
2.7.9.3
1.8.1.9
O-Phospho-L-seryl-tRNA(Sec)
2.7.1.164
L-Seryl-tRNA(Sec)
Data on KEGG graph
Rendered by Pathview

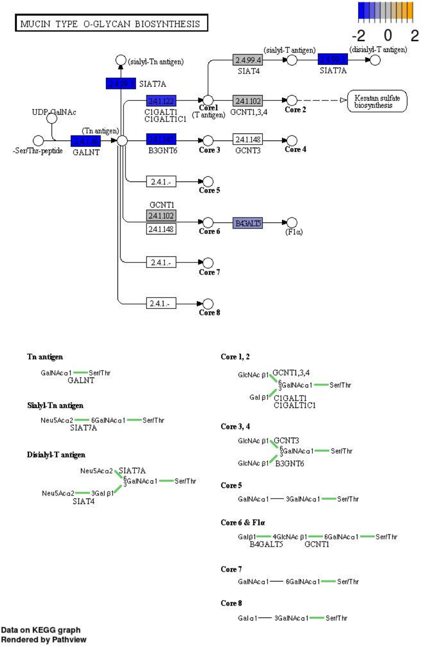
MUCIN TYPE O-GLYCAN BIOSYNTHESIS
-2 0 2
(sialyl-Tn antigen)
SIAT7A
(sialyl-T antigen)
(disialyl-T antigen)
2.4.99.4
SIAT4
SIAT7A
2.4.1.122
C1GALT1
C1GALT1C1
Core1
(T antigen)
2.4.1.102
GCNT1,3,4
Core 2
Keratan sulfate biosynthesis
UDP-GalNAc
(Tn antigen)
-Ser/Thr-peptide
GALNT
B3GNT6
Core 3
2.4.1.148
GCNT3
Core 4
2.4.1.-
Core 5
GCNT1
2.4.1.102
2.4.1.148
Core 6
B4GALT5
(F1α)
2.4.1.-
Core 7
2.4.1.-
Core 8
Tn antigen
GalNAcα1 Ser/Thr
GALNT
Sialyl-Tn antigen
Neu5Acα2 6GalNAcα1 Ser/Thr
SIAT7A
Disialyl-T antigen
Neu5Acα2 SIAT7A
6 3 GalNAcα1 Ser/Thr
Neu5Acα2 3Galβ1
SIAT4
Core 1, 2
GlcNAcβ1 GCNT1,3,4
6 3 GalNAcα1 Ser/Thr
Galβ1 C1GALT1
C1GALT1C1
Core 3, 4
GlcNAcβ1 GCNT3
6 3 GalNAcα1 Ser/Thr
GlcNAcβ1 B3GNT6
Core 5
GalNAcα1 3GalNAcα1 Ser/Thr
Core 6 & F1α
Galβ1 4GlcNAcβ1 6GalNAcα1 Ser/Thr
B4GALT5 GCNT1
Core 7
GalNAcα1 6GalNAcα1 Ser/Thr
Core 8
Galα1 3GalNAcα1 Ser/Thr
Data on KEGG graph
Rendered by Pathview

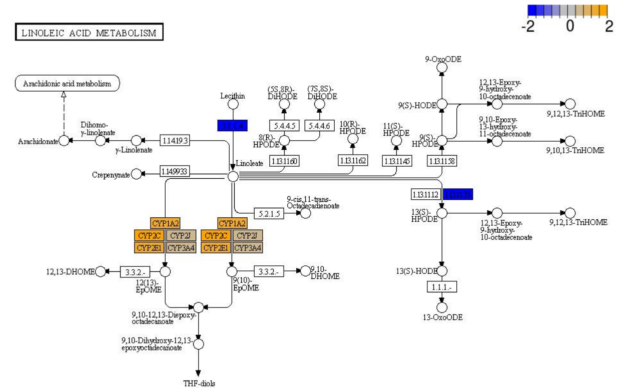


Data on KEGG graph
Rendered by Pathview

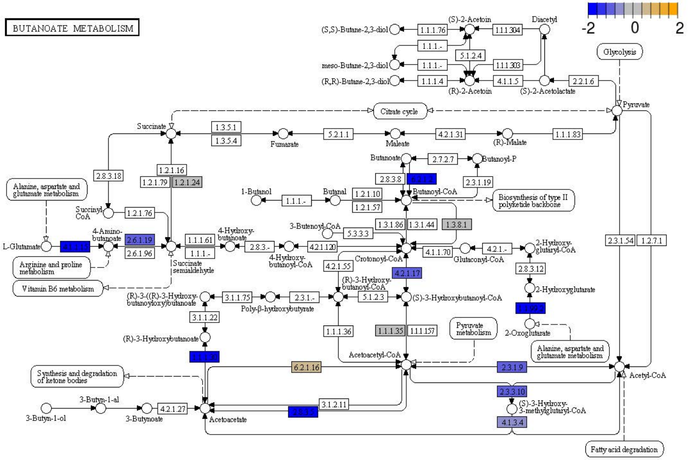


Data on KEGG graph
Rendered by Pathview

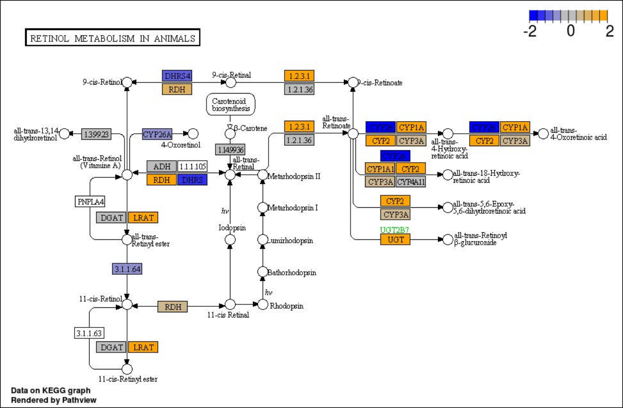
-2
0
2
RETINOL METABOLISM IN ANIMALS
9-cis-Retinol
DHRS4
RDH
9-cis-Retinal
1.2.3.1
1.2.1.36
9-cis-Retinoate
Carotenoid biosynthesis
β-Carotene
1.14.99.36
all-trans-13,14-dihydroretinol
139923
CYP26A
4-Oxoretinol
all-trans-Retinol (Vitamine A)
ADH
1.1.1.105
RDH
DHRS
all-trans-Retinal
1.2.3.1
1.2.1.36
all-trans-Retinoate
CYP26
CYP1A
CYP2
CYP3A
all-trans-4-Hydroxy-retinoic acid
CYP26
CYP1A
CYP2
CYP3A
all-trans-4-Oxoretinoic acid
CYP26
CYP1A1
CYP2
CYP3A
CYP4A11
all-trans-18-Hydroxy-retinoic acid
CYP2
CYP3A
all-trans-5,6-Epoxy-5,6-dihydroretinoic acid
UGT2B7
UGT
all-trans-Retinoyl β-glucuronide
Metarhodopsin II
Metarhodopsin I
Lumirhodopsin
Bathorhodopsin
Rhodopsin
hν
hν
Iodopsin
PNPLA4
DGAT
LRAT
all-trans-Retinyl ester
3.1.1.64
11-cis-Retinol
RDH
11-cis Retinal
3.1.1.63
DGAT
LRAT
11-cis-Retinyl ester
Data on KEGG graph
Rendered by Pathview

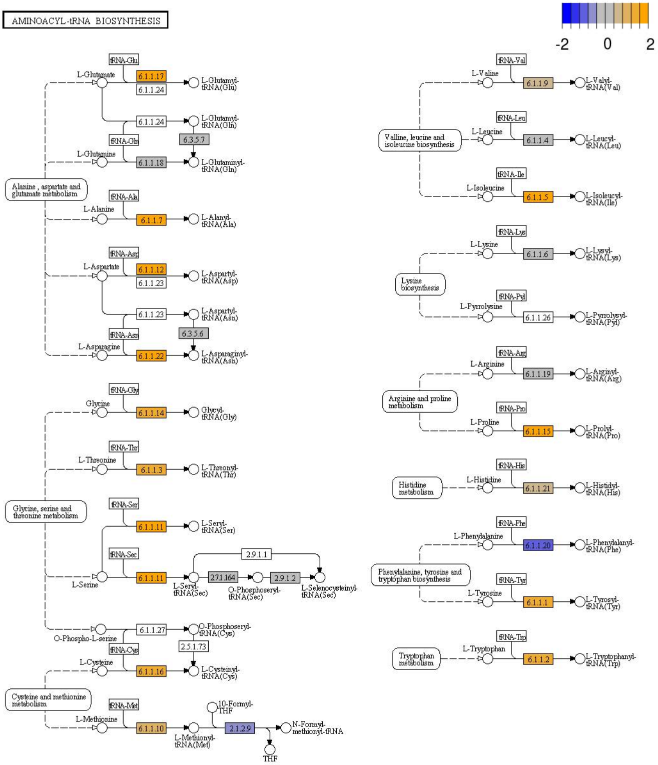


**Data on KEGG graph**
**Rendered by Pathview**

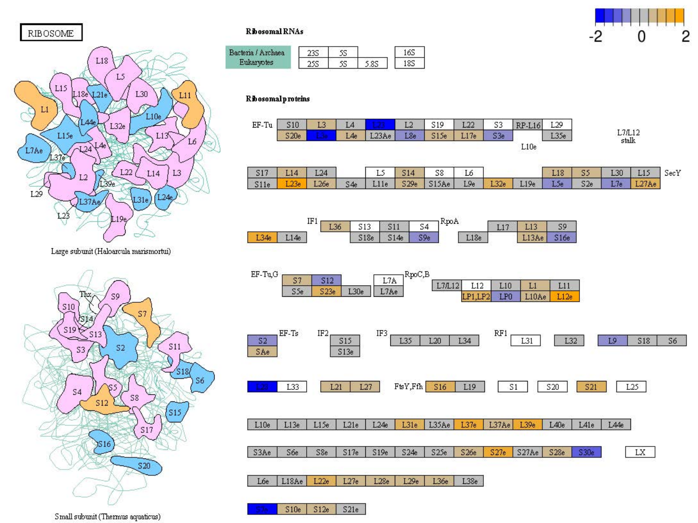


**Data on KEGG graph**
**Rendered by Pathview**

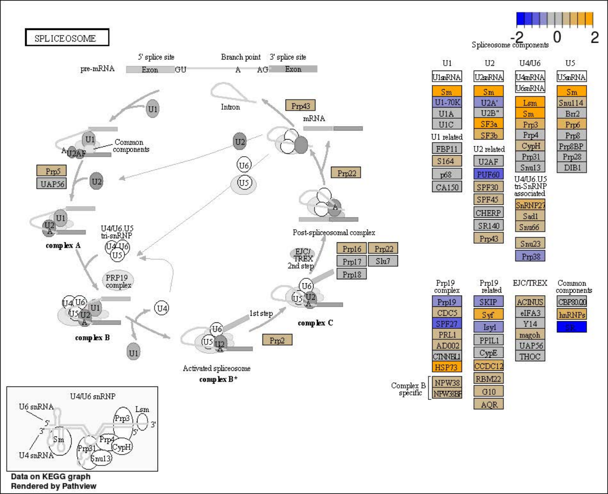

SPLICEOSOME
-2 0 2
Spliceosome components
5' splice site
Branch point
3' splice site
pre-mRNA
Exon
GU
A
AG
Exon
Intron
Prp43
mRNA
U1
U2
U6
U5
Common components
U2AF
Prp5
UAP56
Prp22
complex A
U4/U6.U5 tri-snRNP
Post-spliceosomal complex
EJC/TREX
2nd step
Prp16
Prp22
Prp17
Slu7
Prp18
PRP19 complex
complex B
U4
1st step
complex C
Prp2
Activated spliceosome
complex B*
U4/U6 snRNP
U6 snRNA
U4 snRNA
Sm
Lsm
Prp3
Prp4
CypH
Prp31
Snu13
U1
U2
U4/U6
U5
U1snRNA
Sm
U1-70K
U1A
U1C
U1 related
FBP11
S164
p68
CA150
U2snRNA
Sm
U2A'
U2B"
SF3a
SF3b
U2 related
U2AF
PUF60
SPF30
SPF45
CHERP
SR140
Prp43
U4snRNA
U6snRNA
Lsm
Sm
Prp3
Prp4
CypH
Prp31
Snu13
U4/U6.U5 tri-SnRNP associated
SnRNP27
Sad1
Snu66
Snu23
Prp38
U5snRNA
Sm
Snu114
Brr2
Prp6
Prp8
Prp8BP
Prp28
DIB1
Prp19 complex
Prp19
CDC5
SPF27
PRL1
AD002
CTNNBL1
HSP73
Complex B specific
NPW38
NPW38BP
Prp19 related
SKIP
Syf
Isy1
PPIL1
CypE
CCDC12
RBM22
G10
AQR
EJC/TREX
ACINUS
eIFA3
Y14
magoh
UAP56
THOC
Common components
CBP80/20
hnRNPs
SR
Data on KEGG graph
Rendered by Pathview

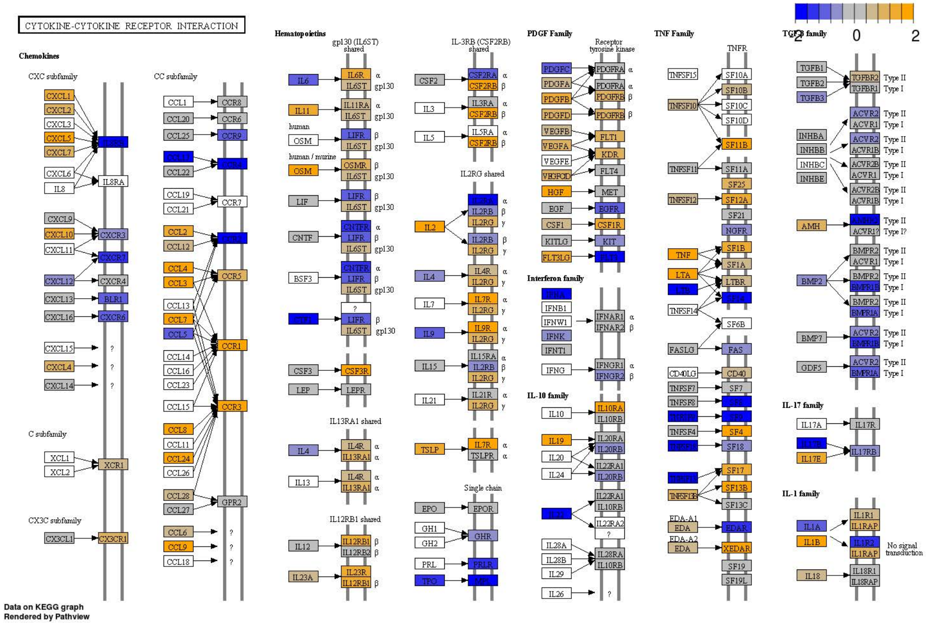
CYTOKINE-CYTOKINE RECEPTOR INTERACTION
-2
0
2
Chemokines
CXC subfamily
CXCL1
CXCL2
CXCL3
CXCL5
CXCL7
CXCL6
IL8
IL8RA
CXCL9
CXCL10
CXCL11
CXCR3
CXCR7
CXCL12
CXCR4
CXCL13
BLR1
CXCL16
CXCR6
CXCL15
CXCL4
CXCL14
C subfamily
XCL1
XCL2
XCR1
CX3C subfamily
CX3CL1
CX3CR1
CC subfamily
CCL1
CCR8
CCL20
CCR6
CCL25
CCR9
CCL22
CCL19
CCL21
CCR7
CCL2
CCL12
CCL4
CCL3
CCR5
CCL13
CCL7
CCL5
CCR1
CCL14
CCL16
CCL23
CCL15
CCR3
CCL8
CCL11
CCL24
CCL26
CCL28
CCL27
GPR2
CCL6
CCL9
CCL18
Hematopoietins
gp130 (IL6ST) shared
IL6
IL6R
IL6ST
IL11
IL11RA
human
OSM
LIFR
human / murine
OSMR
LIF
CNTF
CNTFR
BSF3
CSF3
CSF3R
LEP
LEPR
IL13RA1 shared
IL4
IL4R
IL13RA1
IL13
IL12RB1 shared
IL12
IL12RB1
IL12RB2
IL23A
IL23R
IL-3RB (CSF2RB) shared
CSF2
CSF2RA
CSF2RB
IL3
IL3RA
IL5
IL5RA
IL2RG shared
IL2
IL2RB
IL2RG
IL4R
IL7
IL7R
IL9
IL9R
IL15
IL15RA
IL21
IL21R
TSLP
TSLPR
Single chain
EPO
EPOR
GH1
GH2
GHR
PRL
PRLR
TPO
MPL
PDGF Family
Receptor tyrosine kinase
PDGFC
PDGFRA
PDGFA
PDGFB
PDGFRB
PDGFD
VEGFB
FLT1
VEGFA
KDR
VEGFE
VEGFD
FLT4
HGF
MET
EGF
EGFR
CSF1
CSF1R
KITLG
KIT
FLT3LG
FLT3
Interferon family
IFNB1
IFNW1
IFNAR1
IFNAR2
IFNK
IFNT1
IFNG
IFNGR1
IFNGR2
IL-10 family
IL10
IL10RA
IL10RB
IL19
IL20RA
IL20RB
IL20
IL22RA1
IL24
IL22RA2
IL28A
IL28B
IL28RA
IL29
IL26
TNF Family
TNFR
TNFSF15
SF10A
SF10B
TNFSF10
SF10C
SF10D
SF11B
TNFSF11
SF11A
SF25
TNFSF12
SF12A
SF21
NGFR
TNF
SF1B
SF1A
LTA
LTBR
TNFSF14
SF6B
FASLG
FAS
CD40LG
CD40
TNFSF7
SF7
TNFSF8
TNFSF4
SF4
SF18
SF17
SF13B
SF13C
EDA-A1
EDA
EDAR
EDA-A2
XEDAR
SF19
SF19L
TGF-β family
TGFB1
TGFBR2
TGFB2
TGFBR1
TGFB3
Type II
Type I
ACVR2
ACVR1
INHBA
INHBB
ACVR1B
INHBC
ACVR2B
INHBE
AMH
ACVR1?
Type I?
BMPR2
BMP2
BMPR1B
BMPR1A
BMP7
GDF5
IL-17 family
IL17A
IL17R
IL17E
IL17RB
IL-1 family
IL1R1
IL1RAP
IL1A
IL1B
IL1R2
No signal transduction
IL18
IL18R1
IL18RAP
Data on KEGG graph
Rendered by Pathview

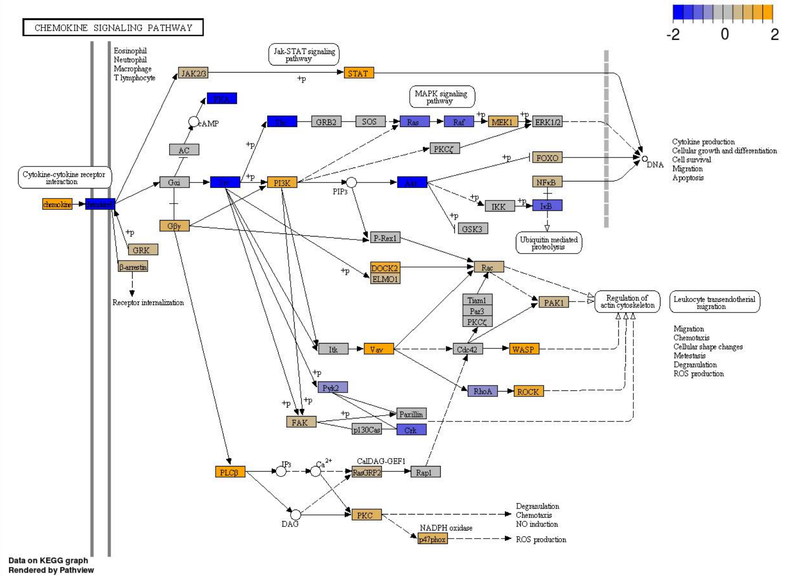

CHEMOKINE SIGNALING PATHWAY
-2
0
2
Eosinophil
Neutrophil
Macrophage
T lymphocyte
Jak-STAT signaling pathway
JAK2/3
STAT
+p
MAPK signaling pathway
PKA
cAMP
AC
Shc
GRB2
SOS
Ras
Raf
MEK1
ERK1/2
PKCζ
FOXO
Cytokine production
Cellular growth and differentiation
Cell survival
Migration
Apoptosis
DNA
Cytokine-cytokine receptor interaction
Gαi
Src
PI3K
PIP3
Akt
NFκB
IKK
IκB
GSK3
chemokine
Receptor
GRK
β-arrestin
Receptor internalization
Gβγ
P-Rex1
Ubiquitin mediated proteolysis
DOCK2
ELMO1
Rac
Tiam1
Par3
PKCζ
PAK1
Regulation of actin cytoskeleton
Leukocyte transendothelial migration
Migration
Chemotaxis
Cellular shape changes
Metestasis
Degranulation
ROS production
Itk
Vav
Cdc42
WASP
RhoA
ROCK
Pyk2
Paxillin
FAK
p130Cas
Crk
PLCβ
IP3
Ca2+
CalDAG-GEF1
RasGRP2
Rap1
DAG
PKC
Degranulation
Chemotaxis
NO induction
NADPH oxidase
p47phox
ROS production
Data on KEGG graph
Rendered by Pathview

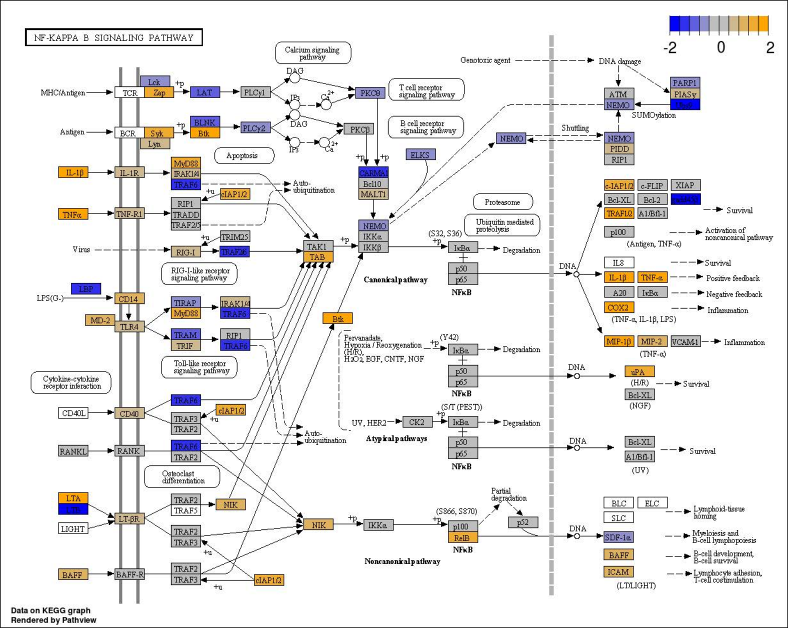

NF-KAPPA B SIGNALING PATHWAY
-2
0
2
MHC/Antigen
TCR
Lck
Zap
LAT
PLCγ1
Calcium signaling pathway
DAG
IP3
Ca2+
PKCθ
T cell receptor signaling pathway
Antigen
BCR
Syk
Lyn
BLNK
Btk
PLCγ2
PKCβ
B cell receptor signaling pathway
Apoptosis
IL-1β
IL-1R
MyD88
IRAK1/4
TRAF6
Auto-ubiquitination
cIAP1/2
TNFα
TNF-R1
RIP1
TRADD
TRAF2/5
CARMA1
Bcl10
MALT1
ELKS
NEMO
Genotoxic agent
DNA damage
ATM
PARP1
PIASy
Ubc9
SUMOylation
Shuttling
PIDD
Proteasome
Ubiquitin mediated proteolysis
Virus
RIG-I
TRIM25
TRAF2/6
TAK1
TAB
IKKα
IKKβ
(S32, S36)
IκBα
Degradation
p50
p65
NFκB
Canonical pathway
RIG-I-like receptor signaling pathway
LBP
LPS(G-)
CD14
MD-2
TLR4
TIRAP
TRAM
TRIF
Btk
Toll-like receptor signaling pathway
Cytokine-cytokine receptor interaction
Pervanadate, Hypoxia / Reoxygenation (H/R), H2O2, EGF, CNTF, NGF
(Y42)
UV, HER2
CK2
(S/T (PEST))
Atypical pathways
CD40L
CD40
TRAF3
TRAF2
RANKL
RANK
Osteoclast differentiation
LTA
LTB
LIGHT
LT-βR
TRAF5
NIK
BAFF
BAFF-R
(S866, S870)
p100
RelB
Partial degradation
p52
Noncanonical pathway
DNA
c-IAP1/2
c-FLIP
XIAP
Bcl-XL
Bcl-2
TRAF1/2
A1/Bfl-1
Survival
p100
Activation of noncanonical pathway
(Antigen, TNF-α)
IL8
IL-1β
TNF-α
A20
IκBα
COX2
Positive feedback
Negative feedback
Inflammation
(TNF-α, IL-1β, LPS)
MIP-1β
MIP-2
VCAM-1
(TNF-α)
uPA
(H/R)
(NGF)
(UV)
BLC
ELC
SLC
SDF-1α
ICAM
(LT/LIGHT)
Lymphoid-tissue homing
Myeloiesis and B-cell lymphopoiesis
B-cell development, B-cell survival
Lymphocyte adhesion, T-cell costimulation
Data on KEGG graph
Rendered by Pathview

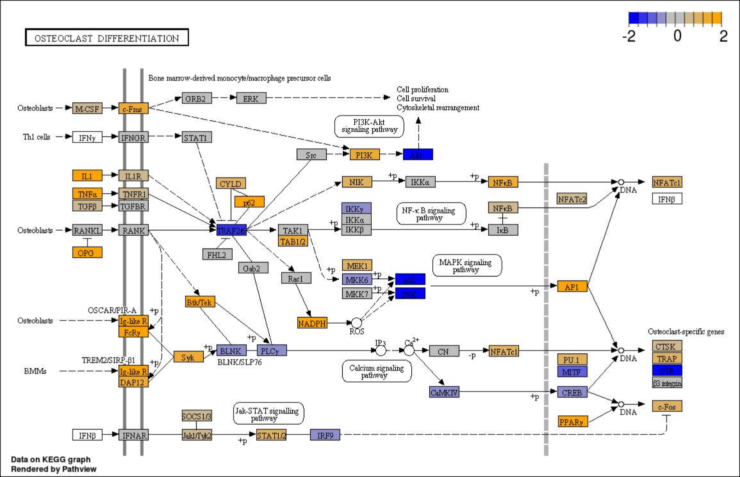
OSTEOCLAST DIFFERENTIATION
-2
0
2
Bone marrow-derived monocyte/macrophage precursor cells
Osteoblasts
M-CSF
c-Fms
GRB2
ERK
Cell proliferation
Cell survival
Cytoskeletal rearrangement
PI3K-Akt signaling pathway
Th1 cells
IFNγ
IFNGR
STAT1
Src
PI3K
Akt
IL1
IL1R
CYLD
NIK
+p
IKKα
NFκB
NFATc1
DNA
TNFα
TNFR1
p62
IFNβ
TGFβ
TGFBR
IKKγ
IKKα
IKKβ
NF-κB signaling pathway
NFκB
NFATc2
RANKL
RANK
TRAF2/6
TAK1
TAB1/2
IκB
OPG
FHL2
Gab2
MEK1
MAPK signaling pathway
Rac1
MKK6
p38
MKK7
JNK
AP1
OSCAR/PIR-A
Btk/Tek
Ig-like R
FcRγ
NADPH
ROS
Osteoclast-specific genes
CTSK
TRAP
CTR
β3 integrin
Syk
BLNK
PLCγ
IP3
Ca2+
CN
NFATc1
-p
PU.1
MITF
BLNK/SLP76
TREM2/SIRP-β1
BMMs
DAP12
Calcium signaling pathway
CaMKIV
CREB
SOCS1/3
Jak-STAT signalling pathway
c-Fos
PPARγ
Jak1/Tyk2
STAT1/2
IRF9
IFNAR
Data on KEGG graph
Rendered by Pathview

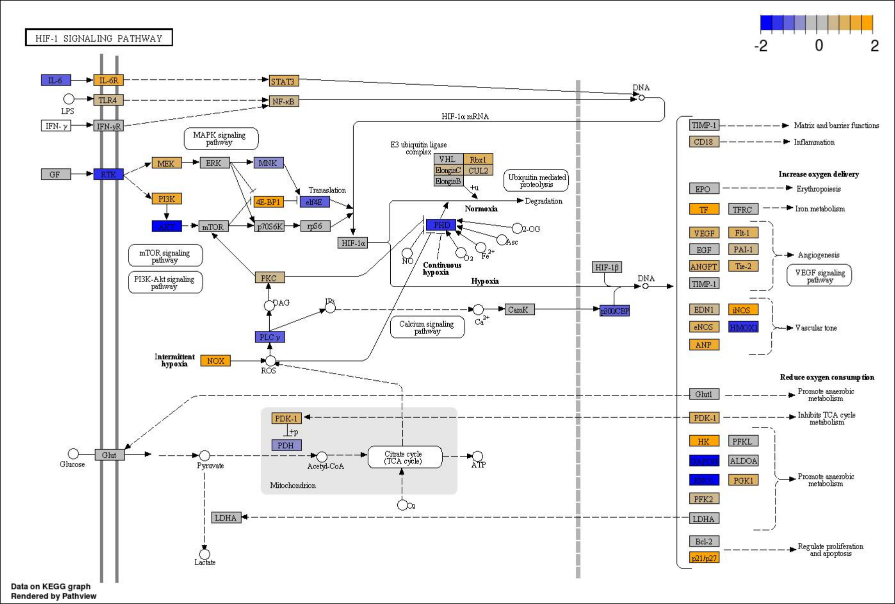
HIF-1 SIGNALING PATHWAY
-2
0
2
IL-6
IL-6R
STAT3
LPS
TLR4
NF-κB
IFN-γ
IFN-γR
DNA
HIF-1α mRNA
MAPK signaling pathway
GF
RTK
MEK
ERK
MNK
E3 ubiquitin ligase complex
VHL
Rbx1
ElonginC
CUL2
ElonginB
+u
Ubiquitin mediated proteolysis
Translation
PI3K
4E-BP1
eIF4E
Degradation
Normoxia
AKT
mTOR
p70S6K
rpS6
PHD
2-OG
HIF-1α
Asc
NO
O2
Fe2+
Continuous hypoxia
mTOR signaling pathway
PI3K-Akt signaling pathway
PKC
Hypoxia
HIF-1β
DNA
DAG
IP3
Ca2+
CamK
p300/CBP
Calcium signaling pathway
PLCγ
Intermittent hypoxia
NOX
ROS
TIMP-1
Matrix and barrier functions
CD18
Inflammation
Increase oxygen delivery
EPO
Erythropoiesis
TF
TFRC
Iron metabolism
VEGF
Flt-1
EGF
PAI-1
Angiogenesis
ANGPT
Tie-2
VEGF signaling pathway
TIMP-1
EDN1
iNOS
eNOS
HMOX1
Vascular tone
ANP
Reduce oxygen consumption
Glut1
Promote anaerobic metabolism
PDK-1
Inhibits TCA cycle metabolism
PDK-1
+p
PDH
Glucose
Glut
Pyruvate
Acetyl-CoA
Citrate cycle (TCA cycle)
ATP
Mitochondrion
O2
HK
PFKL
GAPDH
ALDOA
ENO1
PGK1
Promote anaerobic metabolism
PFK2
LDHA
LDHA
Lactate
Bcl-2
p21/p27
Regulate proliferation and apoptosis
Data on KEGG graph
Rendered by Pathview

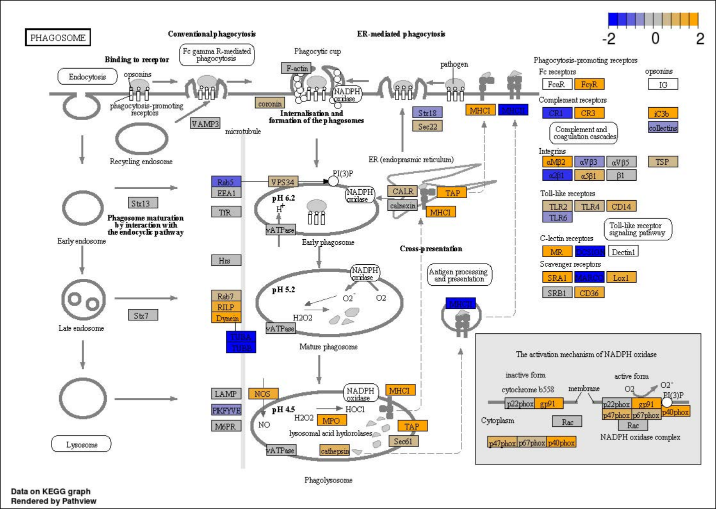

PHAGOSOME
-2
0
2
Conventional phagocytosis
ER-mediated phagocytosis
Fc gamma R-mediated phagocytosis
Phagocytic cup
Binding to receptor
Endocytosis
opsonins
phagocytosis-promoting receptors
F-actin
NADPH oxidase
pathogen
coronin
Internalisation and formation of the phagosomes
VAMP3
microtubule
Recycling endosome
Stx18
Sec22
MHCI
MHCII
ER (endoplasmic reticulum)
Phagocytosis-promoting receptors
Fc receptors
FcαR
FcγR
opsonins
IG
Complement receptors
CR1
CR3
iC3b
Complement and coagulation cascades
collectins
Integrins
αMβ2
αVβ3
αVβ5
TSP
α2β1
α5β1
β1
Toll-like receptors
TLR2
TLR4
CD14
TLR6
Toll-like receptor signaling pathway
C-lectin receptors
MR
DCSIGN
Dectin1
Scavenger receptors
SRA1
MARCO
Lox1
SRB1
CD36
Rab5
EEA1
VPS34
PI(3)P
pH 6.2
$H^+$
NADPH oxidase
CALR
calnexin
TAP
MHCI
Stx13
TfR
vATPase
Phagosome maturation by interaction with the endocytic pathway
Early endosome
Early phagosome
Cross-presentation
Hrs
NADPH oxidase
Antigen processing and presentation
Rab7
RILP
Dynein
pH 5.2
$O_2^-$
O2
H2O2
MHCII
Stx7
Late endosome
TUBA
TUBB
vATPase
Mature phagosome
LAMP
NOS
NADPH oxidase
MHCI
PIKFYVE
pH 4.5
HOCl
H2O2
MPO
M6PR
NO
lysosomal acid hydrolases
TAP
Sec61
Lysosome
vATPase
cathepsin
Phagolysosome
The activation mechanism of NADPH oxidase
inactive form
active form
cytochrome b558
membrane
O2
$O_2^-$
PI(3)P
p22phox
gp91
p22phox
gp91
p47phox
p67phox
p40phox
Cytoplasm
Rac
Rac
NADPH oxidase complex
p47phox
p67phox
p40phox
Data on KEGG graph
Rendered by Pathview

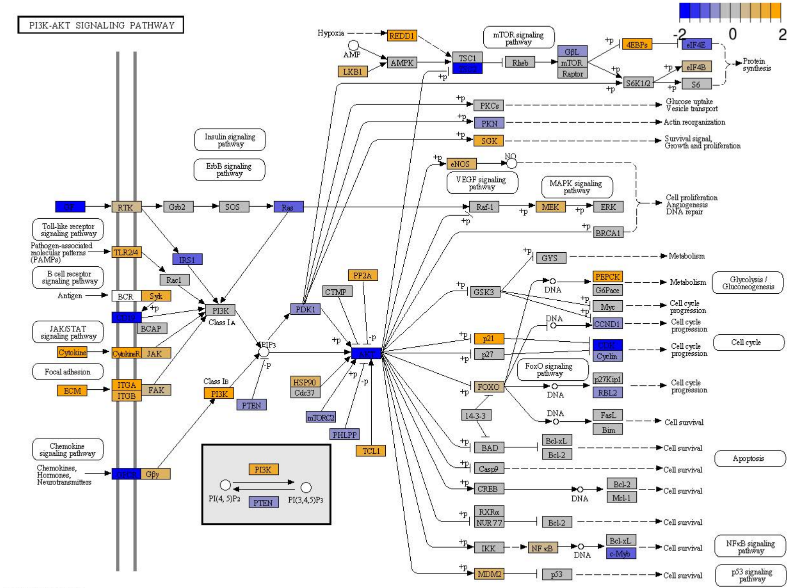


Data on KEGG graph
Rendered by Pathview

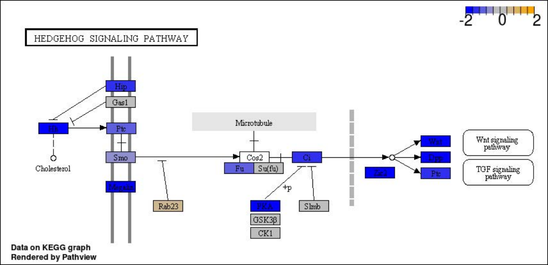

-2
0
2
HEDGEHOG SIGNALING PATHWAY
Hip
Gas1
Hh
Ptc
Cholesterol
Smo
Megalin
Rab23
Microtubule
Cos2
Fu
Su(fu)
Ci
+p
PKA
GSK3β
CK1
Slmb
Zic2
Wnt
Dpp
Ptc
Wnt signaling pathway
TGF signaling pathway
Data on KEGG graph
Rendered by Pathview

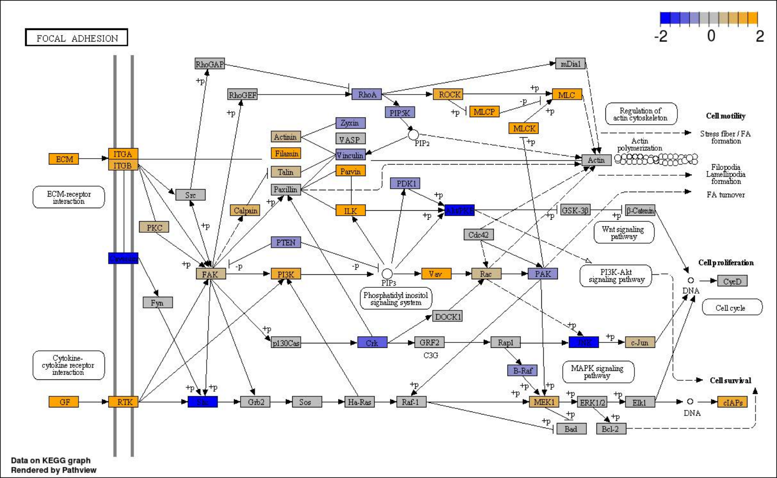

-2
0
2
FOCAL ADHESION
RhoGAP
mDia1
RhoGEF
RhoA
ROCK
MLC
MLCP
PIP5K
Zyxin
VASP
Actinin
Filamin
Vinculin
Talin
Parvin
Paxillin
PIP2
MLCK
Regulation of actin cytoskeleton
Cell motility
Stress fiber / FA formation
Actin polymerization
Actin
Filopodia Lamellipodia formation
FA turnover
ECM
ITGA
ITGB
ECM-receptor interaction
Src
PKC
Calpain
ILK
PDK1
GSK-3β
β-Catenin
Wnt signaling pathway
Cdc42
PTEN
FAK
PI3K
PIP3
Vav
Rac
PAK
PI3K-Akt signaling pathway
Cell proliferation
DNA
CycD
Cell cycle
Fyn
Phosphatidyl inositol signaling system
DOCK1
p130Cas
Crk
GRF2
C3G
Rap1
c-Jun
B-Raf
MAPK signaling pathway
Cell survival
Cytokine-cytokine receptor interaction
GF
RTK
Grb2
Sos
Ha-Ras
Raf-1
MEK1
ERK1/2
Elk1
cIAPs
Bad
Bcl-2
Data on KEGG graph
Rendered by Pathview

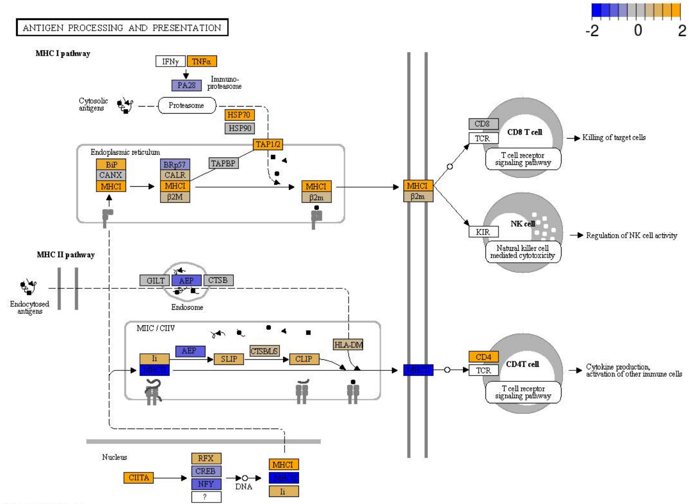


Data on KEGG graph
Rendered by Pathview

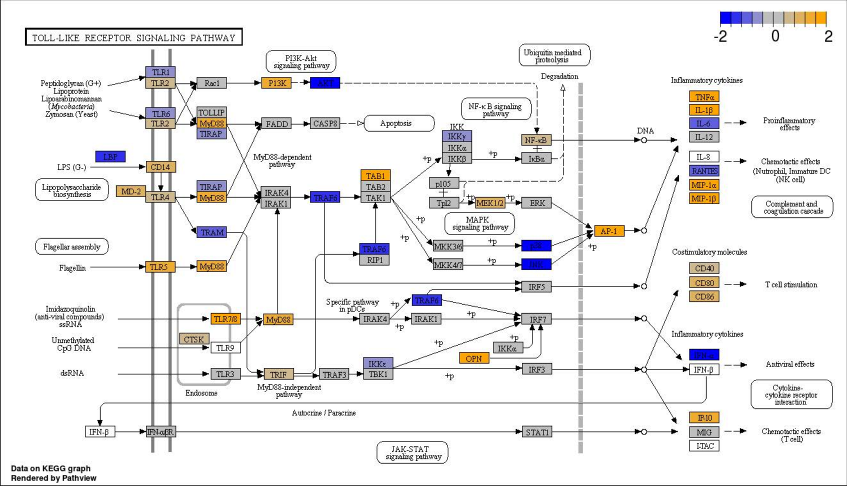

TOLL-LIKE RECEPTOR SIGNALING PATHWAY
-2
0
2
Peptidoglycan (G+)
Lipoprotein
Lipoarabinomannan
(Mycobacteria)
Zymosan (Yeast)
TLR1
TLR2
TLR6
TLR2
Rac1
PI3K-Akt signaling pathway
PI3K
TOLLIP
MyD88
TIRAP
FADD
CASP8
Apoptosis
Ubiquitin mediated proteolysis
Degradation
NF-κB signaling pathway
IKK
IKKγ
IKKα
IKKβ
NF-κB
IκBα
+p
DNA
Inflammatory cytokines
TNFα
IL-1β
IL-6
IL-12
Proinflammatory effects
IL-8
RANTES
MIP-1α
MIP-1β
Chemotactic effects
(Nutrophil, Immature DC
(NK cell)
Complement and coagulation cascade
LBP
LPS (G-)
CD14
MD-2
TLR4
TIRAP
MyD88
Lipopolysaccharide biosynthesis
MyD88-dependent pathway
IRAK4
IRAK1
TRAF6
TAB1
TAB2
TAK1
p105
Tpl2
MEK1/2
ERK
MAPK signaling pathway
AP-1
TRAM
TRAF6
RIP1
MKK3/6
MKK4/7
Flagellar assembly
Flagellin
TLR5
MyD88
IRF5
Costimulatory molecules
CD40
CD80
CD86
T cell stimulation
Imidazoquinolin
(anti-viral compounds)
ssRNA
TLR7/8
MyD88
Specific pathway in pDCs
IRAK4
IRAK1
TRAF6
IRF7
Unmethylated CpG DNA
CTSK
TLR9
IKKα
OPN
Inflammatory cytokines
dsRNA
TLR3
TRIF
TRAF3
IKKε
TBK1
IRF3
IFN-β
Antiviral effects
Cytokine-cytokine receptor interaction
Endosome
MyD88-independent pathway
Autocrine / Paracrine
IFN-β
IFN-αβR
STAT1
IP-10
MIG
I-TAC
Chemotactic effects
(T cell)
JAK-STAT signaling pathway
Data on KEGG graph
Rendered by Pathview

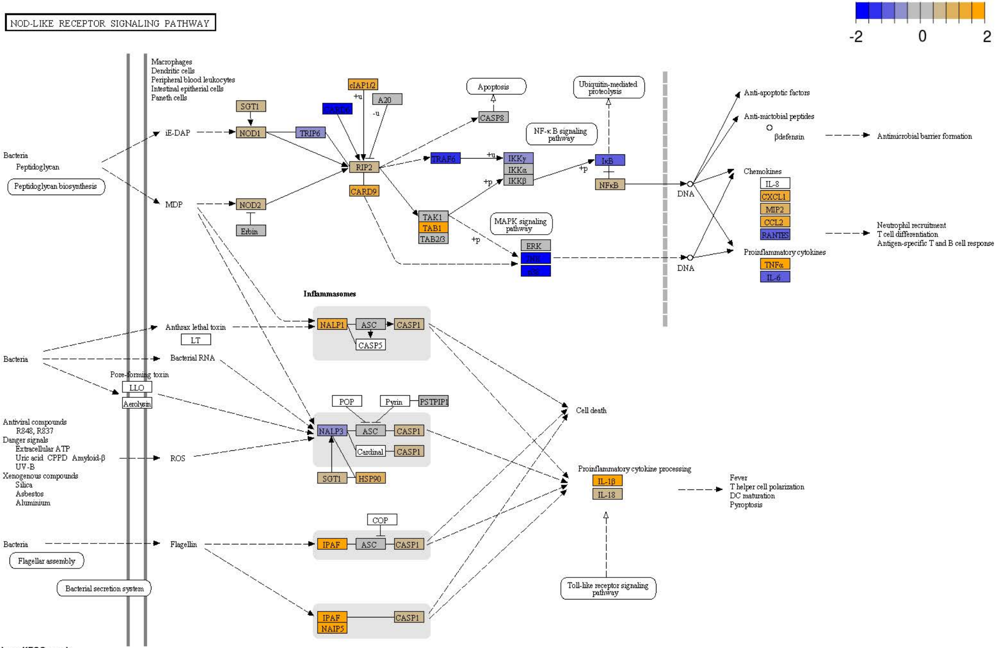


Data on KEGG graph
Rendered by Pathview

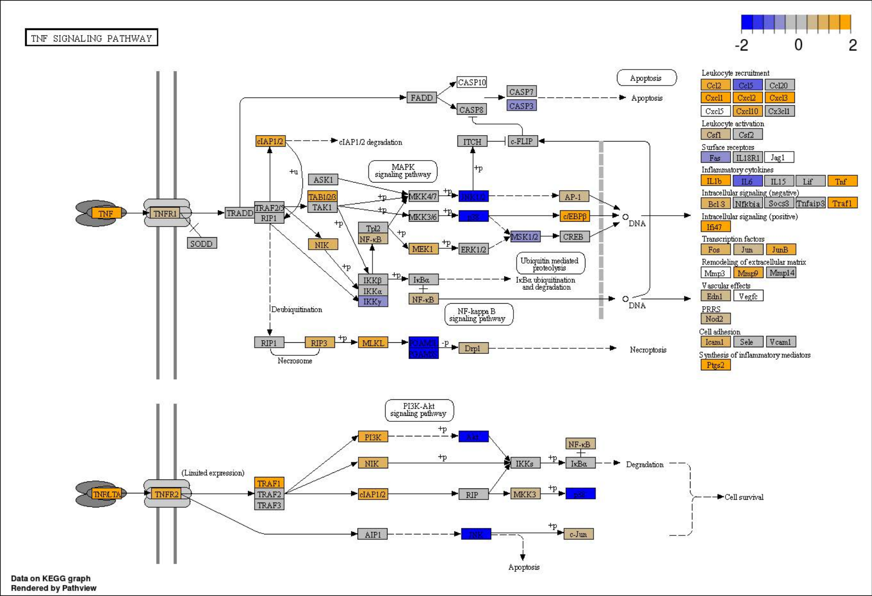
TNF SIGNALING PATHWAY
-2
0
2
TNF
TNFR1
SODD
TRADD
TRAF2/5
RIP1
cIAP1/2
cIAP1/2 degradation
+u
ASK1
TAB1/2/3
TAK1
NIK
Tpl2
NF-κB
IKKβ
IKKα
IKKγ
MAPK signaling pathway
MKK4/7
MKK3/6
MEK1
JNK1/2
p38
ERK1/2
FADD
CASP10
CASP8
CASP7
CASP3
Apoptosis
ITCH
c-FLIP
AP-1
c/EBPβ
MSK1/2
CREB
DNA
IκBα
NF-κB
Ubiquitin mediated proteolysis
IκBα ubiquitination and degradation
NF-kappa B signaling pathway
Deubiquitination
RIP1
RIP3
MLKL
Drp1
Necrosome
Necroptosis
+p
-p
Leukocyte recruitment
Ccl2
Ccl5
Ccl20
Cxcl1
Cxcl2
Cxcl3
Cxcl5
Cxcl10
Cx3cl1
Leukocyte activation
Csf1
Csf2
Surface receptors
Fas
IL18R1
Jag1
Inflammatory cytokines
IL1b
IL6
IL15
Lif
Tnf
Intracellular signaling (negative)
Bcl3
Nfkbia
Socs3
Tnfaip3
Traf1
Intracellular signaling (positive)
Ifi47
Transcription factors
Fos
Jun
JunB
Remodeling of extracellular matrix
Mmp3
Mmp9
Mmp14
Vascular effects
Edn1
Vegfc
PRRS
Nod2
Cell adhesion
Icam1
Sele
Vcam1
Synthesis of inflammatory mediators
Ptgs2
PI3K-Akt signaling pathway
TNF/LTA
TNFR2
(Limited expression)
TRAF1
TRAF2
TRAF3
PI3K
NIK
cIAP1/2
Akt
IKKs
NF-κB
IκBα
Degradation
RIP
MKK3
p38
Cell survival
AIP1
JNK
c-Jun
Apoptosis
Data on KEGG graph
Rendered by Pathview
