# Supplementary material for: Probing the changes in gene expression due to α-crystallin mutations in mouse models of hereditary human cataract
Source: PLoS One. 2018 Jan 16;13(1):e0190817. doi: 10.1371/journal.pone.0190817 (PMC5770019; doi:10.1371/journal.pone.0190817)
Supplement: S7 Fig — Data are plotted on a compressed mean log 2 fold-change scale. (A) Cryab-R120G-het vs. WT lenses; (B) Cryab-R120G-homo vs. WT lenses. (PDF) [file pone.0190817.s007.pdf]

S7A Fig

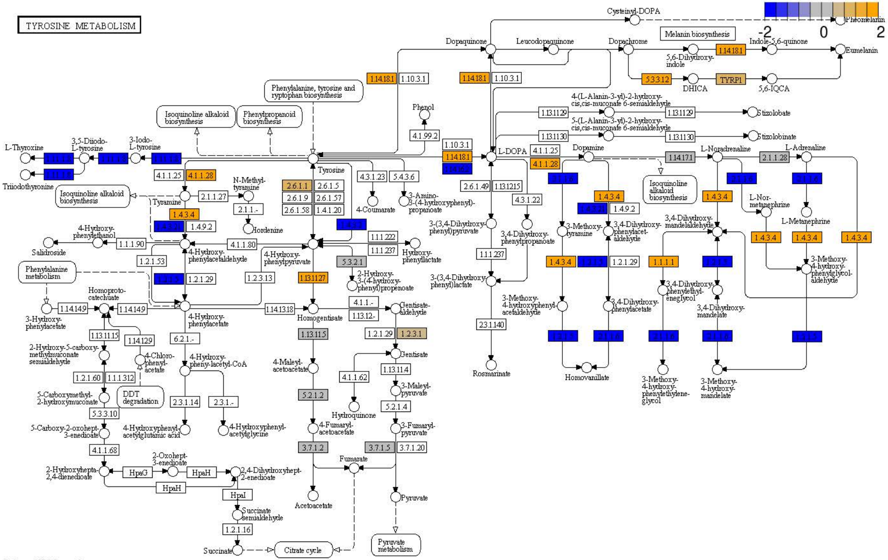

Data on KEGG graph
Rendered by Pathview

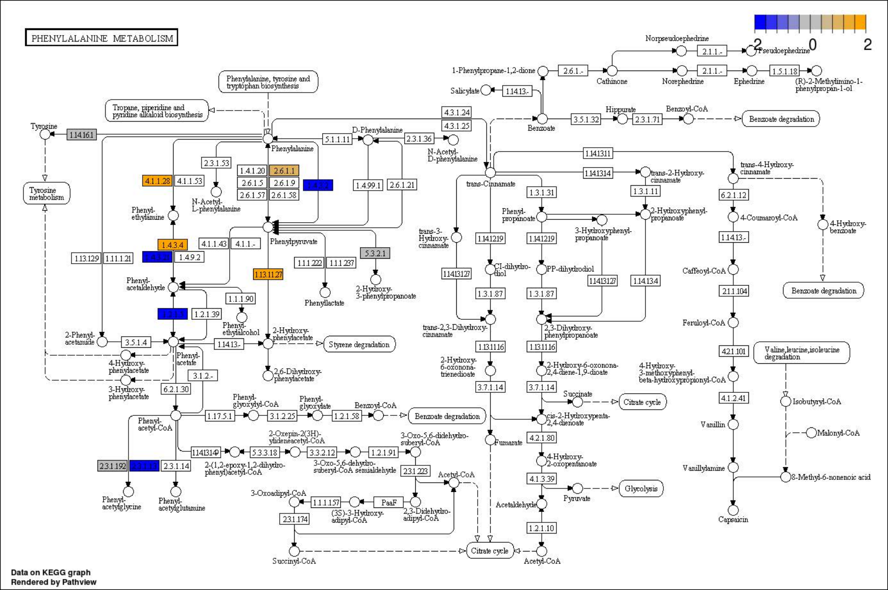
PHENYLALANINE METABOLISM
-2
0
2
Norpseudoephedrine
2.1.1.-
Pseudoephedrine
1-Phenylpropane-1,2-dione
2.6.1.-
Cathinone
2.1.1.-
Norephedrine
Ephedrine
1.5.1.18
(R)-2-Methylimino-1-phenylpropan-1-ol
Salicylate
1.14.13.-
Phenylalanine, tyrosine and tryptophan biosynthesis
Tropane, piperidine and pyridine alkaloid biosynthesis
4.3.1.24
4.3.1.25
Benzoate
3.5.1.32
Hippurate
2.3.1.71
Benzoyl-CoA
Benzoate degradation
Tyrosine
1.14.16.1
Phenylalanine
5.1.1.11
D-Phenylalanine
2.3.1.36
N-Acetyl-D-phenylalanine
1.14.13.11
trans-Cinnamate
1.14.13.14
trans-2-Hydroxy-cinnamate
trans-4-Hydroxy-cinnamate
Tyrosine metabolism
2.3.1.53
1.4.1.20
2.6.1.1
2.6.1.5
2.6.1.9
2.6.1.57
2.6.1.58
4.1.1.28
4.1.1.53
N-Acetyl-L-phenylalanine
1.4.99.1
2.6.1.21
1.3.1.31
1.3.1.11
6.2.1.12
Phenyl-ethylamine
Phenyl-propanoate
3-Hydroxyphenyl-propanoate
2-Hydroxyphenyl-propanoate
4-Coumaroyl-CoA
4-Hydroxy-benzoate
Phenylpyruvate
trans-3-Hydroxy-cinnamate
1.14.12.19
1.14.12.19
1.14.13.-
1.4.3.4
4.1.1.43
4.1.1.-
5.3.2.1
1.13.12.9
1.11.1.21
1.4.9.2
1.1.1.222
1.1.1.237
CI-dihydro-diol
PP-dihydrodiol
Caffeoyl-CoA
1.13.11.27
1.14.13.127
1.14.13.127
1.14.13.4
2.1.1.104
Benzoate degradation
Phenyl-acetaldehyde
2-Hydroxy-3-phenylpropanoate
Phenyllactate
1.1.1.90
1.3.1.87
1.3.1.87
1.2.1.39
Phenyl-ethylalcohol
Feruloyl-CoA
2-Phenyl-acetamide
3.5.1.4
2-Hydroxy-phenylacetate
Styrene degradation
trans-2,3-Dihydroxy-cinnamate
2,3-Dihydroxy-phenylpropanoate
1.14.13.-
1.13.11.16
1.13.11.16
4.2.1.101
Valine, leucine, isoleucine degradation
Phenyl-acetate
4-Hydroxy-phenylacetate
2,6-Dihydroxy-phenylacetate
2-Hydroxy-6-oxonona-trienedioate
2-Hydroxy-6-oxonona-2,4-diene-1,9-dioate
4-Hydroxy-3-methoxyphenyl-beta-hydroxypropionyl-CoA
3.1.2.-
3-Hydroxy-phenylacetate
3.7.1.14
3.7.1.14
Succinate
Citrate cycle
4.1.2.41
Isobutyryl-CoA
6.2.1.30
Phenyl-glyoxylyl-CoA
Phenyl-glyoxylate
Benzoyl-CoA
Benzoate degradation
Phenyl-acetyl-CoA
1.17.5.1
3.1.2.25
1.2.1.58
cis-2-Hydroxypenta-2,4-dienoate
Vanillin
Malonyl-CoA
2-Oxepin-2(3H)-ylideneacetyl-CoA
3-Oxo-5,6-didehydro-suberyl-CoA
4.2.1.80
Fumarate
1.14.13.149
5.3.3.18
3.3.2.12
1.2.1.91
2.3.1.192
2.3.1.14
2-(1,2-epoxy-1,2-dihydro-phenyl)acetyl-CoA
3-Oxo-5,6-dehydro-suberyl-CoA semialdehyde
2.3.1.223
Acetyl-CoA
4-Hydroxy-2-oxopentanoate
Vanillylamine
4.1.3.39
8-Methyl-6-nonenoic acid
Pyruvate
Glycolysis
Phenyl-acetylglycine
Phenyl-acetylglutamine
3-Oxoadipyl-CoA
1.1.1.157
PaaF
Acetaldehyde
2.3.1.174
(3S)-3-Hydroxy-adipyl-CoA
2,3-Didehydro-adipyl-CoA
Capsaicin
1.2.1.10
Succinyl-CoA
Citrate cycle
Acetyl-CoA
Data on KEGG graph
Rendered by Pathview

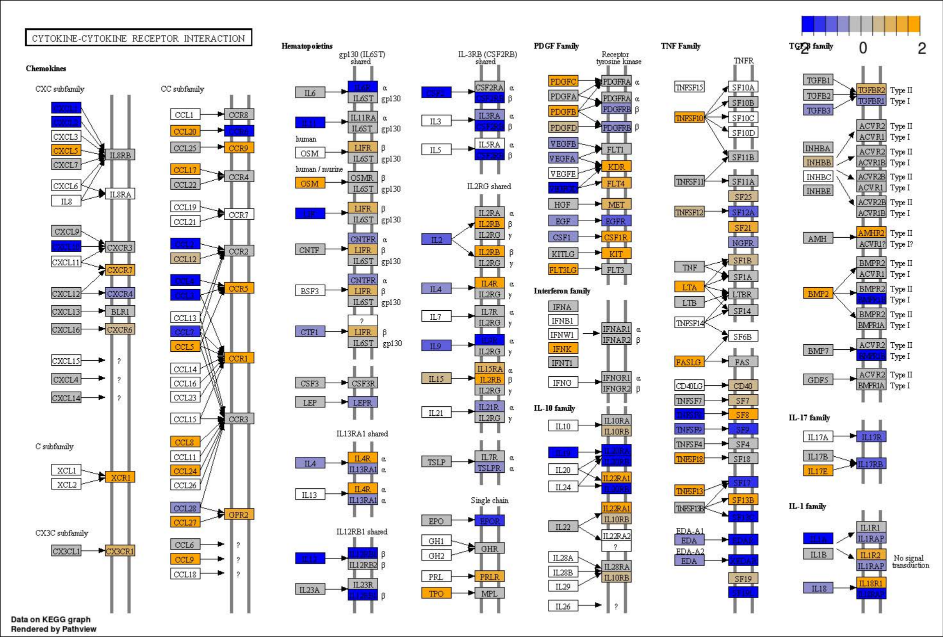
CYTOKINE-CYTOKINE RECEPTOR INTERACTION
-2
0
2
Chemokines
CXC subfamily
CXCL3
CXCL5
CXCL7
CXCL6
IL8
IL8RB
IL8RA
CXCL9
CXCR3
CXCL11
CXCR7
CXCL12
CXCR4
CXCL13
BLR1
CXCL16
CXCR6
CXCL15
CXCL4
CXCL14
?
C subfamily
XCL1
XCL2
XCR1
CX3C subfamily
CX3CL1
CX3CR1
CC subfamily
CCL1
CCR8
CCL20
CCL25
CCR9
CCL17
CCR4
CCL22
CCL19
CCL21
CCR7
CCR2
CCL12
CCR5
CCL13
CCL7
CCL5
CCR1
CCL14
CCL16
CCL23
CCL15
CCR3
CCL8
CCL11
CCL24
CCL26
CCL28
CCL27
GPR2
CCL6
CCL9
CCL18
Hematopoietins
gp130 (IL6ST) shared
IL6
IL6ST
gp130
IL11RA
human
OSM
LIFR
human / murine
OSMR
CNTFR
CNTF
BSF3
CTF1
CSF3
CSF3R
LEP
LEPR
IL13RA1 shared
IL4
IL4R
IL13RA1
IL13
IL12RB1 shared
IL12RB2
IL23A
IL23R
IL-3RB (CSF2RB) shared
CSF2RA
IL3
IL3RA
IL5
IL5RA
IL2RG shared
IL2RA
IL2RB
IL2RG
IL2
IL7
IL7R
IL9
IL15
IL15RA
IL21
IL21R
TSLP
TSLPR
Single chain
EPO
EPOR
GH1
GH2
GHR
PRL
PRLR
TPO
MPL
α
β
γ
PDGF Family
Receptor tyrosine kinase
PDGFC
PDGFA
PDGFB
PDGFD
PDGFRA
PDGFRB
VEGFB
VEGFA
VEGFE
FLT1
KDR
FLT4
HGF
MET
EGF
EGFR
CSF1
CSF1R
KITLG
KIT
FLT3LG
FLT3
Interferon family
IFNA
IFNB1
IFNW1
IFNK
IFNT1
IFNAR1
IFNAR2
IFNG
IFNGR1
IFNGR2
IL-10 family
IL10
IL10RA
IL10RB
IL20
IL24
IL22RA1
IL22
IL22RA2
IL28A
IL28B
IL29
IL28RA
IL26
TNF Family
TNFR
TNFSF15
SF10A
SF10B
SF10C
SF10D
TNFSF10
SF11B
TNFSF11
SF11A
SF25
TNFSF12
SF12A
SF21
NGFR
TNF
SF1B
SF1A
LTA
LTB
LTBR
TNFSF14
SF14
SF6B
FASLG
FAS
CD40LG
CD40
TNFSF7
SF7
SF8
TNFSF9
SF9
TNFSF4
SF4
TNFSF18
SF18
SF17
TNFSF13
SF13B
TNFSF13B
EDA-A1
EDA
EDA-A2
SF19
TGFB family
TGFB1
TGFB2
TGFB3
TGFBR2
TGFBR1
Type II
Type I
Type I?
INHBA
INHBB
INHBC
INHBE
ACVR2
ACVR1
ACVR1B
ACVR2B
AMH
AMHR2
ACVR1?
BMP2
BMPR2
BMPR1A
BMP7
GDF5
IL-17 family
IL17A
IL17R
IL17B
IL17E
IL17RB
IL-1 family
IL1R1
IL1RAP
IL1B
IL1R2
No signal transduction
IL18
IL18R1
Data on KEGG graph
Rendered by Pathview

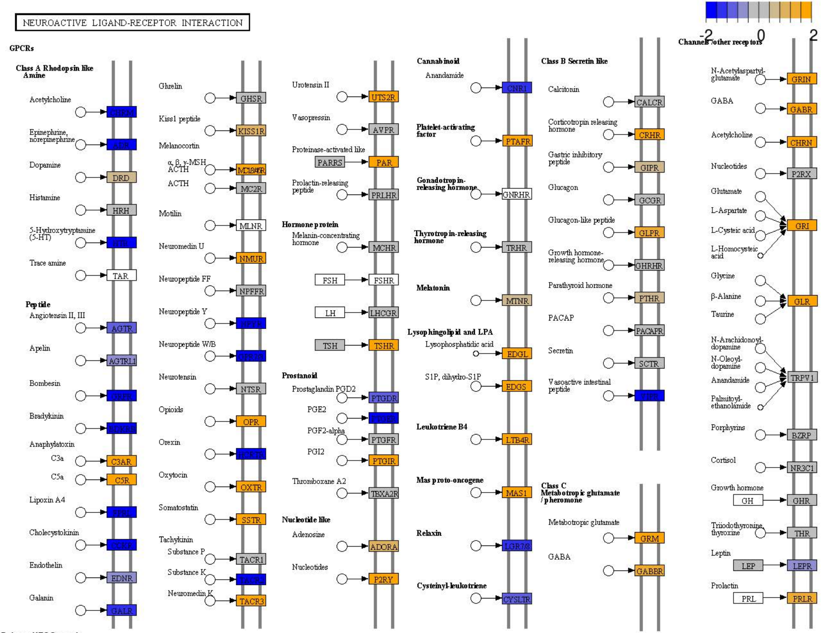

Data on KEGG graph
Rendered by Pathview

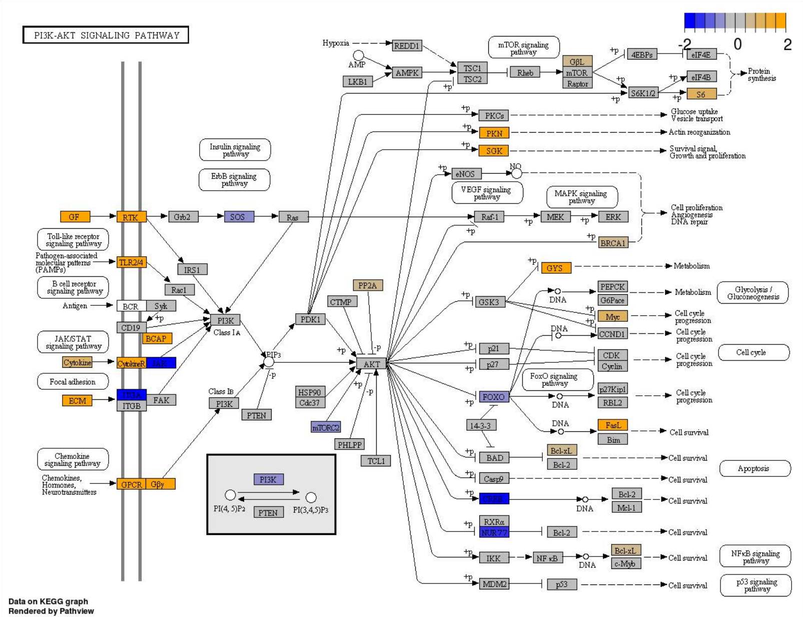
PI3K-AKT SIGNALING PATHWAY
-2
0
2
Hypoxia
REDD1
AMP
AMPK
LKB1
TSC1
TSC2
mTOR signaling pathway
Rheb
GβL
mTOR
Raptor
4EBPs
eIF4E
eIF4B
S6K1/2
S6
Protein synthesis
PKCs
Glucose uptake
Vesicle transport
PKN
Actin reorganization
SGK
Survival signal, Growth and proliferation
Insulin signaling pathway
ErbB signaling pathway
eNOS
NO
VEGF signaling pathway
MAPK signaling pathway
GF
RTK
Grb2
SOS
Ras
Raf-1
MEK
ERK
Cell proliferation
Angiogenesis
DNA repair
BRCA1
Toll-like receptor signaling pathway
Pathogen-associated molecular patterns (PAMPs)
TLR2/4
IRS1
Rac1
B cell receptor signaling pathway
Antigen
BCR
Syk
CD19
BCAP
PI3K
Class IA
JAK/STAT signaling pathway
Cytokine
CytokineR
JAK
Focal adhesion
ECM
ITGA
ITGB
FAK
Chemokine signaling pathway
Chemokines, Hormones, Neurotransmitters
GPCR
Gβγ
Class IB
PI3K
PIP3
PTEN
PDK1
CTMP
PP2A
AKT
HSP90
Cdc37
mTORC2
PHLPP
TCL1
PI3K
PI(4, 5)P2
PI(3,4,5)P3
PTEN
GYS
Metabolism
GSK3
DNA
PEPCK
G6Pace
Metabolism
Glycolysis / Gluconeogenesis
Myc
Cell cycle progression
CCND1
Cell cycle progression
p21
p27
CDK
Cyclin
Cell cycle progression
Cell cycle
FoxO signaling pathway
FOXO
p27Kip1
RBL2
Cell cycle progression
14-3-3
FasL
Bim
Cell survival
BAD
Bcl-xL
Bcl-2
Cell survival
Apoptosis
Casp9
Cell survival
CREB
Bcl-2
Mcl-1
Cell survival
RXRα
NUR77
Bcl-2
Cell survival
IKK
NF κB
Bcl-xL
c-Myb
Cell survival
NFκB signaling pathway
MDM2
p53
Cell survival
p53 signaling pathway
Data on KEGG graph
Rendered by Pathview

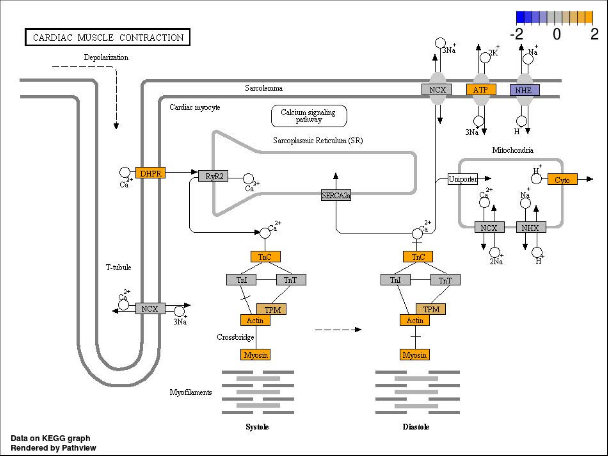

CARDIAC MUSCLE CONTRACTION
-2
0
2
Depolarization
Sarcolemma
Cardiac myocyte
Calcium signaling pathway
Sarcoplasmic Reticulum (SR)
NCX
ATP
NHE
3Na+
2K+
Na+
3Na+
H+
Mitochondria
DHPR
RyR2
Ca2+
SERCA2a
Uniporter
Cyto
NCX
NHX
2Na
TnC
TnI
TnT
TPM
Actin
Myosin
Crossbridge
T-tubule
Myofilaments
Systole
Diastole
Data on KEGG graph
Rendered by Pathview

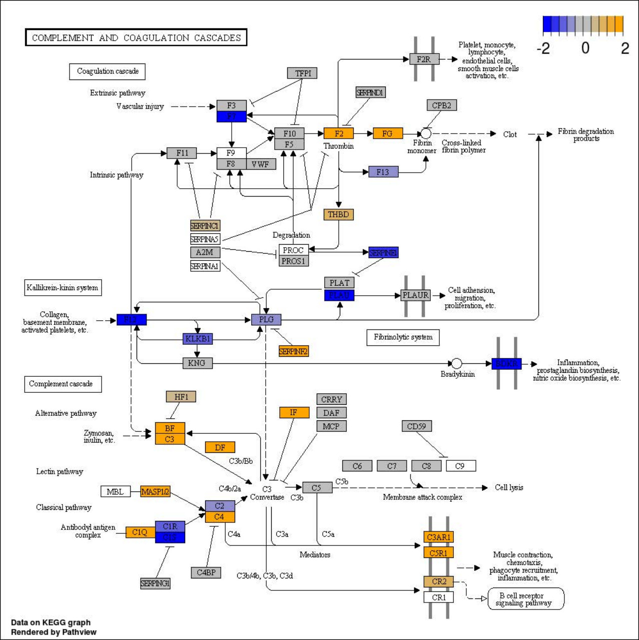
COMPLEMENT AND COAGULATION CASCADES
-2
0
2
Coagulation cascade
Extrinsic pathway
Vascular injury
Intrinsic pathway
TFPI
F3
F7
F10
F5
F2
Thrombin
FG
SERPIND1
F2R
Platelet, monocyte, lymphocyte, endothelial cells, smooth muscle cells activation, etc.
CPB2
Fibrin monomer
Cross-linked fibrin polymer
Clot
Fibrin degradation products
F11
F9
F8
VWF
F13
THBD
SERPINC1
SERPINA5
A2M
SERPINA1
Degradation
PROC
PROS1
SERPINE1
PLAT
PLAU
PLAUR
Cell adhesion, migration, proliferation, etc.
Kallikrein-kinin system
Collagen, basement membrane, activated platelets, etc.
F12
PLG
KLKB1
SERPINF2
Fibrinolytic system
KNG
Bradykinin
Inflammation, prostaglandin biosynthesis, nitric oxide biosynthesis, etc.
Complement cascade
Alternative pathway
HF1
BF
C3
DF
Zymosan, inulin, etc.
IF
CRRY
DAF
MCP
C3b/Bb
CD59
C6
C7
C8
C9
Lectin pathway
MBL
MASP1/2
C4b/2a
C3 Convertase
C3b
C5
C5b
Membrane attack complex
Cell lysis
Classical pathway
C2
C4
Antibodyl antigen complex
C1Q
C1R
C1S
C4a
C3a
C5a
C3AR1
C5R1
SERPING1
C4BP
Mediators
Muscle contraction, chemotaxis, phagocyte recruitment, inflammation, etc.
C3b/4b, C3b, C3d
CR2
CR1
B cell receptor signaling pathway
Data on KEGG graph
Rendered by Pathview

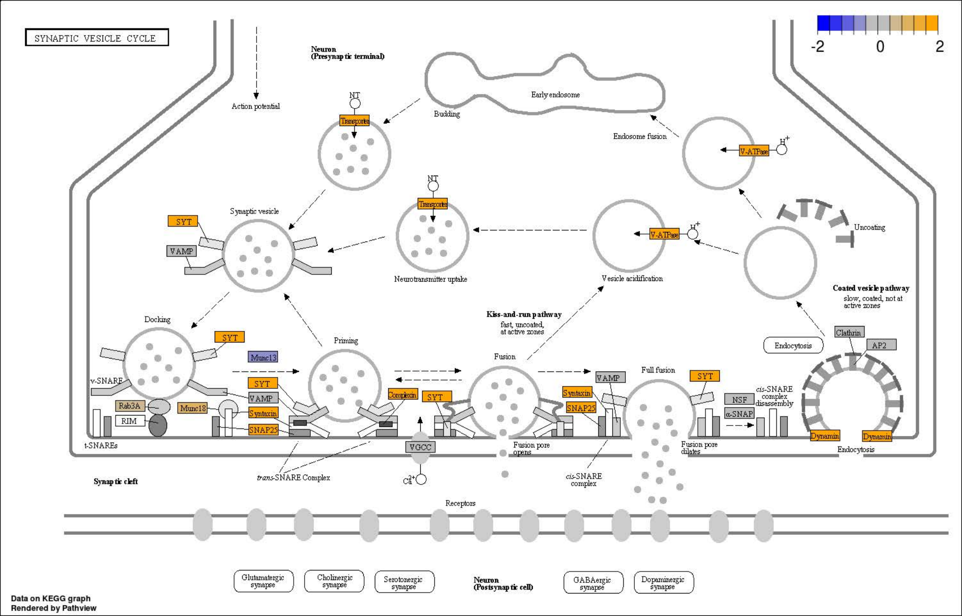
SYNAPTIC VESICLE CYCLE
-2
0
2
Neuron (Presynaptic terminal)
Action potential
NT
Transporter
Budding
Early endosome
Endosome fusion
V-ATPase
H+
Synaptic vesicle
SYT
VAMP
Neurotransmitter uptake
Vesicle acidification
Uncoating
Coated vesicle pathway
slow, coated, not at active zones
Kiss-and-run pathway
fast, uncoated, at active zones
Docking
Priming
Fusion
Full fusion
Munc13
v-SNARE
Rab3A
RIM
Munc18
Syntaxin
SNAP25
Complexin
VGCC
Ca2+
t-SNAREs
trans-SNARE Complex
Fusion pore opens
cis-SNARE complex
Fusion pore dilates
NSF
α-SNAP
cis-SNARE complex disassembly
Clathrin
AP2
Endocytosis
Dynamin
Synaptic cleft
Receptors
Glutamatergic synapse
Cholinergic synapse
Serotonergic synapse
Neuron (Postsynaptic cell)
GABAergic synapse
Dopaminergic synapse
Data on KEGG graph
Rendered by Pathview

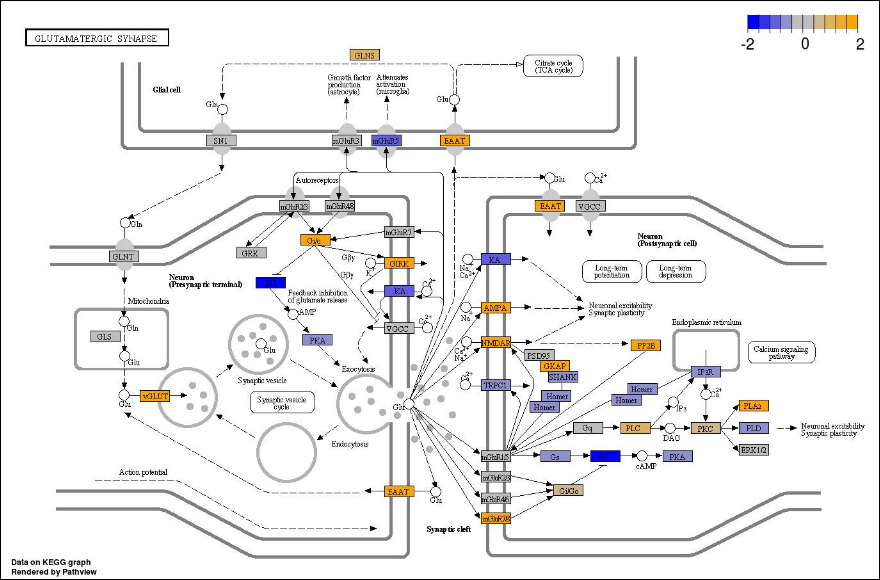

GLUTAMATERGIC SYNAPSE
-2
0
2
GLNS
Citrate cycle (TCA cycle)
Growth factor production (astrocyte)
Attenuates activation (microglia)
Glial cell
Gln
Glu
SN1
mGluR3
mGluR5
EAAT
Autoreceptors
mGluR2/3
mGluR4/8
Gln
GLNT
GRK
Gi/o
mGluR7
Gβγ
GIRK
K+
Neuron (Presynaptic terminal)
AC
Feedback inhibition of glutamate release
KA
Ca2+
AMP
VGCC
Mitochondria
GLS
PKA
Synaptic vesicle
Exocytosis
vGLUT
Synaptic vesicle cycle
Endocytosis
Action potential
EAAT
Synaptic cleft
Neuron (Postsynaptic cell)
VGCC
Long-term potentiation
Long-term depression
Na+
KA
AMPA
NMDAR
Neuronal excitability
Synaptic plasticity
PP2B
PSD95
GKAP
SHANK
Endoplasmic reticulum
Calcium signaling pathway
IP3R
TRPC1
Homer
IP3
Gq
PLC
DAG
PKC
PLA2
PLD
ERK1/2
mGluR1/5
Gs
AC
cAMP
PKA
mGluR2/3
mGluR4/6
mGluR7/8
Gi/Go
Data on KEGG graph
Rendered by Pathview

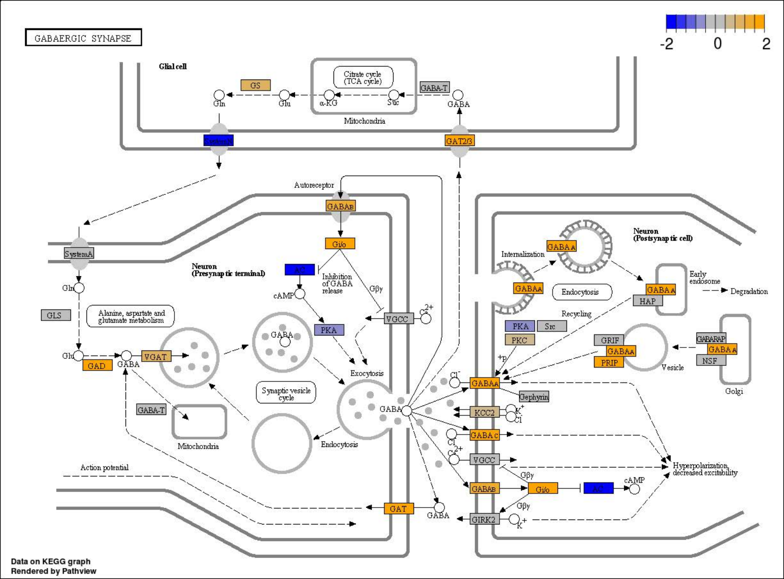

GABAERGIC SYNAPSE
-2
0
2
Glial cell
GS
GABA-T
Citrate cycle (TCA cycle)
Gln
Glu
α-KG
Suc
GABA
Mitochondria
GAT2/3
Autoreceptor
GABAB
Gi/o
AC
SystemA
Neuron (Presynaptic terminal)
Inhibition of GABA release
cAMP
Gβγ
GLS
Alanine, aspartate and glutamate metabolism
PKA
VGCC
GAD
VGAT
Exocytosis
Synaptic vesicle cycle
GABA-T
Mitochondria
Endocytosis
Action potential
GAT
Neuron (Postsynaptic cell)
Internalization
Endocytosis
Early endosome
Degradation
HAP
Recycling
PKC
Src
GRIF
PRIP
GABARAP
NSF
Vesicle
Golgi
GABAA
Gephyrin
KCC2
GABAC
GIRK2
Hyperpolarization, decreased excitability
Data on KEGG graph
Rendered by Pathview

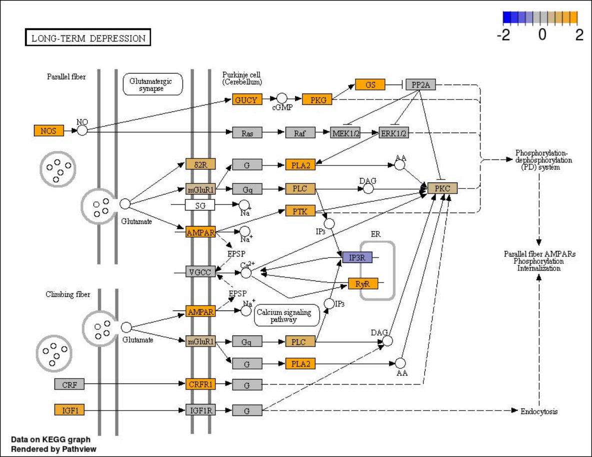
-2
0
2
LONG-TERM DEPRESSION
Parallel fiber
Glutamatergic synapse
Purkinje cell (Cerebellum)
GUCY
cGMP
PKG
GS
PP2A
NOS
NO
Ras
Raf
MEK1/2
ERK1/2
Phosphorylation-dephosphorylation (PD) system
δ2R
G
PLA2
AA
mGluR1
Gq
PLC
DAG
PKC
SG
Na+
Glutamate
PTK
AMPAR
IP3
ER
EPSP
Ca2+
IP3R
VGCC
RyR
Parallel fiber AMPARs Phosphorylation Internalization
Climbing fiber
EPSP
AMPAR
Na+
IP3
Calcium signaling pathway
Glutamate
mGluR1
Gq
PLC
DAG
G
PLA2
AA
CRF
CRFR1
G
IGF1
IGF1R
G
Endocytosis
Data on KEGG graph
Rendered by Pathview

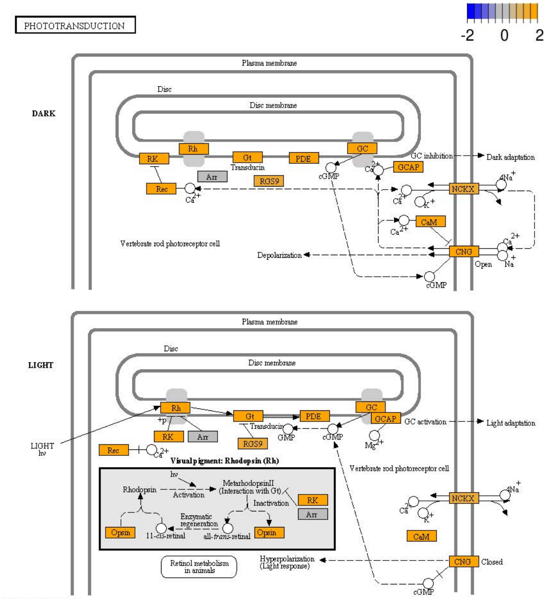

Data on KEGG graph
Rendered by Pathview

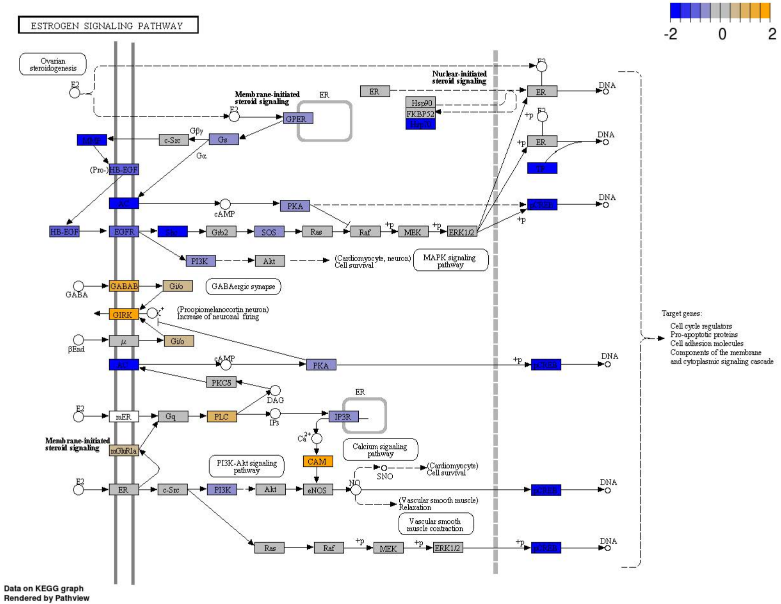

ESTROGEN SIGNALING PATHWAY
-2
0
2
Ovarian steroidogenesis
E2
Membrane-initiated steroid signaling
ER
GPER
Gs
Gβγ
Gα
c-Src
MMP
(Pro-)HB-EGF
AC
cAMP
PKA
HB-EGF
EGFR
Shc
Grb2
SOS
Ras
Raf
MEK
ERK1/2
+p
PI3K
Akt
(Cardiomyocyte, neuron) Cell survival
MAPK signaling pathway
Nuclear-initiated steroid signaling
Hsp90
FKBP52
Hsp70
DNA
TF
pCREB
GABA
GABAB
Gi/o
GABAergic synapse
GIRK
K+
(Proopiomelanocortin neuron) Increase of neuronal firing
βEnd
μ
PKCδ
DAG
mER
Gq
PLC
IP3
IP3R
Ca2+
CAM
Calcium signaling pathway
mGluR1a
PI3K-Akt signaling pathway
eNOS
NO
SNO
(Cardiomyocyte) Cell survival
(Vascular smooth muscle) Relaxation
Vascular smooth muscle contraction
Target genes:
Cell cycle regulators
Pro-apoptotic proteins
Cell adhesion molecules
Components of the membrane and cytoplasmic signaling cascade
Data on KEGG graph
Rendered by Pathview

S7B Fig

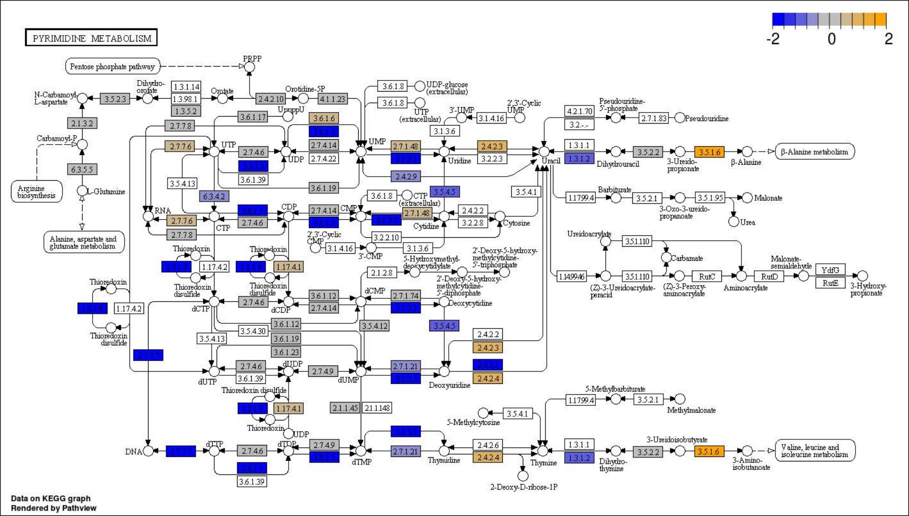
PYRIMIDINE METABOLISM
-2
0
2
Pentose phosphate pathway
PRPP
N-Carbamoyl-L-aspartate
3.5.2.3
Dihydro-orotate
1.3.1.14
1.3.98.1
1.3.5.2
Orotate
2.4.2.10
Orotidine-5P
4.1.1.23
2.1.3.2
Carbamoyl-P
6.3.5.5
Arginine biosynthesis
L-Glutamine
Alanine, aspartate and glutamate metabolism
2.7.7.8
2.7.7.6
3.6.1.17
UppppU
3.6.1.6
UTP
2.7.4.6
UDP
2.7.4.14
2.7.4.22
3.6.1.39
3.5.4.13
3.6.1.19
6.3.4.2
UMP
3.6.1.8
UDP-glucose (extracellular)
UTP (extracellular)
3'-UMP
2',3'-Cyclic UMP
3.1.4.16
3.1.3.6
2.7.1.48
Uridine
2.4.2.3
3.2.2.3
2.4.2.9
Uracil
4.2.1.70
3.2.-.-
Pseudouridine-5'-phosphate
2.7.1.83
Pseudouridine
1.3.1.1
1.3.1.2
Dihydrouracil
3.5.2.2
3-Ureido-propionate
3.5.1.6
β-Alanine
β-Alanine metabolism
RNA
CTP
CDP
CMP
2.7.7.6
2.7.7.8
CTP (extracellular)
3.5.4.5
3.5.4.1
2.4.2.2
3.2.2.8
Cytidine
Cytosine
3.2.2.10
2',3'-Cyclic CMP
3'-CMP
1.17.99.4
Barbiturate
3.5.2.1
3-Oxo-3-ureido-propanoate
3.5.1.95
Malonate
Urea
Thioredoxin
Thioredoxin disulfide
1.17.4.2
1.17.4.1
5-Hydroxymethyl-deoxycytidine
2'-Deoxy-5-hydroxy-methylcytidine-5'-triphosphate
2'-Deoxy-5-hydroxy-methylcytidine-5'-diphosphate
2.1.2.8
dCMP
2.7.1.74
Deoxycytidine
dCTP
dCDP
3.6.1.12
3.5.4.30
3.5.4.12
3.6.1.23
Ureidoacrylate
3.5.1.110
Carbamate
1.14.99.46
(Z)-3-Ureidoacrylate-peracid
(Z)-3-Peroxy-aminoacrylate
RutC
Aminoacrylate
RutD
Malonate-semialdehyde
YdfG
RutE
3-Hydroxy-propionate
dUTP
dUDP
2.7.4.9
dUMP
2.7.1.21
Deoxyuridine
2.4.2.4
2.1.1.45
2.1.1.148
5-Methylbarbiturate
Methylmalonate
5-Methylcytosine
Thymidine
2.4.2.6
Thymine
3-Ureidoisobutyrate
Dihydro-thymine
3-Amino-isobutanoate
Valine, leucine and isoleucine metabolism
2-Deoxy-D-ribose-1P
DNA
dTTP
dTDP
dTMP
Data on KEGG graph
Rendered by Pathview

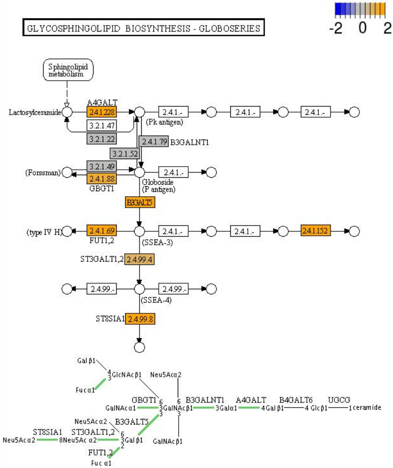

Data on KEGG graph
Rendered by Pathview

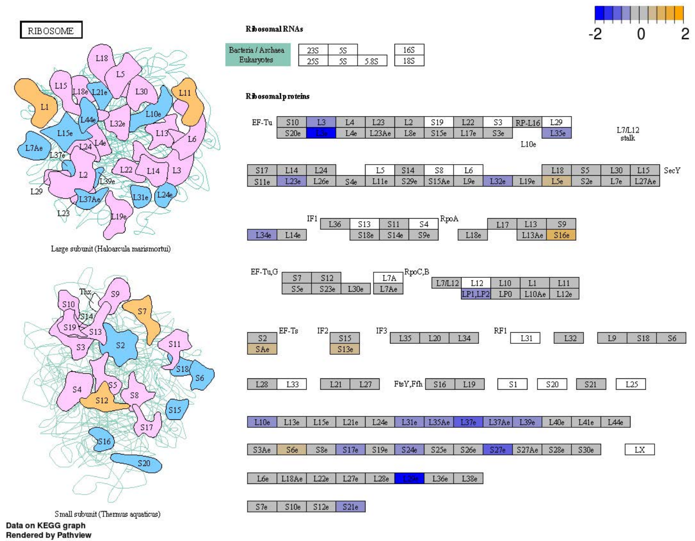
RIBOSOME
Ribosomal RNAs
Bacteria / Archaea
Eukaryotes
23S
5S
16S
25S
5S
5.8S
18S
Ribosomal proteins
-2
0
2
EF-Tu
S10 L3 L4 L23 L2 S19 L22 S3 RP-L16 L29
S20e L4e L23Ae L8e S15e L17e S3e L35e
L10e
L7/L12 stalk
S17 L14 L24 L5 S14 S8 L6 L18 S5 L30 L15
S11e L23e L26e S4e L11e S29e S15Ae L9e L32e L19e L5e S2e L7e L27Ae
SecY
IF1
L36 S13 S11 S4
RpoA
L17 L13 S9
L34e L14e S18e S14e S9e L18e L13Ae S16e
EF-Tu,G
S7 S12
S5e S23e L30e
L7A
L7Ae
RpoC,B
L7/L12 L12 L10 L1 L11
LP1,LP2 LP0 L10Ae L12e
S2
SAe
EF-Ts
IF2
S15
S13e
IF3
L35 L20 L34
RF1
L31 L32 L9 S18 S6
L28 L33 L21 L27
FtsY,Ffh
S16 L19 S1 S20 S21 L25
L10e L13e L15e L21e L24e L31e L35Ae L37e L37Ae L39e L40e L41e L44e
S3Ae S6e S8e S17e S19e S24e S25e S26e S27e S27Ae S28e S30e
LX
L6e L18Ae L22e L27e L28e L36e L38e
S7e S10e S12e S21e
L18 L5 L15 L18e L21e L30 L1 L11 L10e L44e L32e L15e L13 L6 L7Ae L37e L24 L4e L2 L22 L14 L3 L39e L29 L37Ae L31e L24e L23 L19e
Large subunit (Haloarcula marismortui)
Thx S9 S10 S14 S7 S19 S13 S2 S3 S11 S18 S6 S4 S5 S12 S8 S15 S17 S16 S20
Small subunit (Thermus aquaticus)
Data on KEGG graph
Rendered by Pathview

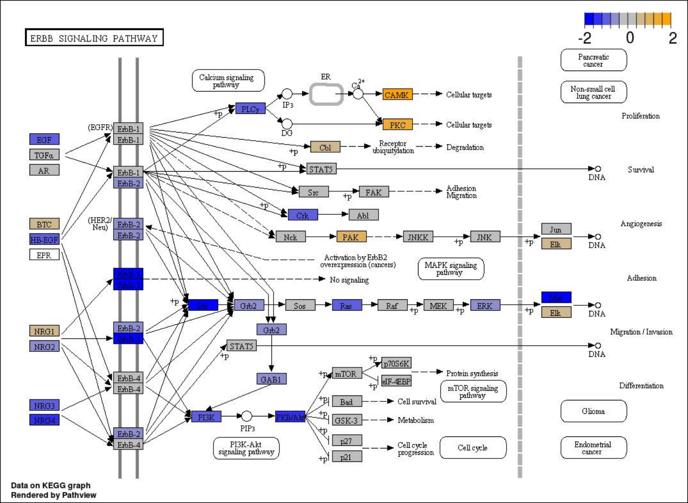
ERBB SIGNALING PATHWAY
-2
0
2
Pancreatic cancer
Non-small cell lung cancer
Proliferation
Survival
Angiogenesis
Adhesion
Migration / Invasion
Differentiation
Glioma
Endometrial cancer
Calcium signaling pathway
ER
IP3
DG
PLCγ
CAMK
PKC
Cellular targets
Cellular targets
Cbl
Receptor ubiquitylation
Degradation
STAT5
DNA
Src
FAK
Adhesion Migration
Crk
Abl
Nck
PAK
JNKK
JNK
Jun
Elk
DNA
Activation by ErbB2 overexpression (cancers)
MAPK signaling pathway
No signaling
Grb2
Sos
Ras
Raf
MEK
ERK
Elk
DNA
Grb2
STAT5
DNA
GAB1
mTOR
p70S6K
eIF-4EBP
Protein synthesis
mTOR signaling pathway
Bad
Cell survival
GSK-3
Metabolism
p27
p21
Cell cycle progression
Cell cycle
PI3K
PIP3
PKB/Akt
PI3K-Akt signaling pathway
EGF
TGFα
AR
BTC
HB-EGF
EPR
NRG1
NRG2
NRG3
NRG4
(EGFR)
ErbB-1
ErbB-2
(HER2/ Neu)
ErbB-4
Data on KEGG graph
Rendered by Pathview

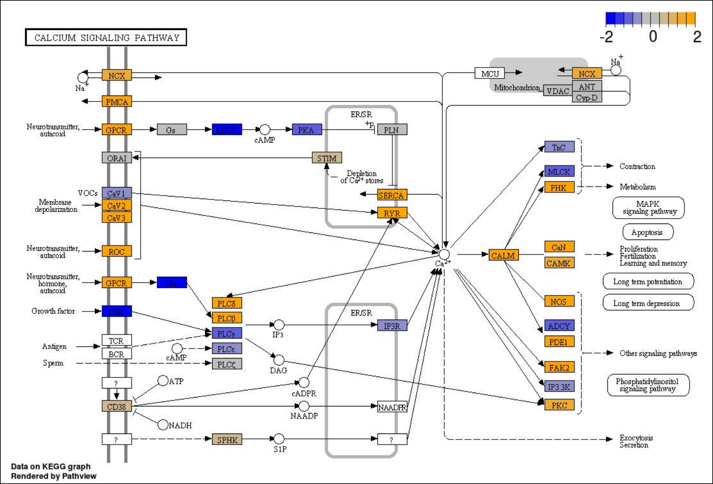
CALCIUM SIGNALING PATHWAY
-2
0
2
Na+
NCX
PMCA
Neurotransmitter, autacoid
GPCR
Gs
ADCY
cAMP
PKA
ER/SR
+p
PLN
MCU
Mitochondrion
VDAC
ANT
Cyp-D
NCX
Na+
ORAI
STIM
Depletion of Ca2+ stores
VOCs
CaV1
Membrane depolarization
CaV2
CaV3
SERCA
RYR
Neurotransmitter, autacoid
ROC
Ca2+
CALM
TnC
MLCK
PHK
Contraction
Metabolism
MAPK signaling pathway
Apoptosis
CaN
CAMK
Proliferation
Fertilization
Learning and memory
Long term potentiation
Long term depression
Neurotransmitter, hormone, autacoid
GPCR
Gq
Growth factor
PTK
PLCδ
PLCβ
PLCγ
PLCε
PLCζ
IP3
IP3R
ER/SR
Antigen
TCR
BCR
cAMP
Sperm
DAG
?
ATP
cADPR
CD38
NAADP
NAADPR
NADH
?
SPHK
S1P
?
NOS
ADCY
PDE1
FAK2
IP3 3K
PKC
Other signaling pathways
Phosphatidylinositol signaling pathway
Exocytosis
Secretion
Data on KEGG graph
Rendered by Pathview

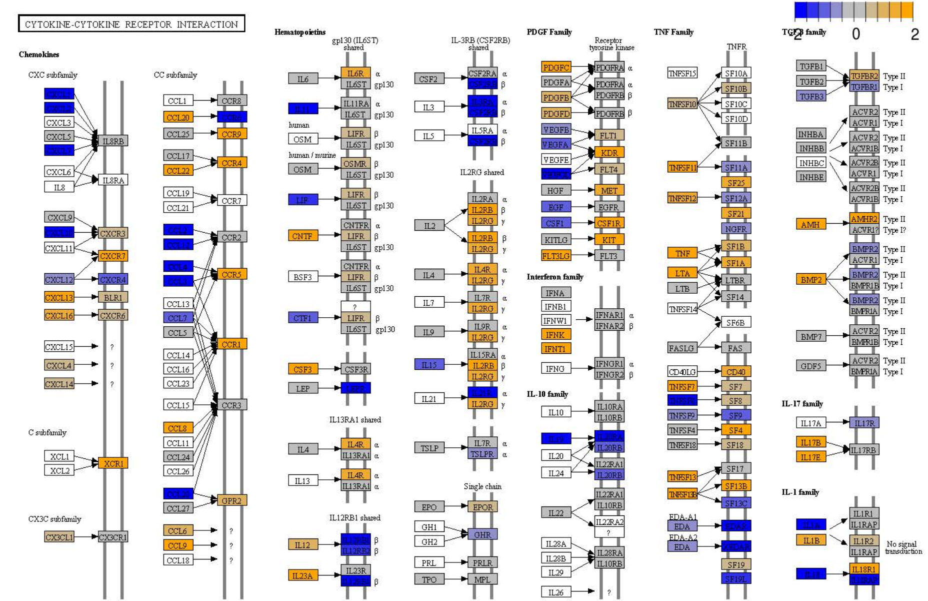

Data on KEGG graph
Rendered by Pathview

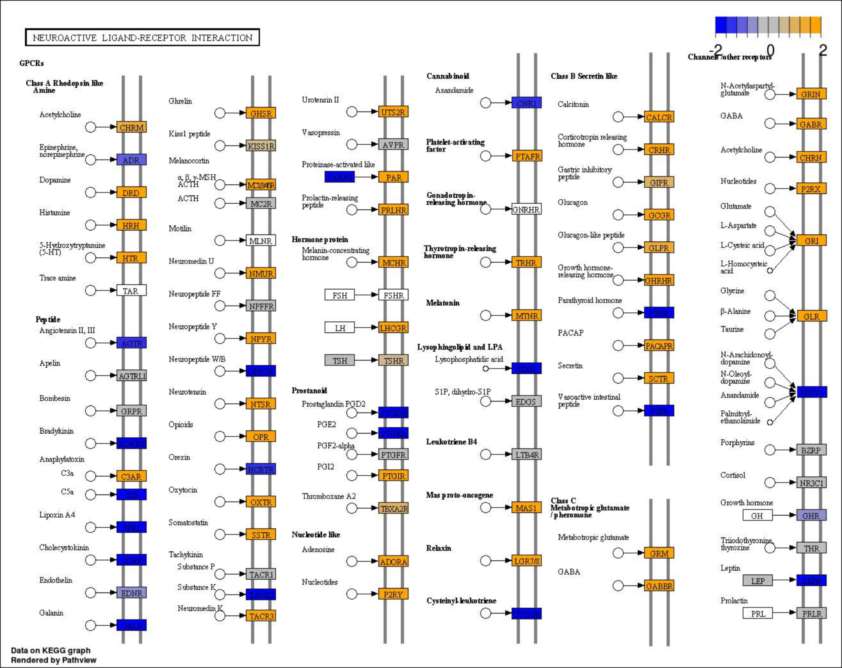
NEUROACTIVE LIGAND-RECEPTOR INTERACTION
-2
0
2
GPCRs
Class A Rhodopsin like
Amine
Acetylcholine
CHRM
Epinephrine, norepinephrine
ADR
Dopamine
DRD
Histamine
HRH
5-Hydroxytryptamine (5-HT)
HTR
Trace amine
TAR
Peptide
Angiotensin II, III
AGTR
Apelin
AGTRL1
Bombesin
GRPR
Bradykinin
Anaphylatoxin
C3a
C3AR
C5a
Lipoxin A4
Cholecystokinin
Endothelin
EDNR
Galanin
Ghrelin
GHSR
Kiss1 peptide
KISS1R
Melanocortin
α, β, γ-MSH
ACTH
ACTH
MC2R
Motilin
MLNR
Neuromedin U
NMUR
Neuropeptide FF
NPFFR
Neuropeptide Y
NPYR
Neuropeptide W/B
Neurotensin
NTSR
Opioids
OPR
Orexin
HCRTR
Oxytocin
OXTR
Somatostatin
SSTR
Tachykinin
Substance P
TACR1
Substance K
Neuromedin K
TACR3
Urotensin II
UTS2R
Vasopressin
AVPR
Proteinase-activated like
PAR
Prolactin-releasing peptide
PRLHR
Hormone protein
Melanin-concentrating hormone
MCHR
FSH
FSHR
LH
LHCGR
TSH
TSHR
Prostanoid
Prostaglandin PGD2
PGE2
PGF2-alpha
PTGFR
PGI2
PTGIR
Thromboxane A2
TBXA2R
Nucleotide like
Adenosine
ADORA
Nucleotides
P2RY
Cannabinoid
Anandamide
CNR1
Platelet-activating factor
PTAFR
Gonadotropin-releasing hormone
GNRHR
Thyrotropin-releasing hormone
TRHR
Melatonin
MTNR
Lysophingolipid and LPA
Lysophosphatidic acid
S1P, dihydro-S1P
EDG5
Leukotriene B4
LTB4R
Mas proto-oncogene
MAS1
Relaxin
LGR7/8
Cysteinyl-leukotriene
Class B Secretin like
Calcitonin
CALCR
Corticotropin releasing hormone
CRHR
Gastric inhibitory peptide
GIPR
Glucagon
GCGR
Glucagon-like peptide
GLPR
Growth hormone-releasing hormone
GHRHR
Parathyroid hormone
PACAP
PACAPR
Secretin
SCTR
Vasoactive intestinal peptide
Class C Metabotropic glutamate /pheromone
Metabotropic glutamate
GRM
GABA
GABBR
Channels/other receptors
N-Acetylaspartyl-glutamate
GRIN
GABA
GABR
Acetylcholine
CHRN
Nucleotides
P2RX
Glutamate
L-Aspartate
L-Cysteic acid
L-Homocysteic acid
GRI
Glycine
β-Alanine
Taurine
GLR
N-Arachidonoyl-dopamine
N-Oleoyl-dopamine
Anandamide
Palmitoyl-ethanolamide
Porphyrins
BZRP
Cortisol
NR3C1
Growth hormone
GH
GHR
Triiodothyronine, thyroxine
THR
Leptin
LEP
Prolactin
PRL
PRLR
Data on KEGG graph
Rendered by Pathview

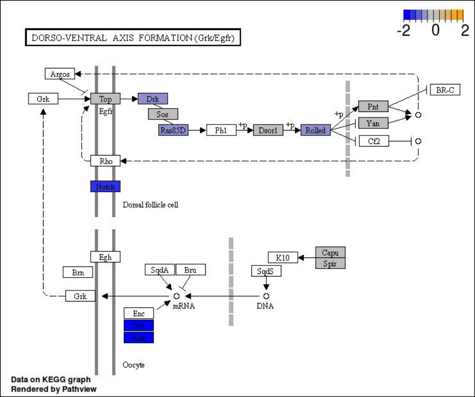

-2 0 2
DORSO-VENTRAL AXIS FORMATION (Grk/Egfr)
Argos
Grk
Top
Egfr
Drk
Sos
Ras85D
Ph1
+p
Dsor1
+p
Rolled
+p
Pnt
Yan
Cf2
BR-C
Rho
Notch
Dorsal follicle cell
Egh
Brn
Grk
SqdA
Bru
mRNA
K10
Capu
Spir
SqdS
DNA
Enc
Orb
Aub
Oocyte
Data on KEGG graph
Rendered by Pathview

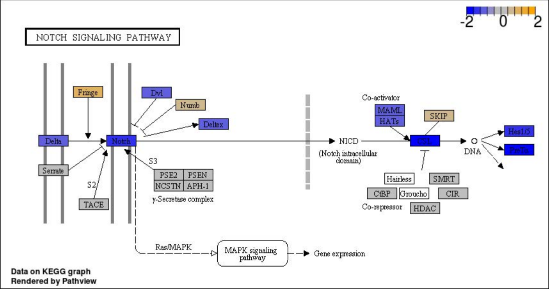

-2
0
2
NOTCH SIGNALING PATHWAY
Fringe
Dvl
Numb
Deltex
Delta
Notch
Serrate
S2
TACE
S3
PSE2
PSEN
NCSTN
APH-1
γ-Secretase complex
NICD
(Notch intracellular domain)
Co-activator
MAML
HATs
SKIP
CSL
DNA
Hes1/5
PreTa
Hairless
SMRT
CtBP
Groucho
CIR
Co-repressor
HDAC
Ras/MAPK
MAPK signaling pathway
Gene expression
Data on KEGG graph
Rendered by Pathview

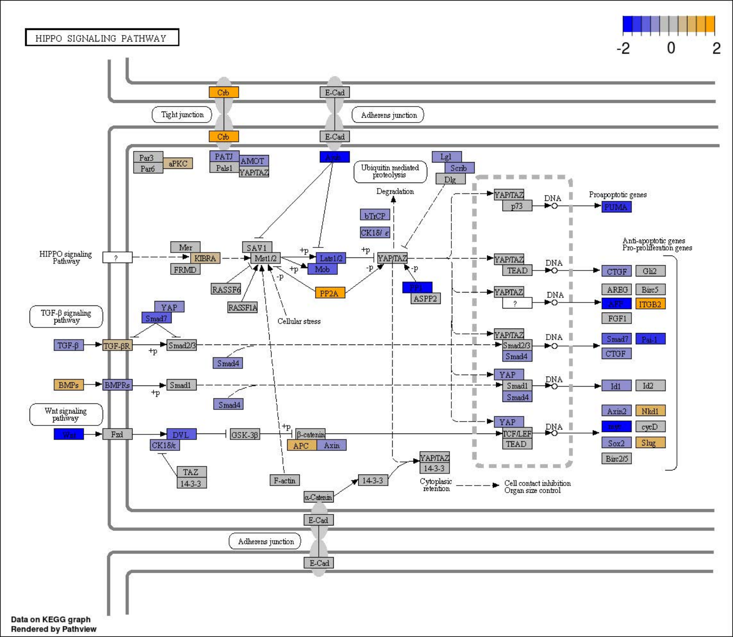

HIPPO SIGNALING PATHWAY
-2
0
2
Crb
E-Cad
Tight junction
Adherens junction
Crb
E-Cad
Par3
Par6
aPKC
PATJ
Pals1
AMOT
YAP/TAZ
Ajub
Ubiquitin mediated proteolysis
Lgl
Scrib
Dlg
Degradation
YAP/TAZ
p73
DNA
Proapoptotic genes
PUMA
bTrCP
CK1δ/ε
Anti-apoptotic genes
Pro-proliferation genes
HIPPO signaling Pathway
?
Mer
KIBRA
FRMD
SAV1
Mst1/2
Lats1/2
Mob
YAP/TAZ
YAP/TAZ
TEAD
DNA
CTGF
Gli2
+p
-p
RASSF6
PP2A
PP1
ASPP2
YAP/TAZ
?
DNA
AREG
Birc5
AFP
ITGB2
FGF1
RASSF1A
Cellular stress
YAP
Smad7
TGF-β signaling pathway
TGF-β
TGF-βR
Smad2/3
YAP/TAZ
Smad2/3
Smad4
DNA
Smad7
Pai-1
CTGF
Smad4
BMPs
BMPRs
Smad1
YAP
Smad1
Smad4
DNA
Id1
Id2
Smad4
Wnt signaling pathway
Wnt
Fzd
DVL
CK1δ/ε
GSK-3β
β-catenin
APC
Axin
YAP
TCF/LEF
TEAD
DNA
Axin2
Nkd1
cycD
Sox2
Slug
Birc2/5
TAZ
14-3-3
F-actin
YAP/TAZ
14-3-3
14-3-3
α-Catenin
Cytoplasic retention
Cell contact inhibition
Organ size control
E-Cad
Adherens junction
E-Cad
Data on KEGG graph
Rendered by Pathview

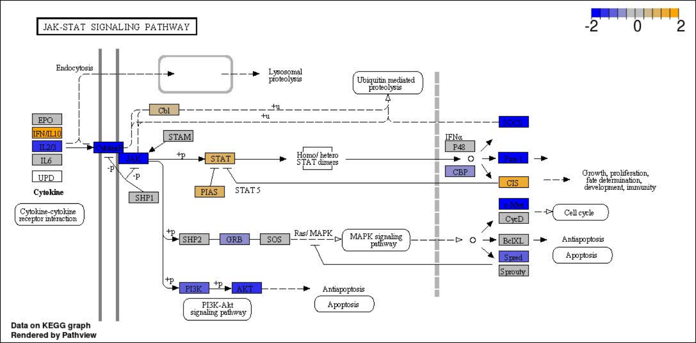
JAK-STAT SIGNALING PATHWAY
-2
0
2
Endocytosis
Lysosomal proteolysis
Ubiquitin mediated proteolysis
Cbl
+u
+u
SOCS
EPO
IFN/IL10
IL2/3
IL6
UPD
Cytokine
Cytokine-cytokine receptor interaction
STAM
JAK
-p
-p
SHP1
+p
STAT
PIAS
STAT 5
Homo/ hetero STAT dimers
IFNα
P48
CBP
CIS
Growth, proliferation, fate determination, development, immunity
CycD
Cell cycle
+p
SHP2
GRB
SOS
Ras/ MAPK
MAPK signaling pathway
BclXL
Antiapoptosis
Apoptosis
Spred
Sprouty
+p
PI3K
+p
AKT
Antiapoptosis
Apoptosis
PI3K-Akt signaling pathway
Data on KEGG graph
Rendered by Pathview

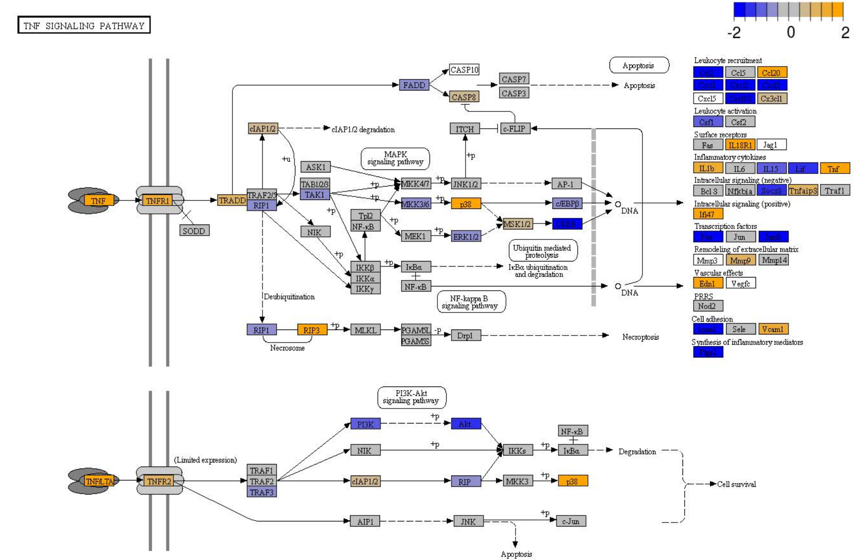

Data on KEGG graph
Rendered by Pathview

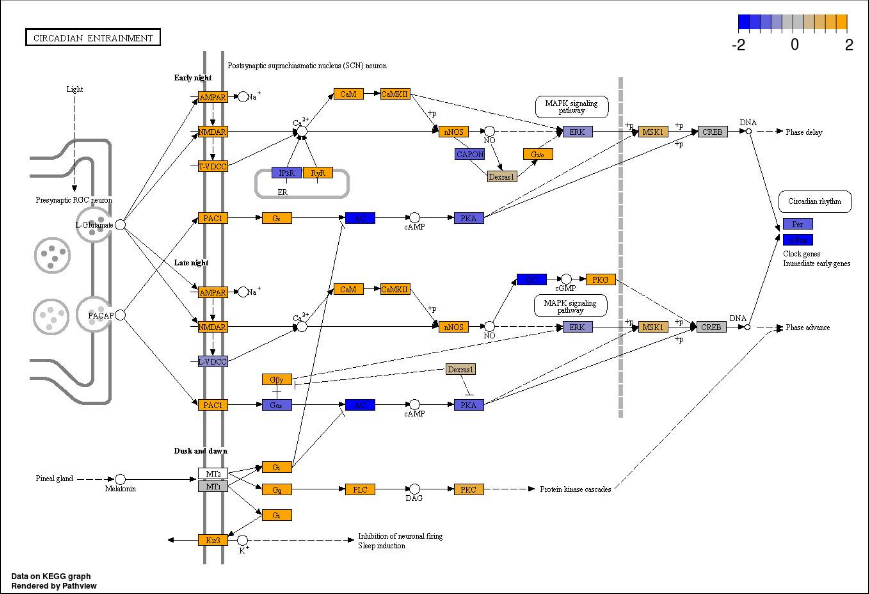
CIRCADIAN ENTRAINMENT
-2
0
2
Light
Presynaptic RGC neuron
L-Glutamate
PACAP
Postsynaptic suprachiasmatic nucleus (SCN) neuron
Early night
Late night
Dusk and dawn
AMPAR
NMDAR
T-VDCC
L-VDCC
PAC1
Na+
Ca2+
CaM
CaMKII
IP3R
RyR
ER
Gs
AC
cAMP
PKA
nNOS
NO
CAPON
Dexras1
Gi/o
MAPK signaling pathway
ERK
MSK1
CREB
DNA
Phase delay
Phase advance
Circadian rhythm
Per
c-Fos
Clock genes
Immediate early genes
GC
cGMP
PKG
Gβγ
Gαs
Pineal gland
Melatonin
MT2
MT1
Gi
Gq
PLC
DAG
PKC
Protein kinase cascades
Kir3
K+
Inhibition of neuronal firing
Sleep induction
Data on KEGG graph
Rendered by Pathview

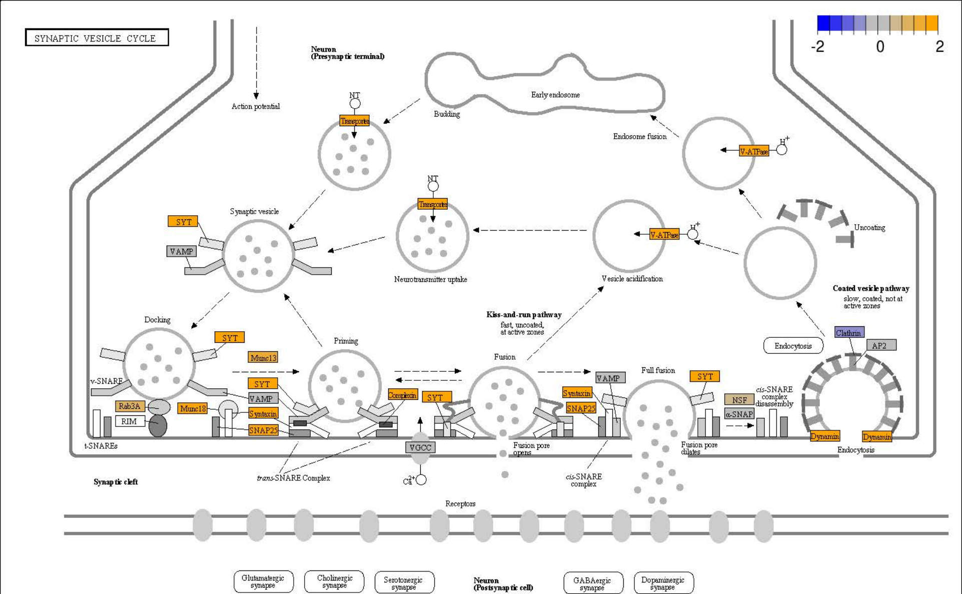

Data on KEGG graph
Rendered by Pathview

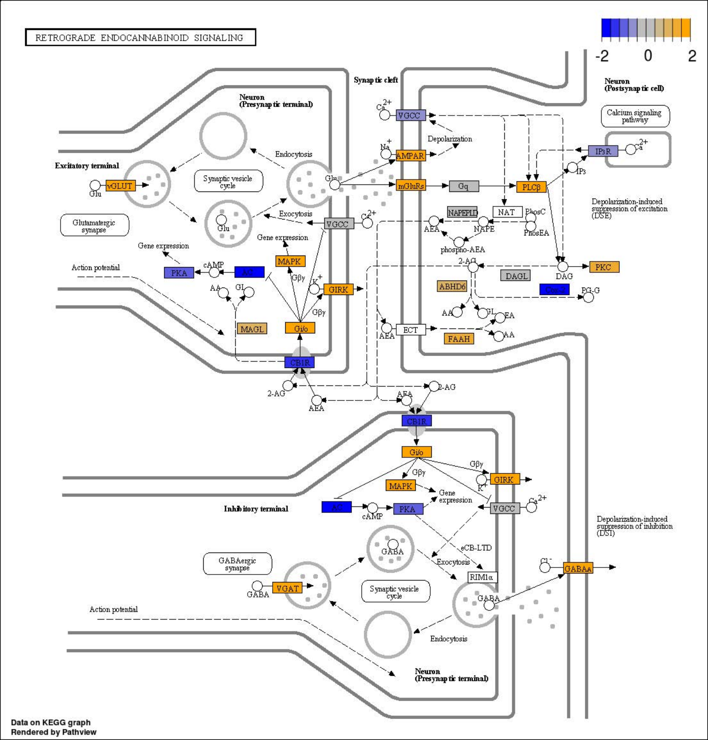
RETROGRADE ENDOCANNABINOID SIGNALING
-2
0
2
Synaptic cleft
Neuron (Presynaptic terminal)
Neuron (Postsynaptic cell)
Calcium signaling pathway
Excitatory terminal
vGLUT
Glu
Synaptic vesicle cycle
Endocytosis
Exocytosis
Glutamatergic synapse
VGCC
AMPAR
mGluRs
Gq
PLCβ
IP3R
IP3
Depolarization
Depolarization-induced suppression of excitation (DSE)
NAPEPLD
NAT
PhosC
PhosEA
AEA
NAPE
phospho-AEA
2-AG
DAG
PKC
DAGL
ABHD6
Cox-2
PG-G
AA
GL
EA
ECT
FAAH
Gene expression
MAPK
PKA
cAMP
AC
GIRK
Gβγ
Gi/o
MAGL
CB1R
Action potential
Inhibitory terminal
GABAergic synapse
VGAT
GABA
RIM1α
eCB-LTD
GABAA
Cl-
Depolarization-induced suppression of inhibition (DSI)
Neuron (Presynaptic terminal)
Data on KEGG graph
Rendered by Pathview

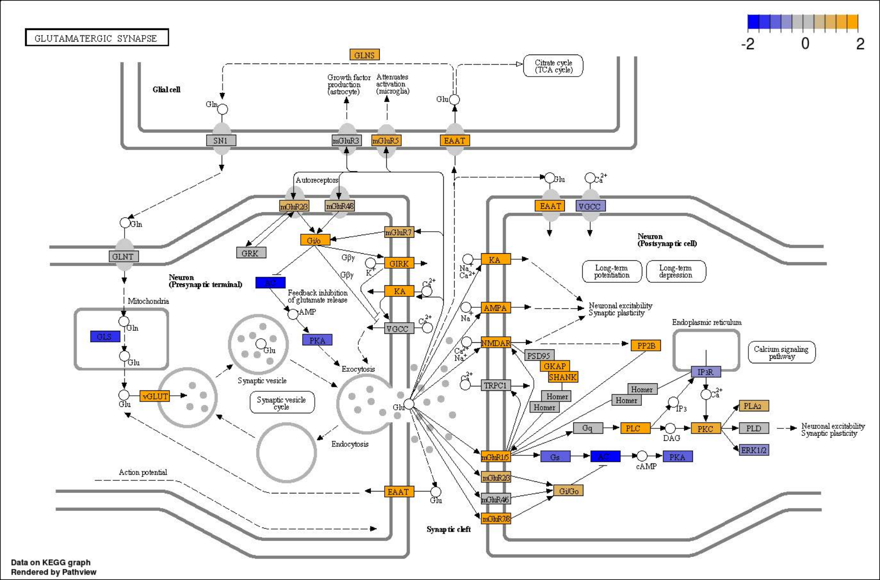
GLUTAMATERGIC SYNAPSE
-2
0
2
GLNS
Glial cell
Growth factor production (astrocyte)
Attenuates activation (microglia)
Citrate cycle (TCA cycle)
Gln
Glu
SN1
mGluR3
mGluR5
EAAT
Autoreceptors
mGluR2/3
mGluR4/8
Gln
GLNT
GRK
Gi/o
mGluR7
Gβγ
GIRK
K+
Gβγ
KA
Ca2+
Neuron (Presynaptic terminal)
AC
Feedback inhibition of glutamate release
AMP
VGCC
Ca2+
Mitochondria
Gln
GLS
Glu
PKA
Synaptic vesicle
Exocytosis
Glu
vGLUT
Synaptic vesicle cycle
Glu
Endocytosis
Action potential
EAAT
Glu
Synaptic cleft
Glu
Ca2+
EAAT
VGCC
Neuron (Postsynaptic cell)
Long-term potentiation
Long-term depression
Na+
Ca2+
KA
AMPA
Na+
Neuronal excitability
Synaptic plasticity
Endoplasmic reticulum
Calcium signaling pathway
Ca2+
Na+
NMDAR
PSD95
GKAP
SHANK
PP2B
TRPC1
Homer
Homer
Homer
Homer
IP3R
Ca2+
IP3
Ca2+
PLA2
Gq
PLC
DAG
PKC
PLD
Neuronal excitability
Synaptic plasticity
ERK1/2
mGluR1/5
Gs
AC
cAMP
PKA
mGluR2/3
Gi/Go
mGluR4/6
mGluR7/8
Data on KEGG graph
Rendered by Pathview

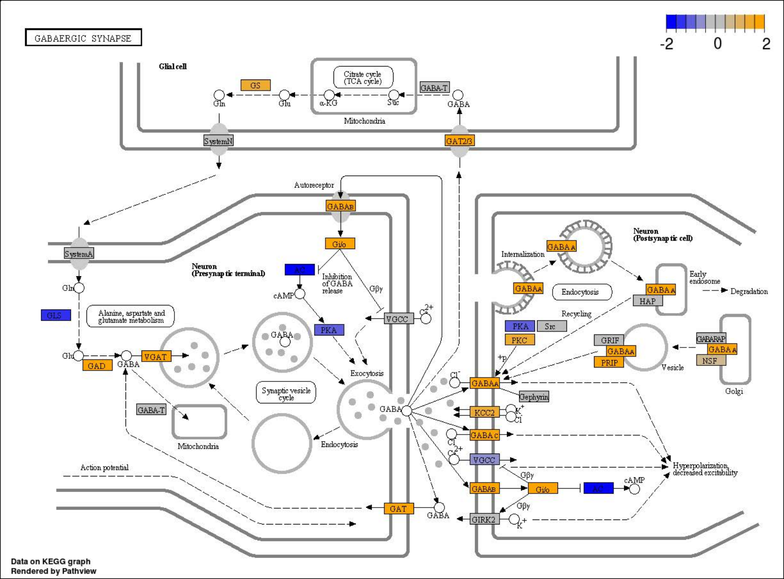

GABAERGIC SYNAPSE
-2
0
2
Glial cell
GS
Gln
Glu
Citrate cycle (TCA cycle)
α-KG
Suc
GABA-T
GABA
Mitochondria
SystemN
GAT2/3
Autoreceptor
GABAB
Gi/o
AC
Inhibition of GABA release
cAMP
Gβγ
SystemA
Neuron (Presynaptic terminal)
Gln
GLS
Alanine, aspartate and glutamate metabolism
PKA
VGCC
Ca^{2+}
GABA
GAD
VGAT
Exocytosis
Synaptic vesicle cycle
GABA-T
Mitochondria
Endocytosis
Action potential
GAT
Neuron (Postsynaptic cell)
Internalization
GABAA
Endocytosis
Recycling
Early endosome
Degradation
HAP
PKA
Src
PKC
+p
GRIF
PRIP
Vesicle
GABARAP
NSF
Golgi
Cl^-
Gephyrin
KCC2
K^+
Cl^-
GABAC
Hyperpolarization, decreased excitability
AC
GIRK2
Data on KEGG graph
Rendered by Pathview

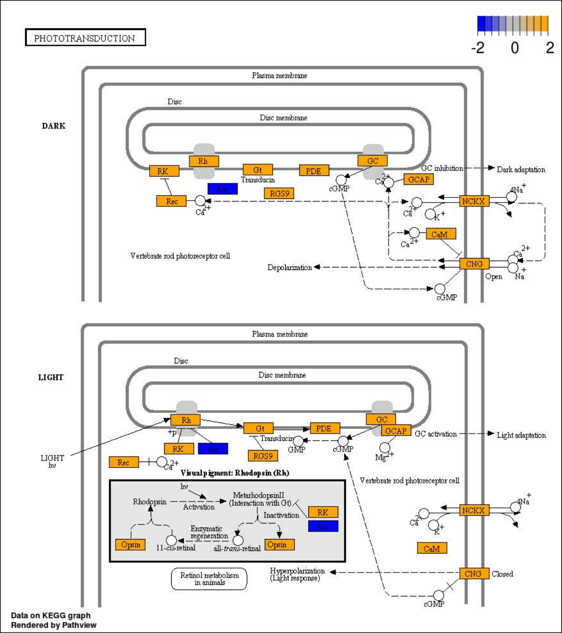
PHOTOTRANSDUCTION
-2
0
2
DARK
Plasma membrane
Disc
Disc membrane
Rh
GC
RK
Gt
Transducin
PDE
GC inhibition
Dark adaptation
GCAP
Arr
RGS9
cGMP
Ca2+
4Na+
Rec
Ca2+
NCKX
K+
CaM
Vertebrate rod photoreceptor cell
Depolarization
CNG
Open
Na
cGMP
LIGHT
Plasma membrane
Disc
Disc membrane
Rh
GC
Gt
PDE
GCAP
GC activation
Light adaptation
+p
Transducin
GMP
cGMP
Mg2+
RK
Arr
RGS9
LIGHT
hv
Rec
Ca2+
Visual pigment: Rhodopsin (Rh)
Vertebrate rod photoreceptor cell
hv
Rhodopsin
Activation
MetarhodopsinII
(Interaction with Gt)
Inactivation
RK
Arr
Enzymatic regeneration
Opsin
11-cis-retinal
all-trans-retinal
Opsin
NCKX
4Na+
Ca2+
K+
CaM
Retinol metabolism in animals
Hyperpolarization
(Light response)
CNG
Closed
cGMP
Data on KEGG graph
Rendered by Pathview

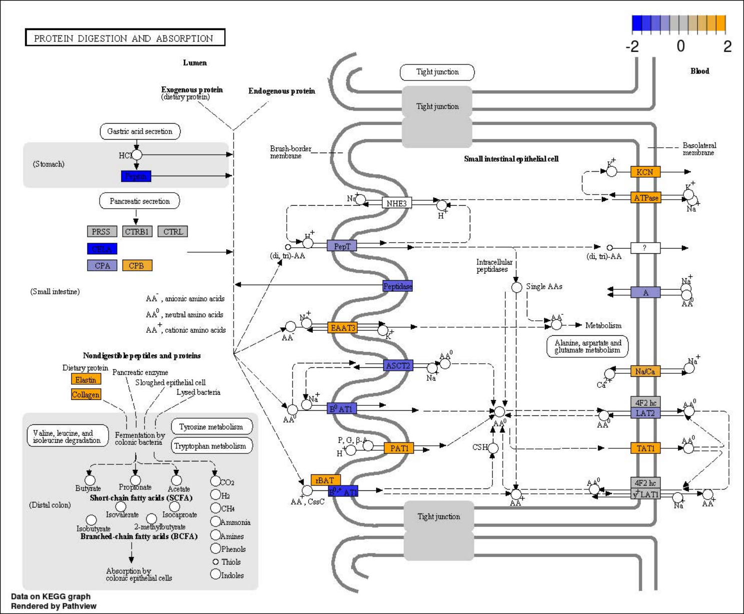
PROTEIN DIGESTION AND ABSORPTION
-2
0
2
Lumen
Blood
Exogenous protein
(dietary protein)
Endogenous protein
Tight junction
Tight junction
Tight junction
Gastric acid secretion
(Stomach)
HCl
Pepsin
Pancreatic secretion
PRSS
CTRB1
CTRL
CELA
CPA
CPB
(Small intestine)
Brush-border membrane
Basolateral membrane
Small intestinal epithelial cell
NHE3
PepT
Peptidase
EAAT3
ASCT2
B0AT1
PAT1
rBAT
B0,+AT1
KCN
ATPase
?
A
Na/Ca
4F2 hc
LAT2
TAT1
4F2 hc
y+LAT1
(di, tri)-AA
(di, tri)-AA
Intracellular peptidases
Single AAs
Metabolism
Alanine, aspartate and glutamate metabolism
AA-, anionic amino acids
AA0, neutral amino acids
AA+, cationic amino acids
P, G, β-A
CSH
AA+, CssC
Nondigestible peptides and proteins
Dietary protein
Elastin
Collagen
Pancreatic enzyme
Sloughed epithelial cell
Lysed bacteria
Valine, leucine, and isoleucine degradation
Fermentation by colonic bacteria
Tyrosine metabolism
Tryptophan metabolism
Butyrate
Propionate
Acetate
Short-chain fatty acids (SCFA)
(Distal colon)
Isovalerate
Isocaproate
Isobutyrate
2-methylbutyrate
Branched-chain fatty acids (BCFA)
Absorption by colonic epithelial cells
CO2
H2
CH4
Ammonia
Amines
Phenols
Thiols
Indoles
Data on KEGG graph
Rendered by Pathview
