# Supplementary material for: Probing the changes in gene expression due to α-crystallin mutations in mouse models of hereditary human cataract
Source: PLoS One. 2018 Jan 16;13(1):e0190817. doi: 10.1371/journal.pone.0190817 (PMC5770019; doi:10.1371/journal.pone.0190817)
Supplement: S6 Table — (DOCX) [file pone.0190817.s013.docx]

**Table S6.** Molecular weight (MW) of α-crystallin from *cryaa*-R49C and *cryab*-R120G knock-in mouse lenses.*^a^*

| Age (days) | Genotype | MW (Daltons) |
| --- | --- | --- |
|  |  |  |
| 2 | WT | 551,215 |
| 2 | *cryaa*-R49C-het | 547,817 |
| 2 | *cryaa*-R49C-homo | 714,721 |
|  |  |  |
| 14 | WT | 777,486 |
| 14 | *cryab*-R120G-het | 870,087 |
| 14 | *cryab*-R120G-homo | 974,769 |

*^a^*The molecular weights (MW) were determined by light scattering measurements from proteins separated by gel permeation chromatography. The age of the lenses was the same as the lenses analyzed by RNA-seq. MW is the weight average molecular weight. WT, Wild-type
